# Ancient regulatory evolution shapes individual language abilities in present-day humans

**DOI:** 10.1126/sciadv.aed5260

**Published:** 2026-04-22

**Authors:** Lucas G. Casten, Tanner Koomar, Taylor R. Thomas, Jin-Young Koh, Dabney Hofammann, Savantha Thenuwara, Allison Momany, Marlea O’Brien, Jeffrey C. Murray, J. Bruce Tomblin, Jacob J. Michaelson

**Affiliations:** ^1^ Department of Psychiatry, University of Iowa, Iowa City, IA 52242, USA.; ^2^ Center for Genomic Medicine, Massachusetts General Hospital, Boston, MA 02114, USA.; ^3^ Department of Otorhinolaryngology Head and Neck Surgery, School of Medicine, University of Maryland, Baltimore, MD 21201, USA.; ^4^ Carver College of Medicine, University of Iowa, Iowa City, IA 52242, USA.; ^5^ Stead Family Department of Pediatrics, University of Iowa, Iowa City, IA 52242, USA.; ^6^ Department of Communication Science and Disorders, University of Iowa, Iowa City, IA 52242, USA.

## Abstract

Language is a defining feature of our species, yet the genomic changes enabling it remain poorly understood. Despite decades of work since *FOXP2*’s discovery, we still lack a clear picture of which regions shaped language evolution and how variation contributes to present-day phenotypic differences. Using an evolutionary stratified polygenic score approach, we find that human ancestor quickly evolved regions (HAQERs) are associated with spoken language abilities (discovery *N* = 350, total replication *N* > 100,000). HAQERs evolved before the human-Neanderthal split, giving hominins increased binding of Forkhead and Homeobox transcription factors, and show evidence of balancing selection across the past 20,000 years. Language-associated variants in HAQERs appear more prevalent in Neanderthals, and HAQER-like sequences show convergent evolution across vocal-learning mammals. Our results reveal how ancient innovations continue shaping human language.

## Teaser

Ancient genomic regions shape human language through a trade-off between cognitive gains and birth complications.

## INTRODUCTION

Language is one of our species’ most remarkable cognitive innovations, yet the genetic mechanisms underlying this ability remain elusive. While the human genome differs by only 1 to 5% from our closest primate relatives (*1*–*3*), these modest genetic changes enabled the evolution of our species’ unique capacity for complex language. Understanding how these relatively small genomic differences produced profound cognitive differences represents a central challenge in evolutionary genetics, with implications for language disorders, human cognitive diversity, and the origins of human-specific traits (*4*).

The discovery that mutations in *FOXP2* cause speech and language disorders provided the first clear example of a single gene with substantial effects on language, reinforcing early expectations for simple genetic architectures (*5*, *6*). However, *FOXP2*’s contribution to typical variation in language ability proved limited, with subsequent studies failing to find associations between common *FOXP2* variants and individual differences in language skills (*7*, *8*). This limitation shifted research toward polygenic models emphasizing a large number of regulatory elements scattered throughout the genome that collectively influence language development. Genome-wide association studies have since identified numerous loci contributing to reading abilities, stuttering, rhythm, and vocabulary development, supporting a highly polygenic architecture (*9*–*14*). Cross-species studies have revealed that language-related traits (vocal learning and rhythm) show convergent evolution across mammalian lineages, with distributed regulatory networks rather than single genes controlling complex vocal behaviors (*15*–*20*). However, this polygenic model has left critical evolutionary questions unanswered: How did language-relevant regulatory elements change during human evolution, when did humans acquire language-promoting functions, and how do ancient evolutionary changes translate into present-day individual differences in language abilities?

**Fig. 1.**
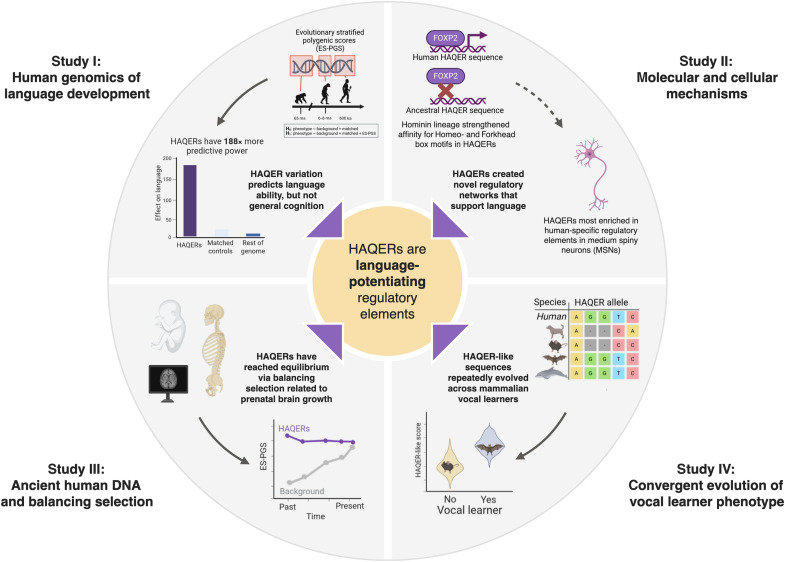
Overview of this study and key findings. ka, thousand years ago; Ma, million years ago.

To address these questions, we analyzed 65 million years of primate evolutionary history to trace the origins of language-relevant genetic variation. As part of our approach, we developed an evolutionary stratified polygenic score (ES-PGS) method that partitions genetic effects based on the evolutionary origins of their sequence context. We applied this approach across multiple datasets with detailed language phenotyping (discovery *N* = 350, total replication *N* > 100,000), combined with molecular analysis, ancient DNA analysis, and cross-species genomic comparisons. This multimodal framework enabled us to directly connect ancient genomic innovations with modern individual differences in language ability and identify neurobiological mechanisms supporting language evolution, and revealed how evolutionary trade-offs have shaped human cognitive variation. An overview of this study and key results can be seen in Fig. 1.

## RESULTS

### Dimensions of language ability

**Fig. 2.**
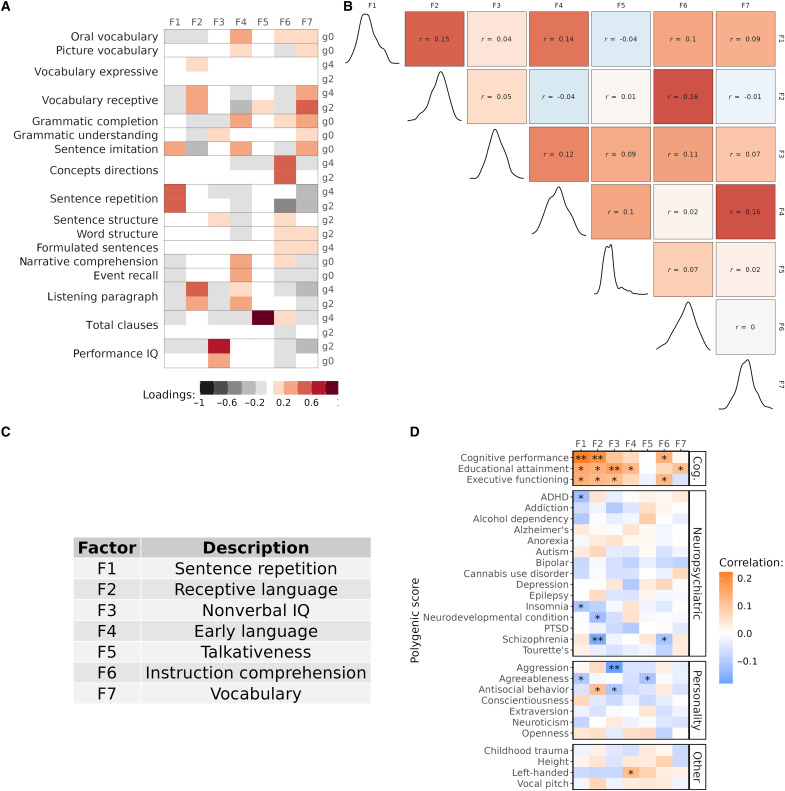
Factor loadings and genetic associations. (**A**) Loadings of cognitive and language assessments onto the seven language factors. g0 = kindergarten (age 5 to 6 years), g2 = second grade (age 7 to 8 years), and g4 = fourth grade (age 9 to 10 years). (**B**) Pearson correlations for language factors (upper triangle) and distribution of each factor (diagonal). (**C**) Interpretations of the language factors based on their loadings. (**D**) Pearson correlations for each factor with genome-wide PGS. **FDR adjusted *P* < 0.05; *unadjusted *P* < 0.05. ADHD; attention deficit-hyperactivity disorder; PTSD, post-traumatic stress disorder.

To quantify dimensions of developmental language abilities, we analyzed 17 longitudinal cognitive and language assessments administered from kindergarten through fourth grade for 350 children sampled from a community-based cohort (*21*), which we refer to as the “EpiSLI” cohort. This analysis revealed seven factors representing distinct aspects of language ability (Fig. 2A). The first factor (F1), primarily driven by spoken sentence repetition scores, represents general language ability. Sentence repetition strongly indicates overall language capacity, making F1 a key measure of general language competence (*22*, *23*). The second factor (F2) relates to receptive vocabulary and listening comprehension, covering broad receptive language skills. The third factor (F3) specifically reflects nonverbal intelligence quotient (IQ), aligning with performance IQ at both kindergarten and second grade. Factor F4 captures preliteracy language skills, incorporating all kindergarten scores except performance IQ. Its slight correlation to F1 and F2 (*r* = 0.13 and 0.12), but not F3, suggests specificity to language (Fig. 2B). Factor F5, which we call “talkativeness,” mainly reflects the number of clauses produced in a narrative task. Factor F6, based on a comprehension of concepts and directions assessment, indexes mastery of directive language (i.e., task-based instructions). Factor F7 spans a variety of assessments, with specific loading on vocabulary and grammar-related tasks, suggesting a broad, crystallized knowledge of language.

We generated high-coverage whole-genome sequencing data for all EpiSLI participants, enabling investigation of both common and rare genetic variation associated with language abilities. Most of our preliminary investigation of these factors suggested that F1, F2, and F3 carried the most genetic association signal (Fig. 2D and table S2). We also find pervasive associations with F1-F3 and measures of mental health in our sample (*N* = 241; fig. S1 and table S1).

### ES-PGS analysis

To investigate the genetic origins of language ability, we developed an ES-PGS approach that systematically examines how genetic variants from different evolutionary periods contribute to traits. ES-PGS builds on the conceptual framework of partitioned heritability and pathway-based polygenic score methods, which have successfully partitioned genetic effects across functional genomic regions (*24*–*27*). The central idea is straightforward: If language capability evolved during a particular period of human evolution, then genetic changes that occurred during that period should show stronger associations with language ability today than genetic changes from other evolutionary periods. By comparing multiple evolutionary periods, we can trace the origins of language-relevant genetic variation across deep evolutionary time. For example, if regions that differentiate modern humans from Neanderthals predict language ability better than other genomic regions, this would provide evidence that genetic changes unique to anatomically modern humans were particularly important for language evolution.

Our approach works by partitioning the genome based on these evolutionary annotations and then testing whether variants within each annotation predict language ability more strongly than would be expected by chance. Critically, we compare each evolutionary annotation not only against the rest of the genome but also against carefully matched control regions that share similar genomic properties (same size, chromosome, GC content, distance to genes, overlap with coding regions, etc.) but did not undergo the same evolutionary changes. This twofold comparison ensures that any effects we detect are specific to the evolutionary history of these regions rather than other genomic properties. By using individual-level data rather than summary statistics, ES-PGS enables direct association testing in deeply phenotyped cohorts, which is particularly valuable for specialized studies like ours with extensive language assessments.

### Human-specific genomic regions predict individual differences in language ability

To trace the evolutionary origins of language abilities, we applied ES-PGS using a cognitive performance polygenic score (CP-PGS) from a large-scale study that examined both cognitive and educational traits (*28*). This polygenic score captures general cognitive function and enables valid comparisons across multiple cognitive domains, including language and nonverbal IQ. We first confirmed that the CP-PGS showed the expected associations in our EpiSLI sample, and the genome-wide CP-PGS showed significant associations with both sentence repetition [F1, *r* = 0.22, false discovery rate (FDR) adjusted *P* = 0.001] and receptive language ability (F2, *r* = 0.19, FDR adjusted *P* = 0.01) (Fig. 2D). Last, we partitioned CP-PGS across five evolutionary annotations spanning ~65 million years of primate and human evolution, ranging from ancient primate-conserved regions to sequences differentiating modern humans from Neanderthals, to trace which genomic regions and evolutionary periods contributed to different aspects of human cognition (*29*–*33*).

**Fig. 3.**
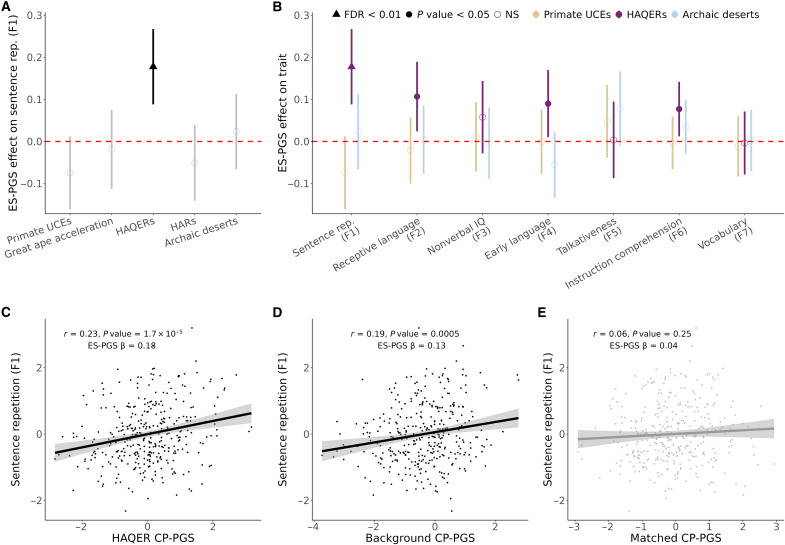
HAQERs are associated with language ability. (**A**) Comparison of evolutionary events effect on sentence repetition ability in EpiSLI (*N* = 350). Points represent the β provided from the ES-PGS models for each evolutionary annotation, while the ranges represent the 95% confidence interval (CI). Solid points indicate *P* < 0.05. (**B**) Comparison of three evolutionary (oldest, primate UCEs; middle, HAQERs; and youngest, archaic deserts) events on the seven factor scores in EpiSLI (*N* = 350). Points represent the β provided from the ES-PGS models for each evolutionary annotation, while the ranges represent the 95% CI. Solid points indicate *P* < 0.05.UCEs, ultra-conserved elements; NS, not significant. (**C**) Scatterplot of HAQER CP-PGS with sentence repetition scores (F1) in the EpiSLI sample. (**D**) Scatterplot of background CP-PGS with sentence repetition scores (F1) in the EpiSLI sample. (**E**) Scatterplot of biologically matched control regions CP-PGS with sentence repetition scores (F1) in the EpiSLI sample (matched to HAQERs).

Human ancestor quickly evolved regions [HAQERs (*31*, *34*)] emerged as the most compelling finding from this analysis. Despite comprising less than 0.1% of the human genome, HAQERs showed associations with four of the seven factors (F1, F2, F4, and F6; Fig. 3B and table S3). HAQER CP-PGS demonstrated the strongest association with sentence repetition, a measure of general language ability (F1, *r* = 0.23, ES-PGS model β = 0.18, *P* = 1.2 × 10^−4^, FDR adjusted *P* = 0.004; Fig. 3, A to C), while showing no association with nonverbal IQ (F3, ES-PGS model β = 0.06, *P* = 0.19, FDR adjusted *P* = 0.61). HAQERs are largely noncoding sequences that began rapidly evolving after the human-chimpanzee split (~6 million years ago) but before human-Neanderthal divergence (~600,000 years ago), acquiring novel regulatory functions in the human lineage (*31*, *34*).

The predictive power of HAQERs is notable. While the background and matched CP-PGS together used ~300,000 independent single-nucleotide polymorphisms (SNPs) and explained 3.7% of variance in sentence repetition ability, adding HAQER CP-PGS (comprising only 1763 independent SNPs) increased explained variance to 7.7%. This indicates that an average HAQER SNP carries 188 times more predictive power for language than SNPs elsewhere in the genome, with HAQERs alone explaining slightly more variance of sentence repetition scores in the EpiSLI cohort (*R*^2^ gain of 4%) than the remaining >99.9% of the human genome (*R*^2^ of 3.7%).

In contrast, human accelerated regions (HARs), which are deeply conserved regulatory elements that acquired human-specific changes (*32*, *35*), showed no comparable signal using ES-PGS (F1 β = −0.05, *P* = 0.27, FDR adjusted *P* = 0.7; fig. S2). This distinction suggests that human language ability emerged through novel regulatory innovations (HAQERs) rather than modifications to existing functional elements (HARs). The association between HAQERs and language factors, with no observed association with nonverbal IQ, suggests a distinct evolutionary trajectory for verbal abilities compared to general cognition. Supporting this distinction, nonverbal IQ (F3) was most strongly associated with genomic regions that underwent rapid changes across all great apes (ES-PGS β = 0.13, *P* = 0.004, FDR adjusted *P* = 0.07; fig. S2) (*30*).

### HAQERs influence language ability across multiple cohorts and variant types

**Fig. 4.**
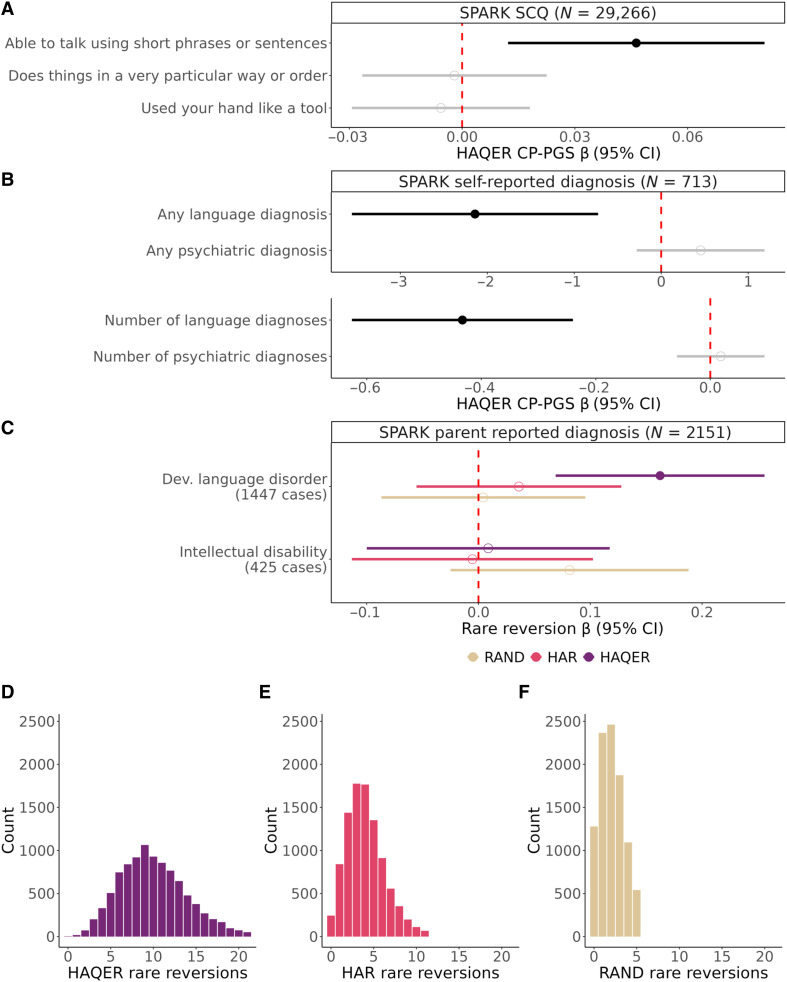
Large-scale validation of HAQERs association with language ability. (**A**) Points represent the β provided from the ES-PGS models for the HAQER CP-PGS on Social Communication Questionnaire (SCQ) items in SPARK, while the ranges represent the 95% CI. Solid points indicate *P* < 0.05. (**B**) Points represent the β provided from the ES-PGS models for the HAQER CP-PGS on self-reported language and psychiatric diagnosis in SPARK, while the ranges represent the 95% CI. Solid points indicate *P* < 0.05. (**C**) Points represent the β provided from the regression models for the rare reversions within 10 kb of HAQERs, HARs, or RAND (random matched) sequences, while the ranges represent the 95% CI. Solid points indicate *P* < 0.05. (**D** to **F**) Distributions of rare reversions counts from the SPARK whole genome sequencing data within 10 kb of the following regions: HAQERs (D), HARs (E), and random sequence (RAND; F).

We validated HAQER effects on language across multiple independent cohorts using both common and rare genetic variants. In the SPARK autism dataset (*N* > 30,000) (*36*), HAQER CP-PGS predicted verbal language capability (“able to talk using short phrases or sentences,” ES-PGS β = 0.05, *P* = 0.008, *N* = 29,266) and language disorder diagnoses in parents without autism (model improvement *P* = 7.9 × 10^−5^, *N* = 713) but not psychiatric conditions (model improvement *P* = 0.58, *N* = 713), confirming language specific effects (Fig. 4, A and B, and table S5). Clinical records showed that HAQER CP-PGS is associated with verbal IQ (ES-PGS β = 2.08, *P* = 0.022, *N* = 620) but not nonverbal IQ (ES-PGS β = 0.59, *P* = 0.49, *N* = 620; fig. S3), further supporting specificity.

To test HAQER effects through an orthogonal approach independent of our ES-PGS method, we analyzed rare genetic variations in SPARK whole genome sequencing data (*N* > 2000). We examined rare “reversions,” variants that revert from the human-specific version to their human-chimp ancestral state, reasoning that if HAQERs evolved to support human language, reversions should impair language function, which would support the observed polygenic score associations. Individuals carrying more reversions in HAQERs showed increased likelihood of developmental language disorder (β = 0.16, *P* = 6.5 × 10^−4^) and delayed language developmental milestones, but no association with age started walking or intellectual disability (Fig. 4C and table S6). Notably, HAQERs showed higher rates of reversions compared to HARs and matched random sequences (Fig. 4, D to F).

Further independent validation in the ABCD developmental cohort (*37*) showed the HAQER CP-PGS is associated with the Rey Auditory Verbal Learning Test performance, a measure of spoken word recall (ES-PGS β = 0.24, *P* = 0.048, *N* = 5625; table S7). We also tested HAQER specificity in the UK Biobank using a word reading ability polygenic score (WR-PGS) rather than our primary cognitive performance score, as the latter included UK Biobank participants in its discovery cohort (*9*, *28*). Despite the substantially smaller discovery sample for word reading genome-wide association study (GWAS) (*N* ≈ 30,000) compared to cognitive performance GWAS (*N* ≈ 250,000), the HAQER WR-PGS showed significant associations with verbal working memory (ES-PGS β = 0.01, *P* = 0.002, *N* = 84,433), a task requiring spoken recall of number sequences shown to be relevant to language (*38*), and educational attainment (ES-PGS β = 0.004, *P* = 0.005, *N* = 370,830), but not with matrix reasoning (ES-PGS β = 0.005, *P* = 0.44, *N* = 26,051), a nonverbal fluid intelligence measure (fig. S4). The detection of HAQER effects across diverse spoken language measures despite less-than-optimal validation data provides additional support for the robustness and specificity of HAQER associations with verbal abilities. These converging results across cohorts and variant types strongly support the possibility that genetic variation within HAQERs has a significant effect on spoken language abilities in contemporary humans.

### HAQERs evolved stronger binding affinity for language-relevant transcription factors

To investigate the molecular mechanisms underlying HAQERs’ association with language development, we analyzed how rare genetic variants affect transcription factor binding sites in these regions. We compared two classes of rare variants in the EpiSLI cohort: hominin-chimpanzee ancestral allele reversions versus other rare variants, using position weight matrices to quantify how these variants alter predicted transcription factor binding affinity. By comparing the effects of reversions with other rare variants, we could detect systematic evolutionary changes in hominin-specific transcription factor binding associated with language phenotypes.

**Fig. 5.**
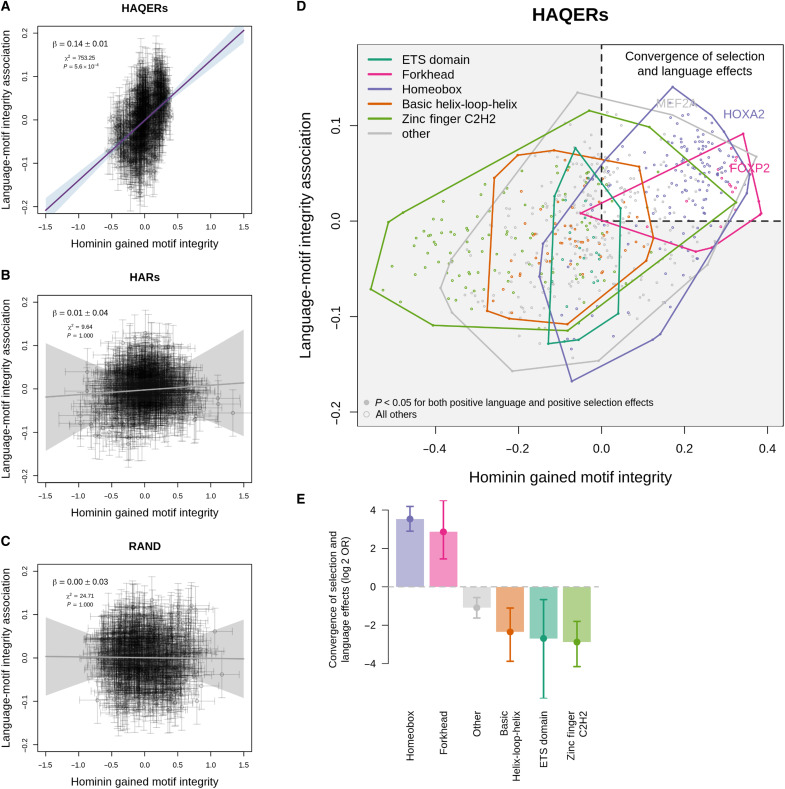
Hominin gained transcription factor binding in HAQERs influences language. (**A** to **C**) Relationship between selection for transcription factor motif integrity (*x* axis) and motif association with language ability (*y* axis) in (A) HAQERs, (B) HARs, and (C) random genomic regions. Each point represents one transcription factor motif. Error bars indicate ±1 standard error. Purple line (or gray for nonsignificant fits) shows York regression fit with 95% CI (shaded); regression coefficient (β), chi-squared statistic (χ^2^), and *P* values are shown. (**D**) Detailed view of motif effects in HAQERs colored by transcription factor family. Solid points indicate motifs with *P* < 0.05 for both positive selection and positive language association. Colored polygons show convex hulls for each transcription factor family. (**E**) Enrichment analysis of transcription factor families for concordant positive selection and language effects, shown as log 2 odds ratio (OR). Error bars indicate 95% CIs. Solid points indicate *P* < 0.05. ETS, E26 transformation-specific.

In HAQERs, hominin-gained transcription factor motif binding showed significant correlation with individual sentence repetition ability (*N* = 350 individuals, β = 0.14, *P* = 5.6 × 10^−4^; Fig. 5A), suggesting that hominins evolved increased transcription factor binding affinity in these regions that may support spoken language abilities. In contrast, regions under sequence conservation with human-specific changes (HARs, β = 0.01, *P* = 1; Fig. 5B) or neutral evolution (RAND sequences, β = 0, *P* = 1; Fig. 5C) showed no relationship between motif integrity and language ability, highlighting HAQERs’ unique selection for regulatory function during hominin evolution.

Analysis of specific transcription factor families revealed notable enrichment of Homeobox and Forkhead box transcription factors associated with both enhanced binding affinity in HAQERs and improved language performance (Fig. 5D). The Homeobox family displayed the strongest enrichment among all transcription factor families (odds ratio = 11.58, *P* = 2.6 × 10^−34^), followed by the Forkhead box family (which includes *FOXP2*, odds ratio = 7.28, *P* = 5.3 × 10^−6^; Fig. 5E and table S16). These results suggest that hominin-gained binding of Homeobox and Forkhead box families within HAQERs may have played a crucial role in the evolution of human language capability.

### HAQERs regulate language-relevant brain cell types through human-specific chromatin accessibility

To determine which types of brain cells are regulated by HAQERs, we analyzed their overlap with candidate cis-regulatory elements (cCREs) identified by single-nucleus chromatin accessibility profiling (single-nucleus assay for transposase-accessible chromatin) of human and mouse brain cell types (*39*). We tested whether HAQERs preferentially associate with human-specific versus evolutionarily conserved chromatin accessible regions, reasoning that HAQERs should show increased overlap with human-specific regulatory elements if they provide novel functions in the human lineage.

HAQERs demonstrated significant enrichment for human-specific cCREs across brain cell types (fig. S7A), with the strongest enrichment in medium spiny neurons (MSNs, *P* = 9.8 × 10^−8^). MSNs comprise over 90% of neurons in the human striatum, a circuit that plays essential roles in vocal learning across species (*17*, *40*) and was the only part of the brain robustly associated with developmental language disorders in a recent meta-analysis (*41*). HAQERs also showed enrichment around human-specific cCREs in *FOXP2*-expressing neurons (*P* = 5.3 × 10^−4^), providing independent evidence linking HAQERs to *FOXP2* regulatory networks beyond our transcription factor binding findings (Fig. 5E). In contrast, HARs showed minimal enrichment for human-specific chromatin accessibility but strong enrichment for evolutionarily conserved regions (strongest in VIP neurons, *P* = 2.7 × 10^−10^; fig. S7B). These results are consistent with HAQERs providing novel regulatory functions specific to human brain development that may support language capabilities.

### Selective pressures acting on language and general cognition

**Fig. 6.**
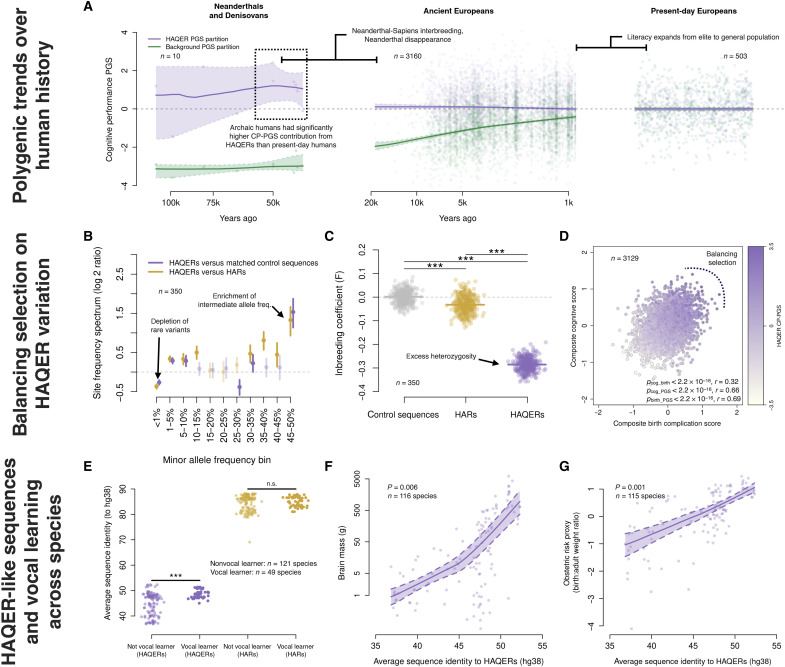
Selective pressures on human cognition and convergent evolution of vocal learning. (**A**) CP ES-PGS across hominin evolution. HAQER PGS (purple) and background PGS (green) plotted against sample age for Neanderthals/Denisovans, ancient Europeans, and present-day Europeans. Locally estimated scatterplot smoothing (LOESS) fits with 95% CIs shown. (**B**) Site frequency spectrum comparing HAQERs to matched controls and HARs. Log 2 ratio of proportion of variants across allele frequency bins; positive values indicate HAQER enrichment. Error bars, 95% bootstrap CIs. (**C**) Inbreeding coefficient (*F* statistic) across sequence types. Lower *F* statistics indicate excess heterozygosity. HAQERs show significantly lower *F* statistics than HARs and control regions (***P < 0.001). (**D**) Canonical correlation analysis linking HAQER CP-PGS to cognitive and birth complication traits in the ABCD cohort. Each point is an individual, colored by their HAQER CP-PGS. *x* axis represents a composite birth complication score; *y* axis captures cognitive performance measures (see fig. S6 for variable loadings). (**E**) HAQER-like and HAR-like sequence similarity scores in nonvocal learning (*N* = 121 species) and vocal learning (*N* = 49 species) mammals. Phylogenetic logistic regression statistics are shown. n.s., not significant. (**F** and **G**) HAQER-like sequence similarity correlates with brain mass [*N* = 116 species; (F)] and birth:adult weight ratio [*N* = 115 species; (G)] across mammals. LOESS fits with 95% CIs.

Having multiple lines of evidence associating HAQERs with human language evolution, we next examined how selective pressures may have influenced language-related genetic variation over the past 20,000 years of human history using the Allen Ancient DNA Resource (AADR) (*42*). The AADR is the largest genotyped collection of ancient humans, providing harmonized genotype and metadata for each sample (like radiocarbon dating–based sample ages). We identified ancient west Eurasians, then correlated their HAQER CP-PGS and the background CP-PGS with sample age (*N* = 3244 individuals with remains dated between 18,775 and 150 years ago passing quality control). We see that the polygenic score for general cognition (background CP-PGS) has been subject to positive selection and has increased substantially over time (selection coefficient = 0.088, *P* = 2.1 × 10^−12^; Fig. 6A). Unexpectedly, we found that HAQER CP-PGS has been stable throughout human history, indicating that ancient and modern humans carry similar numbers of language-related alleles in HAQERs (selection coefficient = −0.004, *P* = 0.71; Fig. 6A).

The presence of HAQERs in archaic humans provided a unique opportunity to describe genetically predicted cognitive traits across human species. To do this, we computed HAQER CP-PGS and background CP-PGS in archaic humans (*N* = 10) and compared them to ancient (*N* = 3,244) and modern humans (*N* = 503). The 10 archaic human genomes (eight Neanderthals and two Denisovans) showed elevated HAQER CP-PGS (mean *z*-score = 0.91, median *z*-score = 1.23), while having reduced background CP-PGS (mean *z*-score = −3.02, median *z*-score = −3.01; Fig. 6A). In contrast, a set of random matched control regions showed no differences in CP-PGS across groups (fig. S5, A to C, and table S9). While these data should be interpreted with caution due to challenges applying polygenic scores across populations (*43*), the elevated HAQER CP-PGS we observed aligns with arguments that archaic humans were capable of complex language (*44*–*46*).

### Evidence of balancing selection from modern genomes

The stability of HAQER CP-PGS throughout human evolutionary history led us to hypothesize that HAQERs have been maintained through balancing selection. Multiple population genetic analyses in the EpiSLI sample provided support for this hypothesis. HAQERs exhibited significantly more heterozygosity compared to both HARs (*t* statistic = 110, *P* = 5.3 × 10^−273^) and matched control sequences (*t* statistic = 147, *P* = 1.6 × 10^−315^), suggesting that heterozygosity at HAQER loci provided a selective advantage (Fig. 6C). In addition, we observed an enrichment of intermediate frequency variants (minor allele frequencies between 30 to 50%) in HAQERs (Fig. 6B). These patterns could arise from several mechanisms, including elevated mutation rates or balancing selection (*47*, *48*). However, together with the stability of HAQER polygenic scores over 20,000 years and the obstetric trade-offs (described below), our findings are most consistent with balancing selection maintaining genetic variation in these regions at intermediate frequencies throughout human history.

### HAQERs influence prenatal brain development

The evolutionary analysis revealed a puzzling pattern: while other cognitive variants show recent positive selection, the HAQER CP-PGS has remained stable for at least the past 20,000 years of human history despite the cognitive benefits associated with these variants. This stability suggests ongoing fitness costs that counterbalance the advantages of increased language ability. Given HAQERs’ established role in neurodevelopment (*34*), we investigated whether these variants create pleiotropic effects on prenatal brain development that could explain their evolutionary constraints. First, we investigated temporal and cell-type enrichment for these regions to identify if they were likely to influence birth-related neurodevelopmental traits. HAQERs showed broad enrichment for variants affecting prenatal brain gene expression when intersected with single-cell quantitative trait loci (scQTLs) from developing midbrain neurons (*49*). The strongest enrichment observed was at the late prenatal time point, which corresponds to when the human brain most rapidly expands (fig. S7C) (*50*). Critically, HAQERs showed no enrichment when we examined adult brain regulatory elements (*51*), confirming prenatal neurodevelopmental effects (fig. S7D). HAQERs were also significantly enriched around genomic loci associated with head circumference at birth, a proxy for brain size (*P* = 4.4 × 10^−4^; fig. S7E) (*52*).

### HAQERs link language evolution to the obstetric dilemma

The evidence for HAQER effects on prenatal brain development and head circumference at birth suggests a potential mechanism for their evolutionary stability: the obstetric dilemma. Enhanced fetal brain development may create reproductive costs through the obstetric dilemma, where increased brain size complicates birth in bipedal humans with narrow pelvises (*53*, *54*). To test whether HAQERs contribute to this trade-off, we analyzed brain imaging, cognitive, and birth outcome data in the ABCD cohort (*37*).

A canonical correlation analysis revealed two distinct composite phenotypic axes associated with HAQER CP-PGS, providing evidence for the obstetric dilemma that could plausibly drive the observed balancing selection (Fig. 6D). The first canonical component captured variance primarily from birth complication–related variables, while the second component loaded predominantly on cognitive performance measures (including a measure of verbal language learning; fig. S6). Critically, both composite scores showed positive correlations with HAQER CP-PGS (*r* = 0.69 and *r* = 0.66, respectively; *P* < 2.2 × 10^−16^ for both), and the two composite scores were themselves positively correlated (*r* = 0.32, *P* < 2.2 × 10^−16^). This pattern indicates that genetic variants contributing to higher HAQER CP-PGS simultaneously increase both cognition (a trait under positive selection) and birth complication risk (a trait under negative selection). The consistent positive relationship between HAQER CP-PGS and both phenotypic domains provides a plausible mechanistic explanation for the balancing selection signatures specific to HAQERs. While the correlation magnitudes should be interpreted cautiously given our optimization procedure (see Materials and Methods), the qualitative finding of antagonistic pleiotropy is robust and aligns with the evolutionary hypothesis that the cognitive benefits conferred by HAQER variants are counterbalanced by obstetric costs, maintaining genetic diversity at these loci through balancing selection. This trade-off between cognitive ability and increased birth complications is a fundamental constraint that may have shaped the evolution of human language.

### HAQER-like sequences show convergent evolution in vocal learning mammals

To test whether HAQER functions extend beyond humans, we analyzed homologous sequences across 170 nonprimate mammalian species, including 49 vocal learners and 121 nonvocal learner species (*15*). Vocal learner species can acquire and modify vocalizations through experience, contrasting with species restricted to innate vocalizations. We computed genome-wide “HAQER-like” and “HAR-like” sequence similarity scores for each species by comparing homologous genomic regions to the human reference genome using whole genome alignment data (*55*), with these scores measuring how similar each species’ sequences are to human HAQERs and HARs. We tested for associations between these similarity scores and vocal learning ability while controlling for phylogenetic relatedness across species (*56*, *57*).

Vocal learner species showed significantly higher HAQER-like sequence similarity compared to nonvocal learners (phylogenetic regression β = 1.41, *P* = 1 × 10^−4^; Fig. 6E). HAQERs were also enriched around previously identified mammalian vocal learner enhancer regions (*P* = 0.028; fig. S7F) (*15*). While HARs showed a similar enrichment around vocal learner enhancer regions (*P* = 0.04), HAR sequence similarity was not associated with vocal learner classification (phylogenetic regression β = −0.15, *P* = 0.67; Fig. 6E). Given the independent evolution of vocal learning across mammalian lineages, these results suggest that HAQER-like sequences may be a fundamental genetic mechanism for complex vocal communication that has been repeatedly used across evolutionary history.

HAQER-like sequences also associated with brain size across species (phylogenetic regression β = 0.42, *P* = 0.006, *N* = 116 species) and larger relative birth weights (phylogenetic regression β = 0.44, *P* = 0.001, *N* = 115 species), mirroring the human obstetric dilemma pattern (Fig. 6, F and G). This convergent evidence across independent evolutionary lineages supports a link between the genetic architecture of vocal learning, brain development, and reproductive constraints. Together, these results suggest that the trade-offs we observe in human language evolution may represent a broader biological phenomenon that support complex vocal communication.

## DISCUSSION

Our ES-PGS analysis identifies genomic regions that disproportionately contributed to human language evolution and continue to influence individual differences in language abilities observed in present-day humans. While previous research demonstrated that rare mutations in *FOXP2* can cause language disorders (*5*, *58*), common variants in *FOXP2* show minimal association with typical language variation (*7*, *8*), prompting GWAS studies and polygenic models of language-related traits (*9*, *11*–*14*, *59*). However, these models left critical questions unanswered: When did language-associated variation evolve, and how do these molecular changes influence language development? Our analysis reveals that HAQERs (*31*, *34*), mostly noncoding regions that rapidly evolved before the human-Neanderthal split and represent less than 0.1% of the human genome, harbor variants with disproportionate effects on language. Individual SNPs in HAQERs carry 188 times more impact on language than variants elsewhere in the genome. While we observed consistent associations between HAQERs and language-related measures across multiple independent cohorts, the relatively modest replication effect sizes (UK Biobank, *P* = 0.002; minimum SPARK, *P* = 7.9 × 10^−5^; and ABCD, *P* = 0.048) likely reflect phenotypic heterogeneity, with the EpiSLI discovery cohort undergoing extensive, standardized language assessments compared to briefer measures in replication samples. In contrast, we observed no association between HAQERs and nonverbal cognition across multiple independent cohorts, although continued investigation of HAQER effects across diverse cognitive domains will be valuable for understanding the full extent of their phenotypic specificity. These language-specific effects, distributed across thousands of small regulatory elements, support the polygenic architecture of human language while establishing HAQERs as crucial connectors between the evolutionary emergence of human language capacity and ongoing individual differences in language ability.

We find that HAQERs evolved across hominins to increase binding for Forkhead (including *FOXP2*) and Homeobox transcription factors (TFs), with motif integrity correlating with individual language scores. Supporting this regulatory mechanism, HAQERs show significant enrichment for human-specific chromatin-accessible regions across brain cell types, with strong enrichment in MSNs and *FOXP2*-expressing neurons, while HARs primarily overlap with evolutionarily conserved regulatory elements. This suggests that *FOXP2* influences language primarily through its regulatory networks (*60*) rather than protein-coding changes, explaining how rare mutations in a single TF can produce profound effects on language development while common variants in the same TF shows minimal associations (*7*, *8*). The observed 11.58-fold and 7.28-fold enrichment of hominin-gained Homeobox and Forkhead binding sites that positively correlate with language scores in HAQERs suggests that these developmentally essential TF families may have played a central role in human language evolution (*61*–*63*).

Cross-species triangulation provides independent support that HAQERs are functional regulatory elements for language-related traits. Consistent with our human evidence, vocal learner mammals show significantly higher HAQER-like sequence similarity than nonvocal learners after controlling for phylogenetic relationships, with parallel associations for brain size and birth weight. HAQERs show enrichment around established mammalian vocal learning enhancer regions (*15*), consistent with previous reports of convergent evolution for complex vocal communication (*15*–*20*). This convergent evolution of HAQER-like sequences across vocal learning lineages provides strong independent support that these regulatory elements are fundamental genetic mechanisms for complex vocal communication.

Ancient DNA analysis reveals that while general cognitive variants show positive selection over 20,000 years, the language-related HAQER polygenic score has remained stable, suggesting that balancing selection maintains genetic variation in these language-relevant regions. Analysis of birth outcomes, brain imaging, and cognitive data provided a potential mechanism for this unexpected evolutionary constraint: Individuals with higher HAQER CP-PGS were more likely to have larger heads and birth complications, indicating trade-offs between language capability and reproductive costs. This pattern connects HAQERs to the obstetric dilemma, the evolutionary trade-off between narrower pelvises supporting upright walking and larger fetal brains enabling complex cognition (*53*, *54*), potentially explaining why variants in HAQERs persist at intermediate frequencies rather than reaching fixation. Further supporting this neurodevelopmental mechanism, we find that HAQERs are enriched for genetic variants associated with prenatal gene expression and head circumference at birth (*49*, *52*). The prenatal brain regulatory activity of HAQERs aligns with established evidence that early developmental processes critically influence later language outcomes (*64*, *65*). Despite substantial methodological limitations in cross-population polygenic score applications (*43*), the available Neanderthal and Denisovan genomes show higher HAQER CP-PGS than modern humans, although this requires careful interpretation. This finding needs further validation, and future research should also investigate morphological and obstetric differences between archaic and modern humans that may have enabled archaic populations to maintain higher HAQER polygenic scores. These results point to the obstetric dilemma as an ongoing evolutionary constraint that specifically limits language-related genetic variation from reaching fixation, creating a fundamentally different selection landscape for vocal communication compared to general cognitive abilities.

These findings demonstrate how ancient regulatory innovations continue to shape human language variation through evolutionary constraints that balance cognitive benefits against reproductive costs. The independent evolution of HAQER-like sequences in vocal learning mammals reveals fundamental genetic mechanisms for complex communication that have emerged repeatedly across lineages. While general cognitive variants show positive selection over time, language variants remain stable at intermediate frequencies, suggesting that individual differences in spoken language abilities reflect ongoing evolutionary trade-offs. These evolutionary constraints reveal why individual differences in language abilities persist despite the importance of communication skills. Further investigation of HAQER regulatory networks will be essential to translate these evolutionary insights into approaches for supporting those with language disorders.

## MATERIALS AND METHODS

We developed an ES-PGS approach to trace the origins of language-relevant genetic variation across 65 million years of primate evolutionary events. Language abilities were assessed through factor analysis of 17 longitudinal cognitive and language assessments from kindergarten through fourth grade in 350 children from a community-based cohort (EpiSLI) (*21*). We applied ES-PGS using cognitive performance (*28*) polygenic scores partitioned across five evolutionary annotations (*29*–*33*), testing whether genomic regions from specific evolutionary periods contribute disproportionately to language versus general cognitive functions.

Validation was performed across multiple independent cohorts using both common and rare genetic variants. In the SPARK autism dataset (*N* > 30,000) (*36*), we tested HAQER effects on verbal language ability and language disorder diagnoses. We analyzed rare “reversions” in SPARK whole genome sequencing data (*N* > 2,000 individuals), variants reverting from human-specific to human-chimpanzee ancestral states (*34*), reasoning that if HAQERs evolved to enhance language, reversions should impair language function. Additional validation used unrelated individuals from the ABCD cohort (*N* = 5625) for spoken word recall performance, cognitive test scores, brain size, and birth traits (*37*). Last, we validated the effects of HAQERs on language-related traits in the UK Biobank (*66*). Given that cognitive trait GWASs use the UK Biobank, we opted to use the largest quantitative language-related GWAS available (for word reading) in our ES-PGS analysis (*9*). We focused our analysis on a verbal working memory trait, which was previously shown to be highly relevant to language, and educational attainment (*38*). For comparison, we also analyzed matrix reasoning in the UK Biobank.

To investigate molecular mechanisms supporting language evolution, we analyzed how rare genetic variants in evolutionarily significant regions affect transcription factor binding sites using position weight matrices for 633 human transcription factors from JASPAR2020 (*67*). We compared hominin-chimpanzee ancestral allele reversions versus other rare variants to detect systematic evolutionary changes in transcription factor binding associated with language phenotypes. In addition, we looked for enrichment of evolutionarily significant regions across human-specific or conserved chromatin accessible regions (*39*), birth head circumference GWAS loci (*52*), neurodevelopmental related scQTL datasets (*49*, *51*), and mammalian vocal learning genomic regions (*15*).

Ancient DNA analysis examined selective pressures using the AADR (*42*), correlating HAQER polygenic scores with sample age in 3244 ancient west Eurasians (18,775 to 150 years ago). We computed polygenic scores in archaic humans (8 Neanderthals and 2 Denisovans) and compared them to ancient and modern humans. Cross-species validation analyzed HAQER-like sequence similarity across 170 nonprimate mammalian species (49 vocal learning and 121 nonvocal learning) (*15*) using phylogenetic regression to control for evolutionary relatedness (*56*, *57*) in analyses of vocal learning, brain size, and a birth:adult weight ratio (used as a rough proxy for the obstetric dilemma) (*68*).

## Supplementary Materials

### The PDF file includes:

Supplementary Materials and Methods Figs. S1 to S7 Legends for tables S1 to S18 References
