# Supplementary material for: Ancient regulatory evolution shapes individual language abilities in present-day humans
Source: Sci Adv. 2026 Apr 22;12(17):eaed5260. doi: 10.1126/sciadv.aed5260 (PMC13101878; doi:10.1126/sciadv.aed5260)
Supplement: Supplementary file 1 — Supplementary Materials and Methods Figs. S1 to S7 Legends for tables S1 to S18 References [file sciadv.aed5260_sm.pdf]

Science Advances
AAAS

Supplementary Materials for

Ancient regulatory evolution shapes individual language abilities in present-day humans

Lucas G. Casten *et al.*

Corresponding author: Jacob J. Michaelson, jacob-michaelson@uiowa.edu

Sci. Adv. **12**, eaed5260 (2026)
DOI: 10.1126/sciadv.aed5260

The PDF file includes:

Supplementary Materials and Methods
Figs. S1 to S7
Legends for tables S1 to S18
References

Other Supplementary Material for this manuscript includes the following:

Tables S1 to S18

Materials and Methods

Human Subjects Consent and IRB Approval

All EpiSLI subjects in this study were minors who assented to participation under the University of Iowa IRB# 200511767. Secondary genetic analyses for EpiSLI subjects were approved and carried out under the University of Iowa IRB #201406727. SPARK is approved under WIRB #201703201. Approval for data collection and analysis of the SPARK Research Match study described here was conducted under the the University of Iowa IRB #201705739. This research has been conducted using the UK Biobank Resource under Application Number #46053.

EpiSLI Cohort

The discovery cohort of this study, referred to as EpiSLI, was originally recruited as an epidemiological study of language impairments in Kindergarteners in the state of Iowa. A more detailed description of the recruitment scheme can be found in the initial (*69*), and subsequent (*21, 70*) publications. In brief, 7,218 kindergarteners, sampled to be representative of Iowa's population were screened for language impairment with a rapid, 40-item subset (*69*) of the TOLD-2P (*71*). Children who failed the TOLD-2P screener (i.e., have poor language ability) were over-sampled to capture a broad range of language ability in the primary cohort (n = 1,929 children). Children with autism spectrum disorder and/or intellectual disability were excluded. The entirety of the primary cohort received a more complete battery of language and cognitive assessments, while teachers and parents completed scholastic and behavioral questionnaires about the children 2nd, 4th, 8th, and 10th grades. Language and cognitive assessments included: TOLD-2P (*71*), WPPSI (*72*), a narrative story task (*73*), Woodcock Reading Mastery Tests-Revised (*74*), Word-Sound Deletion task (*75*), Random Animals-Colors task (*75*). We selected a total of 390 children from the primary cohort for whole genome sequencing. This sample represents a broad spectrum of language abilities, these 390 children were chosen by sampling from the tails of the distribution of a composite language score inward until we reached our final sample size (*8, 69*).

Behavioral assessment scores came from normed T-scores for the summary scales of the Child Behavior Checklist (CBCL) developed by ASEBA (*76*). CBCLs were completed at the 2nd grade timepoint of the study (241 of the 350 children used in this study had complete CBCL data for the

analysis in Supplementary Table 2).

Factor Analysis

An exploratory maximum-likelihood factor analysis was carried out on the language and cognitive assessment scores using the `factanal` function from the `stats` package in R (*77*), with the default "varimax" rotation. A number of factors from 1-10 were evaluated, with 7 - the largest number of factors with a nominal χ^2 p-value less than 0.05 - chosen for subsequent analyses. These seven factors accounted for an estimated 62.3% of the total variance in the original data.

EpiSLI Cognitive measures

Language assessment scores and IQ were normalized via the general scheme described in (*69*) to account for the over-sampling of individuals with low language ability. Scores were corrected for age as the residual of a linear regression model using `lm()` in R (*77*). The age-corrected language scores were then normalized with respect to the population using the `wtd.mean()` and `wtd.var()` functions from the `Hmisc` package (*78*) in R.

Cognitive data Imputation

Most individuals for whom whole genome sequencing was generated had scores from assessments given in 2nd grade, while a minority had only 4th grade (41/390) or kindergarten (39/390) scores. Missing scores were imputed using chained random forests and predictive mean matching as implemented the `missRanger` R package (*79*) with 5,000 trees and `pmm.k = 20`.

EpiSLI Whole Genome Sequencing

Sample Collection and whole genome sequencing

DNA was collected and extracted from either blood or saliva samples for 390 individuals (350 of which were used in the final analysis after quality control). DNA concentration of all sequenced samples was quantified with with Qubit 2.0 Fluorometer (Life Technologies Corporation). 390 DNA samples were sheared on a E220 Focused-ultrasonicator (Covaris) to an average size of 400 bp. Sequencing libraries were generated with a Kapa Hyper Prep kit (Kapa Biosystems) according

to the manufacturer protocol. All samples had an average genome-wide coverage of at least 20X and were sequenced on a HiSeq4000 (Illumina) with 150-bp Paired End chemistry.

Post-sequencing QC

All sequencing data was analysed with Fastqc (v0.11.8) (*80*), where no samples failed the run modules. After genome alignment (see below), some samples were found to have significantly better coverage than others, in excess of 40x. To help control for ascertainment bias in these samples, all samples with an average coverage > 35x (based on initially mapped BAM) were randomly down-sampled, leading to the distribution of genome-wide coverage and average insert-size outlined in Supplementary Table 17.

Genome Alignment and Variant Calling

Reads were processed with bcbio (v1.1.6) (*81*) and mapped to hg19 via BWA-mem (v0.7.17) (*82*). SNVs and InDels were then called with all samples in a pool with three variant callers: GATK (v4.1.2.0) (*83*), FreeBayes (v1.1.0.46) (*84*), and Platypus (v0.8.1.2) (*85*).

Variants from each caller were filtered according to caller-specific quality metrics. For GATK, variants needed to pass all VQSR tranche thresholds. For Platypus, variants were filtered based on the goodness of fit of genotype calls, excessive region-based haplotype scores, root-mean-square mapping quality, variant quality and its ratio with read depth, low complexity sequence context, allele bias, region-based read quality, neighboring homopolymers, and strand bias. For FreeBayes, variants were filtered based on a combination of allele frequency, read depth, and overall quality. The thresholds used for filtering Platypus and FreeBayes calls were the default set by bcbio. An ensemble of all three callers was generated and used in all subsequent analyses in order to achieve improved specificity in the detection of rare variants. All variants in the ensemble callset were called by GATK as well as either Platypus OR FreeBayes. The majority of variants (87%; 25,775,508) were called by all three callers, while a minority were called by GATK and only one other caller (13%; 3,937,146). All variants were filtered to have a QUAL and individual quality score ≥ 20. Qualified researchers can access the individual level genotypes from dbGaP at (study accession = phs002255.v1.p1): `https://www.ncbi.nlm.nih.gov/projects/gap/cgi-bin/study.cgi?study_id=phs002255.v1.p1`

Final sample QC

Starting from the initial sample size of 390, a total of 14 samples were flagged for removal from all association analyses because of population stratification. Specifically, these samples did not cluster with 1,000 Genomes Europeans (*86*), or were more than 3 standard deviations away from the rest of the EpiSLI cohort based on the top 10 multidimensional scaling components calculated from SNPs found at or above a 0.05 minor allele frequency. An additional 26 samples were dropped due to relatedness or limited phenotypic data, leaving a final sample size of 350 unrelated European individuals with complete data for all genomic analyses.

Variant Annotations

Variants were annotated with the Ensembl Variant Effect Predictor tool (VEP v109) (*87*). Reference population allele frequencies came from 1000 Genomes Phase 3 samples (*86*) and GnomAD (*88*), rsIDs of variants were dbSNP (*89*) (v151), these additional annotations were added using VCFanno (*90*) (v20190119).

Polygenic Scores (PGS)

Genotypes for PGS

To derive a set of SNPs suitable for PGS analysis, we merged our dataset with 1000 Genomes Europeans to use as a reference sample. Based on widely used recommendations (*91*), we extracted SNPs with a minor allele frequency ≥ 1%, Hardy-Weinberg equilibrium p-value > 1×10^{-6}, and a missingness rate < 2% in both samples (leaving 7,719,665 SNPs total for PGS calculation).

PGS Calculation

LDpred2 was used to calculate a genome-wide PGS for all traits with the infinitesimal model using the provided UK Biobank LD reference panel and HapMap3+ variant set (*92*). PGS were calculated using GWAS summary statistics for neurodevelopmental and psychiatric traits: ADHD (*93*), addiction (*94*), alcohol dependency (*95*), Alzheimer's (*96*), Autism (*97*), Anorexia (*98*), bipolar disorder (*99*), cannabis use disorder (*100*), depression (*101*), epilepsy (*102*), insomnia (*103*), neurodevelopmental conditions (*104*), PTSD (*105*), schizophrenia (*106*), and Tourette's (*107*).

GWAS summary statistics for cognitive traits included: cognitive performance (*28*), educational attainment (*108*), executive functioning (*109*), and the "g Factor" (*110*). Additional PGS were calculated for the following behavioral and socioeconomic status related traits: childhood aggression (*111*), antisocial behavior (*112*), empathy (*113*), the BIG5 personality traits (*114*), income (*115*), and the Townsend Deprivation Index (a measure of material deprivation) (*116*). PGS were also calculated for brain structural (*117, 118*) and functional connectivity phenotypes (*119*). Finally, we computed PGS for miscellaneous traits: left-handedness (*116*), height (*120*), childhood trauma (*121*), and vocal pitch (*122*). It is important to note we did not compute PGS in EpiSLI for reading based traits, because our sample was part of the discovery cohort of the largest reading related GWAS to date (*9*). Associations for all of these PGS with our language factors can be found in Supplementary Table 3.

To account for population stratification, we corrected PGS for the first 5 genetic principal components. PGS were normed to the 1000 Genomes Europeans reference sample.

ES-PGS Calculation

We developed evolutionary stratified polygenic scores (ES-PGS) to identify genomic regions from specific evolutionary periods that disproportionately contribute to modern phenotypic variation. ES-PGS partitions polygenic score effects based on evolutionary annotations, testing whether variants from particular evolutionary epochs show disproportionately strong associations with traits compared to the broader genome.

The method uses three components: (1) annotation-specific PGS calculated from variants within evolutionary regions of interest, (2) background PGS calculated from all remaining genome-wide variants, and (3) matched control PGS from biologically similar regions to test specificity. Matched control regions were generated by sampling 1,000 random genomic segments for each annotation region (e.g., 10 annotation region would produce 10,000 random regions for matching), these regions are then matched for chromosome, size, GC content, repeat content, distance to nearest gene, and overlap with promoter or coding sequences. Control regions were required to be $\geq$ 100kb from any annotation region to ensure independence.

We implemented ES-PGS using nested model comparisons. The reduced model tests background genomic effects and matched control effects:

$$y = \beta_0 + \beta_1 \text{PGS}_{\text{background}} + \beta_2 \text{PGS}_{\text{matched}} + \epsilon \tag{S1}$$

The full model adds the annotation-specific term:

$$y = \beta_0 + \beta_1 \text{PGS}_{\text{background}} + \beta_2 \text{PGS}_{\text{matched}} + \beta_3 \text{PGS}_{\text{annotation}} + \epsilon \tag{S2}$$

Where y represents the phenotype (e.g., language factor scores), $\text{PGS}_{\text{background}}$ includes all variants except those in the focal annotation, $\text{PGS}_{\text{matched}}$ represents the matched control regions, and $\text{PGS}_{\text{annotation}}$ contains only variants within the evolutionary annotation. An ANOVA comparing these models tests whether the annotation contributes significantly beyond background genetic effects and matched controls. Additional covariates (genetic principal components, demographics) can be incorporated as needed.

PRSet (*27*) was used to calculate stratified PGS using human genome annotations in BED formatted files, computing a clumping and thresholding based ES-PGS for each annotation and a background PGS for the rest of the genome. Background regions for each annotation were identified using the "complement" function from bedtools (v2.26.0) (*123*). Matched control regions were matched using the `nullranges` R package (*124*). As recommended by the authors of PRSet, we used a p-value threshold of 1 and the 1000 Genomes Europeans as the LD reference (*86*). We corrected for population stratification using the same steps as described in the genome-wide PGS analysis.

We focused our analysis on genome annotations related to primate and human evolution, annotations included: primate ultra conserved regions (primate UCEs, Supplementary Data 5 from the reference) (*29*), great ape lineage accelerated regions (*30*)(Supplementary Data S1 filtered to "Hominidae" from the reference), Human Ancestor Quickly Evolved Regions (HAQERs, Supplementary Table 68 from the reference) (*31*), Human Accelerated Regions (HARs) (*32*), and ancient human selective sweep regions (Supplemental File S2 from the reference) (*33*). All annotations were converted to BED format and lifted over to match genome builds when necessary, using the UCSC liftOver tool (*125*). All annotation coordinates, complement regions, and matched regions can be found in our repository:
`https://github.com/lucasgcasten/language_evolution/tree/master/manuscript/supplemental materials/ES-PGS_annotations`

ES-PGS replication in SPARK

To validate our ES-PGS results in a separate sample we used the SPARK cohort, a large genetic study of individuals with autism and their family members (*36*). For the replication, we utilized imputed SNP array data that we have previously described (*126*) to compute an ES-PGS using the same workflow as in our EpiSLI sample. The self-reported language and psychiatric diagnosis phenotypes, came from a questionnaire as part of a previously described research match where we had > 1,000 adults with autism or parents of children with autism complete an online language battery (*127*). We grouped all self-reported language-related diagnoses (language impairment, dyslexia, hearing impairment, stuttering, and speech impediments), and psychiatric diagnoses together (major depression, generalized anxiety disorder, bipolar, schizophrenia, substance abuse, OCD, and Tourette's). Given the large number of people without any language or psychiatric diagnosis, we used zero inflated Poisson regression models to determine the relationship of the HAQER CP-PGS with having any language or psychiatric diagnosis, as well as the number of language or psychiatric diagnoses. We used the log-ratio test to determine if adding the HAQER CP-PGS term significantly improved prediction accuracy in the zero inflated Poisson regression models. All other phenotypes came from the SPARK December 2014 phenotype release. All analyses included age, sex, background CP-PGS, HAQER matched control CP-PGS, and the first 5 genetic principal components as covariates.

SPARK rare ancestral reversion analysis

To explore the effects of rare genetic variation in evolutionary significant regions on language ability, we used the whole genome sequencing data from the previously described SPARK cohort (max N = 11,545) (*36*). Briefly, we merged the whole genome sequencing data provided by SPARK (WGS batches 1-4) using bcftools, then used the same processing pipeline as we did for the EpiSLI cohort: filtered to variants with a QUAL and individual quality score ≥ 20, and annotated the variants with VEP (v109) (*87*). To identify rare variants we then filtered variants in both datasets to have a maximal reference population allele frequency < 1% and an allele frequency < 1% in SPARK. Ancestral alleles were identified using those provided in the original HAQER manuscript (*34*). We identified all reversion variants within 10Kb of HAQERs from the updated set (*31*), HARs (*32*),

or random non-coding (RAND) (*34*) sequence and counted the number of rare ancestral reversions each sample had in each of these elements. We removed outlier samples who had > 2.5 median absolute deviations away from the median value for either HAQER, HAR, or random reversions, but had consistent phenotypic associations even when including these outliers in the analysis. We then used these reversion counts for association with speech and language phenotypes in SPARK.

ES-PGS replication in the UK Biobank

To further validate the effects of HAQERs on language abilities, we analyzed data from the UK Biobank cohort under Application Number #46053 (*66*). While the UK Biobank does not collect deeply phenotyped language data, they collect cognitive test scores relevant to language-related skills. For example, previous research has identified a verbal working memory task, where participants hear a sequence of digits and are asked to verbally repeat as many as they can, as highly relevant to language ability (*38*). In addition to this task, we analyzed educational attainment (a trait correlated with language abilities) and matrix reasoning, a measure of nonverbal fluid intelligence comparable to the F3 factor in our EpiSLI cohort.

We computed ES-PGS using the same pipeline established for the EpiSLI cohort, using the imputed v3 genotype data (filtered to SNPs with an imputation INFO score > 0.6 and a MAF > 1%), incorporating the first 20 genetic principal components (PCs) as covariates to mitigate the effects of population stratification. Our analysis focused on "white British European" samples; we removed all related individuals by identifying pairs with kinship score > 0.0442 using UK Biobank-provided relatedness data and randomly retaining one individual per pair, as well as removing anyone related to multiple participants. This filtering resulted in final samples of N = 84,433 unrelated individuals for the analysis of verbal working memory, N = 370,830 for educational attainment, and N = 26,051 for matrix reasoning scores. Because most large-scale cognitive GWASs include the UK Biobank in their discovery cohorts (including the cognitive performance GWAS used in our primary analyses (*28*)), for the ES-PGS we utilized the largest available GWAS on a quantitative language-related trait that excludes UK Biobank participants: a word reading ability GWAS (*9*). Although the word reading GWAS is substantially less powerful than the cognitive performance GWAS used in our primary analyses (N $\approx$ 30,000 versus N $\approx$ 250,000), it provided an independent cohort to further validate the effects of HAQERs on language-related skills. We applied rank inverse normal

transformation to all phenotypes and included year of birth, age, age^2, sex, and the first 20 genetic principal components (PCs) as covariates in the ES-PGS models.

Transcription Factor Analysis

Variant selection and annotation

We analyzed transcription factor binding sites in three distinct genomic contexts: Human Accelerated Regions (HARs), Human-Accelerated Quickly Evolved Regions (HAQERs), and matched random genomic regions (RAND, taken from (*31, 34*)). Position weight matrices (PWMs) for 633 human transcription factors were obtained from the JASPAR2020 database (*67*), with pseudocounts adjusted according to base frequency distributions.

Variants were filtered using strict quality control criteria as described above. For this analysis, we retained only biallelic single nucleotide variants (SNVs) located within feature boundaries that exhibited minor allele frequencies below 1% across all reference populations provided by VEP (*87*) and a minor allele frequency below 5% in our smaller EpiSLI sample. Complete great ape allele information was required for each variant, including data from the human reference (hg19), Neanderthals and Denisovans, Chimpanzee, Bonobo, Gorilla, and Orangutan genomes (*34, 128–131*). Briefly, archaic hominin genotype data came from VCFs with genotypes for the three high coverage Neanderthals (Altai (*128*), Chagyrskaya (*129*), and Vindija (*130*)) and one Denisovan (*131*) produced from high-coverage while genome sequencing were downloaded from the Max Planck Institute for Evolutionary Anthropology website and merged with our data. The final dataset comprised genotype information from 15,729 rare variant sites across 350 individuals, with corresponding variant annotations.

Reversion Status Determination

To characterize the evolutionary trajectory of variants, we developed a machine learning approach to impute hominin (human reference and most common allele from Neanderthal and Denisovan genomes matched) to human-chimp ancestral reversion status where direct determination was not available. We implemented an elastic net regression model (α = 0.9) using the glmnet package in R (*132*), incorporating great ape allele states as predictors of reversion status as given in (*34*). To

address class imbalance, we applied weights to ensure equal representation across sequence context types (HAQER, HAR, random). Reversion status was assigned using a probability threshold of 0.95 (i.e., at least a 95% predicted probability of being a human-chimp ancestral reversion), with known states preserved for training data.

Sequence and Motif Analysis

For each variant, we extracted 51-base pair genomic windows centered on the variant position from the human reference genome (hg19). Alternative sequences were generated by substituting variant alleles into the reference background. We then calculated maximal motif scores for both reference and alternative sequences across all JASPAR2020 human transcription factor motifs. Scores were computed on both forward and reverse complement strands, with the maximum score retained for reference and alternate alleles of each variant-motif pair.

Language Association Analysis

Sentence repetition ability, a proxy for general spoken language abilities, was assessed using the F1 measure as described above in the factor analysis. We computed burden scores for each transcription factor motif by combining variant effects weighted by genotype status of reversion sites. Linear regression models were used to estimate the associations between individual context-specific (aggregate) motif scores and language ability.

Transcription Factor Motif Score Selection Analysis

For each transcription factor, the (Z-scaled) difference in reference and alternate allele motif scores was modeled as a linear function of variant sequence context (HAQER, HAR, or random) and a binary reversion status indicator. Separate reversion effects were estimated for HAR, HAQER, and random region variants as a sequence context by reversion interaction term. Estimated beta coefficients from these terms, as well as their standard errors, were extracted for use in downstream analyses.

Joint Selection-Language Enrichment Analysis

We used York regression analysis (*133*) to examine relationships between hominin-divergent motif integrity and language-related effects. This approach accounts for uncertainty in both variables (i.e., language association betas and hominin-divergent binding effect betas). Prior to the analysis, the sign of the hominin-divergent binding binding betas were flipped such that positive values indicate human-gained motif integrity scores (i.e., when reversions to the human-chimp ancestral allele tend to decrease motif scores). York regression betas, their standard errors, a Chi-squared goodness of fit statistic and its p-value were extracted to interpret the significance of the overall relationship between TF motif integrity (i.e., motif score) and motif score effect on individual differences in language ability, for each sequence context. Individual TF motifs of interest are those with nominal significance ($p < 0.05$) for both selection for motif integrity and for positive association of aggregate motif integrity with higher sentence repetition (F1) scores. Transcription factors were classified into families using InterPro annotations (*134*) of representative families found to be significantly associated with a reversion effect in either direction (using www.string-db.org) (*135*). To identify transcription factor families showing convergent patterns of hominin selection and language association, we performed 2x2 Fisher's exact tests comparing the proportion of motifs with concordant effects (hominin gained binding AND positive language association vs. all other combinations) across families. Odds ratios with 95% confidence intervals and p-values were computed for each TF family.

Ancient DNA

DNA data and sample age information for ancient *Homo sapiens* came from the Allen Ancient DNA Resource (AADR) version 54. We downloaded the publicly available EIGENSTRAT formatted files and converted them to PLINK format using the EIGENSOFT tool. We then merged the ancient genomes with our EpiSLI and 1000 Genomes Europeans dataset to ensure we were using comparable SNPs for our ES-PGS selection analysis as we did in our discovery sample. We identified ancient west Eurasians using the same criteria as a recent large-scale selection analysis (*136*). Briefly, we filtered to samples found between longitude 25W and 60E and latitude 35N to 80N, samples passing quality control with an assessment labeled as "PASS", and sample ages > 0 but < 20,000 years old.

We then computed ES-PGS for CP in HAQERs using the same methodology as we did in the EpiSLI, SPARK, and ABCD samples for use in our polygenic selection analysis. Given the challenges of accounting for population structure in ancient DNA, we opted to use a LMM based approach instead of a traditional PC based approach as recommended by Akbari et al., 2024 (*136*). With the ancient west Eurasian subsample, we then identified independent SNPs using 1000 Genomes as the LD reference with PLINK's "–indep-pairwise" function (window size = 1000bp, step size = 1bp, r^2 threshold = 0.05, MAF ≥ 5%) (*137*). Next, we identified samples and SNPs with low missingness for GRM calculation (samples missing < 50% of independent SNPs, and SNPs missing in < 10% of those samples). Finally, we computed the genetic relatedness matrix (GRM) with GCTA (*138*) with the QC passing samples and SNPs and removed duplicate/twin samples for subsequent analysis (GCTA "grm-cutoff" of 0.9).

We then used the 3,244 QC passing samples and the GRM based on the 12,146 QC passing SNPs for the ES-PGS analysis. We implemented the LMM based polygenic selection analysis with the `gaston` (*139*) R package (`lmm.aireml` function), allowing us to account for the GRM which reflects population structure and relatedness of the sample. We used log10(sample age) as the outcome variable and HAQER CP-PGS and background CP-PGS as the independent variables (similar to the ES-PGS analysis we used in our other samples).

Additionally, we computed HAQER CP-PGS in the 10 available that are part of this release of the AADR. We compared these archaic hominins to ancient humans (described above), and 1000 Genomes Europeans, all data was processed together using the same set of SNPs to ensure polygenic scores would be comparable.

Detecting balancing selection in modern genomes

To detect signatures of balancing selection, we analyzed the WGS data we generated in EpiSLI. First, we subset to regions of interest (HAQERs, HARs, RAND, and HAQER matched control sequences). Then we computed a site frequency spectrum (SFS) for each sequence class, calculating the proportion of variants in each minor allele frequency bin. We compared HAQERs SFS to the SFS of HARs, RAND, and matched control sequences, to identify whether HAQERs had a relative enrichment of intermediate frequency variants -which can indicate balancing selection (or ongoing selection). To create 95% confidence intervals for HAQERs we generated 1,000 random bootstrap

samples of HAQERs (sampled with replacement) and computed the allele frequency proportions within in each sampled HAQER set, These confidence intervals allowed us to more confidently determine whether there was enrichment or depletion across MAF bins in HAQERs when compared to other sequence types.

Next, to more formally test our balancing selection hypothesis we identified common independent SNPs in these regions using PLINK (*137*), using the "–indep-pairwise" function (window size = 200bp, step size = 50bp, r^2 threshold = 0.5, MAF ≥ 5%) (*137*). This identified common independent SNPs in HAQERs, HARs, RAND sequences. We then computed individual level method of moments F-coefficients for each class of variation using the "–het" function in PLINK (*137*), which derives statistics based on the expected number of homozygotes and observed homozygotes (with more negative values indicating there are more heterozygotes than expected). We compared values between classes using paired t-tests to determine if there was excess heterozygosity in HAQERs, a signature of balancing selection.

ES-PGS analysis in ABCD

To explore the effects of HAQER CP-PGS in prenatal development we analyzed the ABCD cohort, a large longitudinal study of adolescent development with genetic, cognitive, brain imaging, and developmental phenotypes (*140*). All phenotypes used in ABCD came from the v4.0 data release. Similar to the SPARK replication analysis, we utilized imputed SNP array data that we have previously described (*126*) to compute ES-PGS using the same workflow as in our EpiSLI and SPARK samples. We computed genetic relatedness using GCTA (*138*) in the merged ABCD and SPARK dataset. Using the relatedness matrix, we identified unrelated individuals for ES-PGS analysis with brain imaging, cognitive, and birth phenotypes (genetic relatedness < 0.05). All evolutionary stratified polygenic scores were adjusted for genetic principal components, similar to the SPARK and EpiSLI cohorts. We included age, sex, and genetic principal components as covariates in the ES-PGS analyses.

To investigate potential evolutionary trade-offs underlying the balancing selection observed in HAQERs, we examined the relationship between HAQER CP-PGS and both cognitive performance and birth complications in ABCD. We employed a two-stage approach to address confounding and maximize signal detection. First, we applied a modified Remove Unwanted Variation using

residuals (RUVr) approach (*141*) to remove variance components in the phenotypic data that were orthogonal to the HAQER CP-PGS signal. Specifically, we identified and removed k = 15 principal components from the phenotype matrix that captured variance attributable to measured confounders (age, sex, ancestral background from genetic principal components) and unmeasured technical or biological factors not associated with HAQER CP-PGS. This denoising step preserved only phenotypic variance potentially related to our polygenic score of interest while removing confounding sources of variation. Second, we performed canonical correlation analysis (CCA) (*142*) on the RUVr-adjusted phenotypes to identify composite phenotypic dimensions that maximally correlated with HAQER CP-PGS. The CCA simultaneously analyzed two phenotypic domains (each having a block): cognitive performance measures (including memory, executive function, and processing speed tasks) and birth complication-related variables (including cesarean section delivery, intracranial volume measured from MRIs, and other obstetric risk factors). Sparsity constraints ($\lambda = 0.75$) were applied to the phenotypic loadings to improve interpretability. While we present correlation statistics for completeness, we acknowledge these may be inflated due to the optimization procedure; our primary inference focuses on the qualitative pattern of associations rather than specific effect sizes.

HAQER enrichment analysis

To determine enrichment between loci of interest (prenatal scQTLs, birth head circumference GWAS loci, and mammalian vocal learning enhancers) with our evolutionary regions of interest (HAQERs, HARs, and random sequence), we used the "intervalOverlap" function from gonomics (*143*). This allowed us to compute expected versus actual overlaps (based on size of the human genome and the provided annotations) providing an enrichment p-value for each region.

Evolutionary annotations

Given the two sets of HAQER annotations available (*31, 34*), created using different reference genomes and sequencing methodology we opted to use a conservative set of HAQERs for enrichment analyses to ensure any associations were not due to artifacts. The conservative set of HAQERs was identified by overlapping regions in the two separate HAQER sets using the bedtools "intersect" function (*123*), leaving nearly 900 overlapping HAQER regions. We included the set of HARs and

random sequence (matched to basic biological properties in HAQERs like size and non-coding sequence) from the original HAQER paper (*34*) for all enrichment analyses, allowing us to directly compare annotations. Similar to the other genomic analyses, we restricted analysis of all annotations to autosomal regions.

Defining human-specific and cell-type specific chromatin accessible regions

To identify whether HAQERs provided humans with novel regulatory mechanisms in specific neural cell-types, we examined data from snATAC-seq of human and mouse brains (*39*). To identify human-specific and cell-type specific cCREs, we used Suppelemental Table 6 provided by the authors to get hg38 coordinates of cCREs and filtered to "humanSpec" cCREs using data provided in Supplementary Table 20. Similarly, to identify human-mouse conserved cCREs, we used Suppelemental Table 6 provided by the authors and filtered to "CA_cons" cCREs using data provided in Supplementary Table 20. We added a 100Kb flank around all chromatin accessible regions and merged overlapping regions using the bedtools "merge" function, allowing us to test whether evolutionary annotations are enriched around cell-type specific chromatin accessibility regions.

Defining neurodevelopmental scQTLs

To determine if HAQERs, HARs, and RAND sequences significantly influence gene expression in prenatal and postnatal brains, we leveraged scQTLs from two studies. (1) a study of stem cell derived neurons from > 200 individuals, meant to mimic early prenatal cells in the midbrain (*49*) and (2) a study of nearly 400 postmortem adult brains from psychENCODE2 (*51*). For scQTL study (1), we defined scQTLs as all common variants (MAF > 5%) with a p-value $< 5 \times 10^{-4}$, for scQTL study (2) we utilized all provided scQTLs defined as significant by the authors downloaded from `https://psychscreen.wenglab.org/psychscreen/downloads`.

Defining birth head circumference regions

We identified birth head circumference associated genomic loci with a genome-wide suggestive p-value (p-value $< 5 \times 10^{-5}$) and a MAF > 5%, using the largest GWAS available to date (*52*).

Given the influence of LD on human traits, we added a 100Kb flank around all associated SNPs and merged overlapping regions using the bedtools "merge" function, allowing us to test whether evolutionary annotations are enriched around associated SNPs.

Defining mammalian vocal learning enhancer regions

We gathered previously established mammalian vocal learning enhancer regions from a recent cross-species analysis (*15*), which identified 50 open chromatin regions in the motor cortex strongly associated with vocal learning. Since these regions were provided in mouse genome coordinates (mm10), we lifted these over to hg19 and we added 1Mb flanks around these enhancer regions which were then used to test whether evolutionary annotations are enriched near established vocal learning enhancers.

Convergent evolution of vocal learning analysis

To test for evidence of convergent evolution of "HAQER-like" sequences in vocal learning species, we utilized a large dataset of > 400 species with whole genome data aligned to the human reference genome (hg38) (*55*). We parsed the alignments with Biopython (*144*) to subset to regions of interest. For each species and sequence type (HAQERs or HARs), we computed a sequence similarity (using the number of bases matching the human reference genome in these regions). We then used this "HAQER-like" sequence similarity to predict vocal learning status in 170 non-primate species that have been previously described (*15*). To determine statistical significance and account for species relatedness we used phylogenetic logistic regression (*56*) with the `phylolm` package (*57*) in R. We did similar analyses for brain size and birth:adult weight ratio using species level phenotype data from the PanTHERIA dataset (*68*).

Statistical analysis

All statistical analysis was done in R (version 4.3.1) (*77*).

Approach to multiple testing correction and triangulating evidence

To address multiple testing concerns, we employed False Discovery Rate (FDR) correction for analyses testing more than 20 hypotheses, allowing us to limit type 1 errors (false positives). This was applied to the EpiSLI CBCL mental health score analysis, genome-wide PGS analysis, and the ES-PGS analysis where we conducted many statistical tests.

We adopted a comprehensive approach to validate our key findings through multiple lines of evidence. We established confidence in our results through: (1) replication across independent cohorts (e.g., EpiSLI, SPARK, ABCD, and the UK Biobank); (2) identifying convergent evidence across different analytical approaches (e.g., common variant, rare variant, and transcription factor binding analyses); (3) demonstration of specificity, showing HAQERs' associations with language but not nonverbal IQ; (4) evolutionary support from ancient DNA and cross-species analyses; and (5) mechanistic support through analysis of transcription factor binding, enrichment for human-specific and cell-type specific chromatin accessible regions, and enrichment of variants influencing prenatal gene regulation. This multi-faceted approach allowed us to distinguish robust biological signals from statistical noise, even when some individual analyses showed moderate statistical significance. Our findings' consistency across diverse data types, species, and analytical methods provides stronger evidence than would be achieved through any single statistical test, regardless of its p-value.

Supplementary Figures

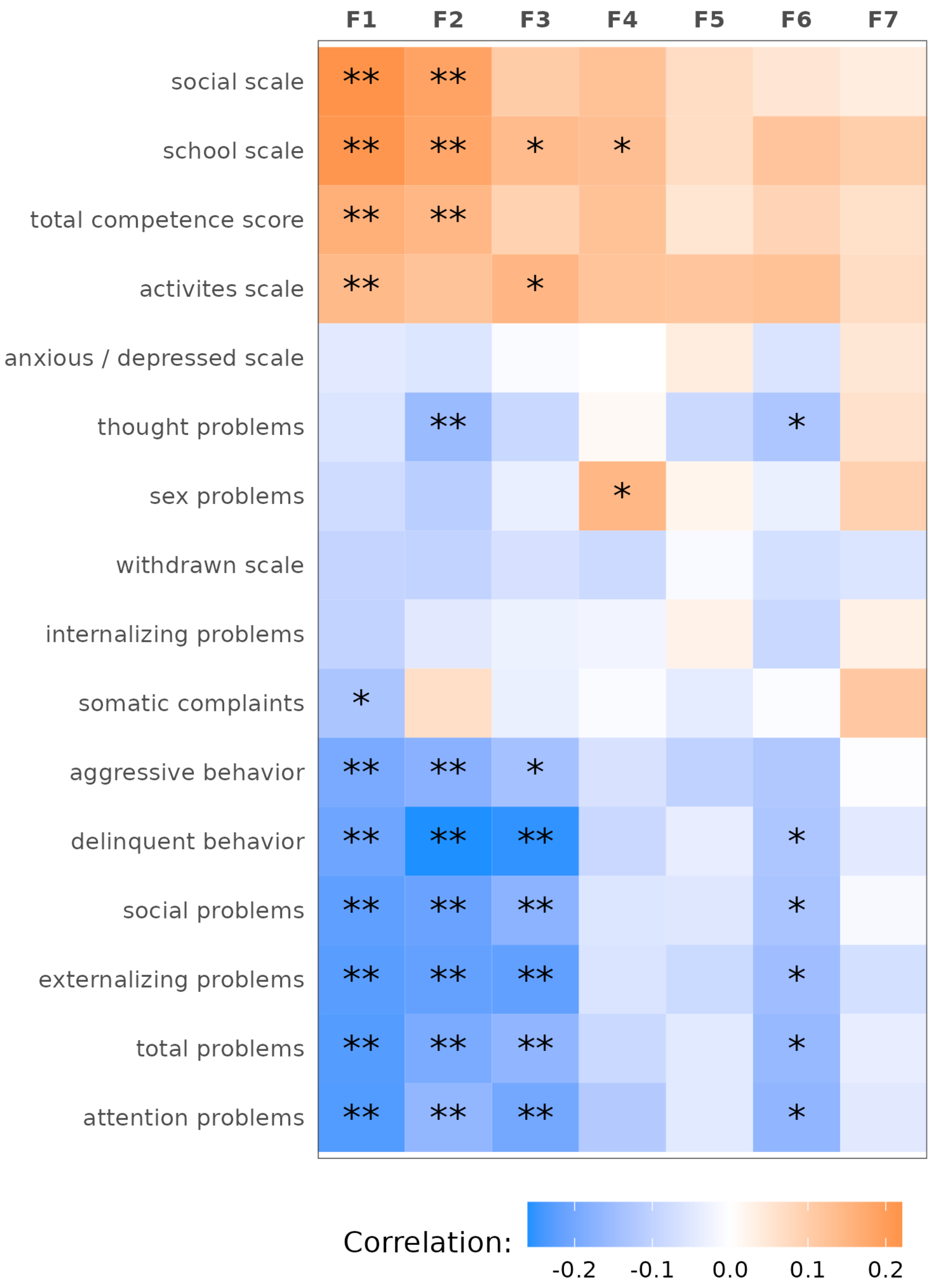

Figure S1: **Mental health correlates of EpiSLI factors**

Heatmap of Pearson correlation coefficients (*r*) of CBCL scales with EpiSLI factor scores (N = 241 individuals). Color indicates the association between that CBCL subscale score and the EpiSLI factor score (orange = positive correlation, blue = negative correlation. "**" indicates FDR < 0.05, and "*" indicates unadjusted p-value < 0.05.

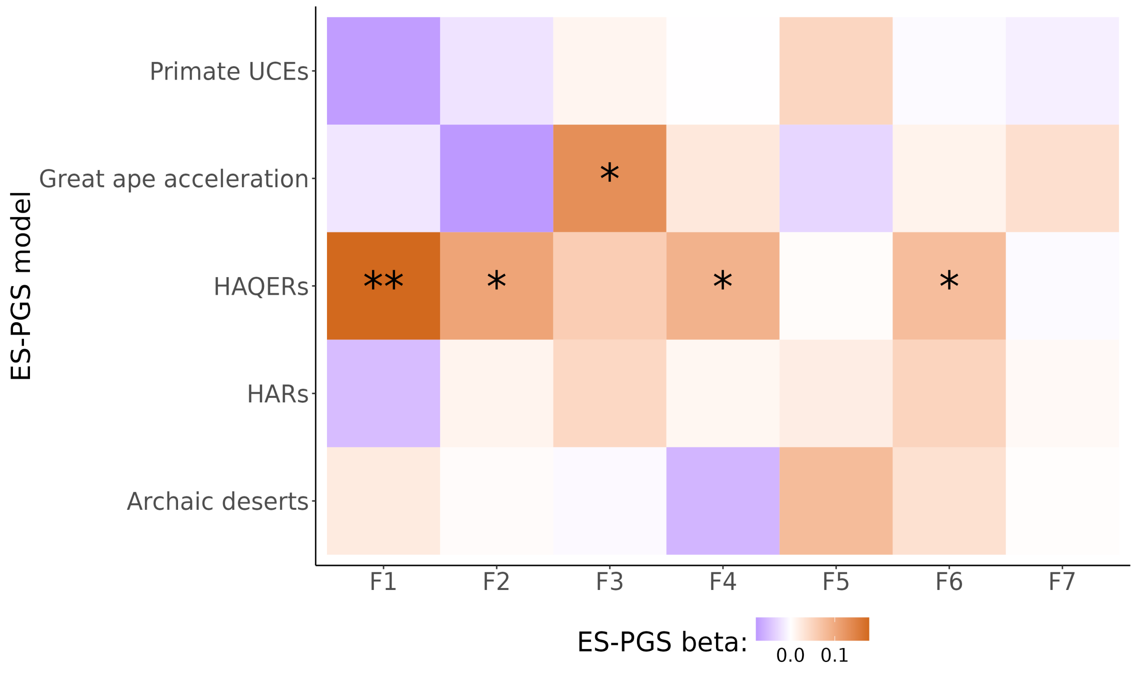

Figure S2: **Evolutionary history of EpiSLI factors**

Heatmap of ES-PGS β's stratifying the CP-PGS based on evolutionary annotations. Color indicates the association between that evolutionary annotation and the EpiSLI factor score (orange = positive ES-PGS β, purple = negative ES-PGS β). "**" indicates FDR < 0.01, and "*" indicates unadjusted p-value < 0.05.

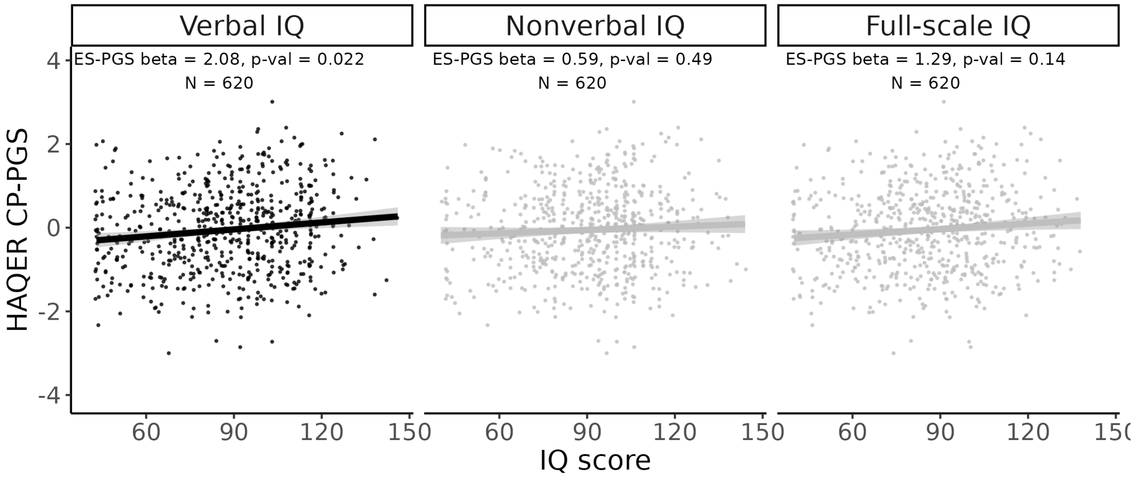

Figure S3:

HAQER CP-PGS is associated with verbal IQ in an independent cohort

ES-PGS results of HAQER CP-PGS and clinical IQ assessment scores from the SPARK dataset (N = 620).

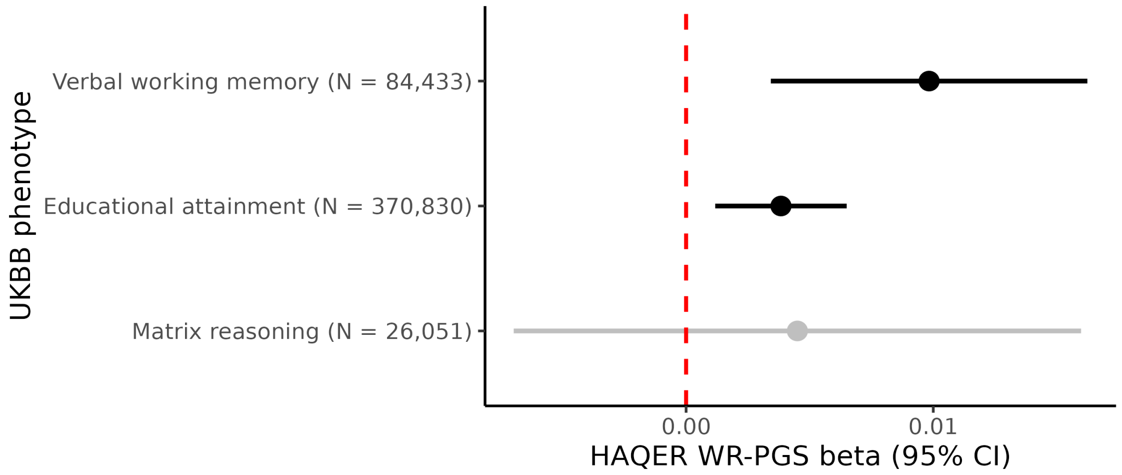

Figure S4:

HAQER WR-PGS is associated with verbal working memory in an independent cohort

ES-PGS results of the HAQER word reading polygenic score (WR-PGS) and relevant cognition-related traits in the UK Biobank.

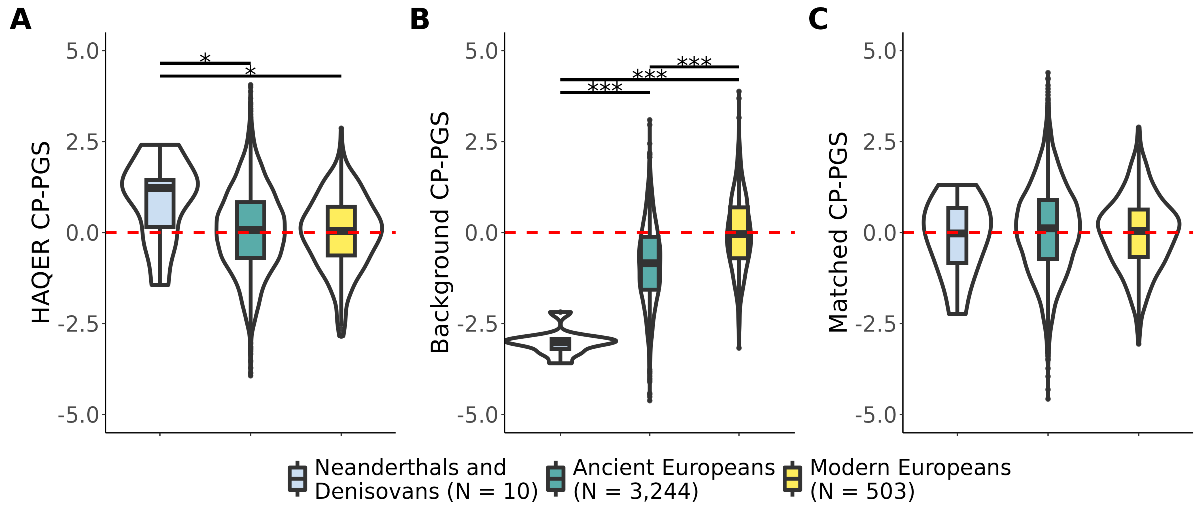

Figure S5:

ES-PGS differences across human history

A-C Distribution of HAQER CP-PGS (**A**), background CP-PGS (**B**), or random matched control regions (**C**) across archaic humans (neanderthals and denisovans from the AADR), ancient anatomically modern humans (AADR), and modern Europeans (1000 Genomes dataset). "*" indicates a p-value of < 0.05 from a t-test comparison, and "***" indicates p-value < 0.001.

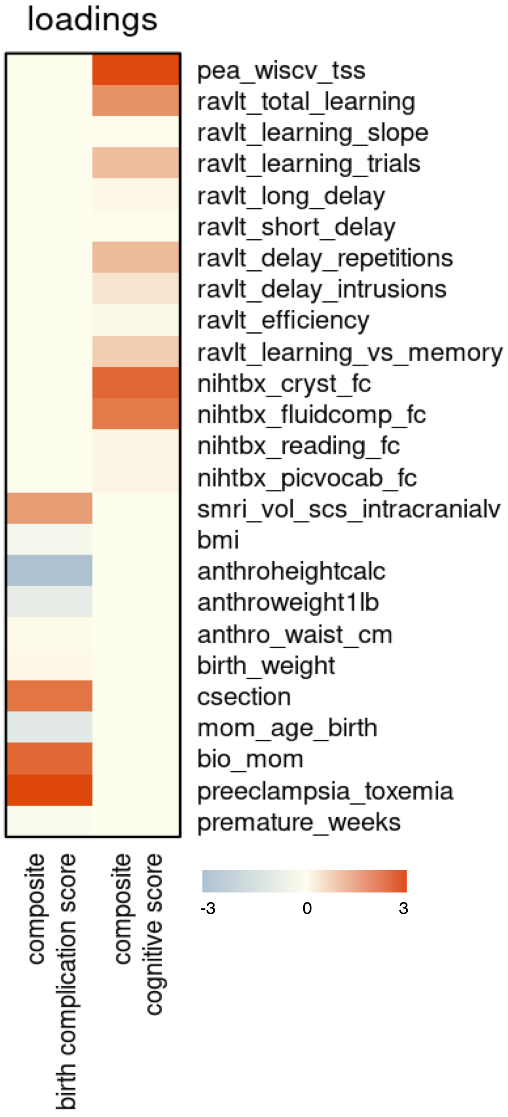

Figure S6: **Phenotypic dimensions of cognition and birth complications**

Composite phenotypic scores derived from ABCD cohort data using regularized canonical correlation analysis with HAQER CP-PGS. This method identifies linear combinations of variables maximally correlated with HAQER CP-PGS while enforcing sparsity (parameter = 0.75). Block 2: 14 cognitive measures (RAVLT, NIH Toolbox cognition, reading, vocabulary, matrix reasoning). Block 3: 11 birth/development measures (intracranial volume, anthropometrics, birth weight, delivery mode, maternal factors, prematurity). Heatmap shows sparse canonical loadings; Component 1 = composite birth complication score, Component 2 = composite cognitive score. Orange = positive loadings onto component, while blue = negative loading.

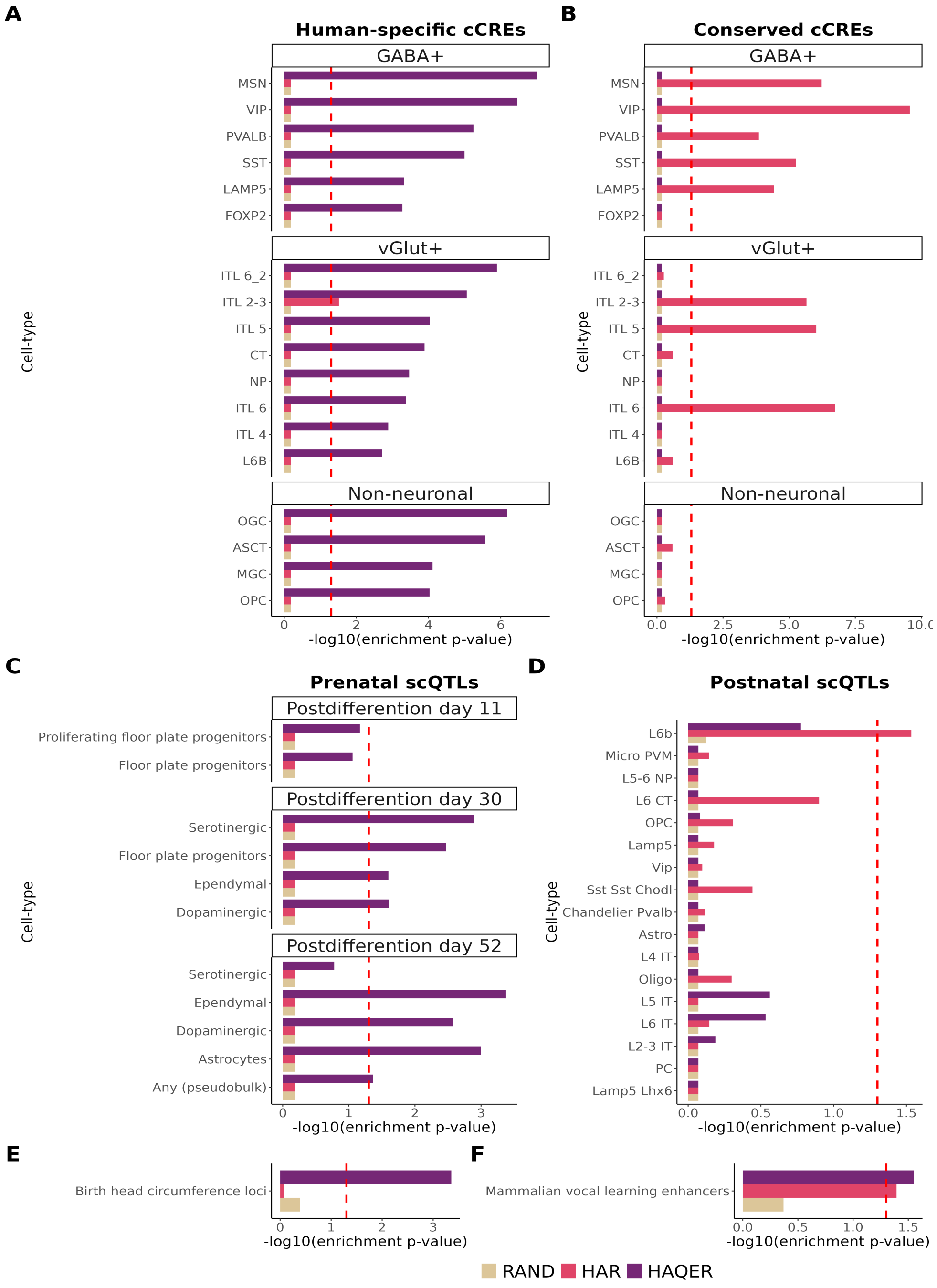

Figure S7: **HAQERs influence prenatal brain development**

A-B Enrichment of HAQERs, HARs, and RAND for brain cCREs from human-specific (**A**) or human-mouse conserved (**B**) elements. **C-D** Enrichment for regulatory variants (scQTLs) across timepoints and cell-types in newly differentiated neurons (**C**) or adult post mortem brains (**D**). **E** Enrichment for common SNPs (MAF > 5%) associated with head circumference at birth. **F** Enrichment for enhancer regions associated with vocal learning across mammals (Wirthlin et al., 2024).

Supplementary Tables

Table S1. < in a separate .xlsx file >

Correlation results from EpiSLI cohort factor scores with CBCL scales. Correlation summary statistics for all EpiSLI factors with Child Behavior Checklist (CBCL) subscales, as seen in Figure S1.

Table S2. < in a separate .xlsx file >

Correlation results from EpiSLI cohort factor scores with genome wide polygenic scores. Correlation summary statistics for all EpiSLI factors with polygenic scores calculated using LDpred2, as seen in Figure 2D.

Table S3. < in a separate .xlsx file >

ES-PGS results from EpiSLI cohort factor scores. ES-PGS summary statistics for all EpiSLI factors, as seen in Figure 3 and Figure S2.

Table S4. < in a separate .xlsx file >

Validation of HAQER CP-PGS in larger SPARK cohort. HAQER CP-PGS summary statistics for SPARK validation analyses, as seen in Figure 4A and Figure S3.

Table S5. < in a separate .xlsx file >

Validation of HAQER CP-PGS in SPARK research match cohort. HAQER CP-PGS summary statistics for SPARK validation analyses in the Research Match cohort, as seen in Figure 4B.

Table S6. < in a separate .xlsx file >

SPARK WGS rare reversion analysis results. Summary statistics from the rare ancestral reversion analysis of HAQERs, HARs, and RAND sequences in the SPARK whole genome sequencing dataset, as seen in Figure 4C-F.

Table S7. < in a separate .xlsx file >

ABCD HAQER CP-PGS validation analysis and birth related traits. HAQER CP-PGS summary statistics for ABCD validation analyses.

Table S8. < in a separate .xlsx file >

Polygenic selection results from ancient human data from the AADR cohort. Summary statistics for cognitive performance polygenic score selection over recent human evolutionary history in the AADR dataset, including the HAQER CP-PGS, seen in Figure 6A.

Table S9. < in a separate .xlsx file >

ES-PGS comparison results of AADR groups to 1,000 Genomes Europeans. Summary statistics comparing the HAQER partitions of the cognitive performance polygenic score between archaic humans from the AADR (N = 10), ancient humans from the AADR (N = 3,244), and modern Europeans from 1000 Genomes (N = 503), as seen in Figure S5.

Table S10. < in a separate .xlsx file >

Cross-species HAQER-like similarity and phenotypic data. Dataset for cross-species genomic comparisons of non-primate mammals for HAQER-like and HAR-like sequence similarity (compared to the hg38 human reference genome). This dataset was used in Figure 6 E-G.

Table S11. < in a separate .xlsx file >

Phylogenetic regression results from the cross-species analysis. Summary statistics from phylogenetic regression analyses of HAQER-like and HAR-like sequence similarity in non-primate mammals of vocal learning, brain size, and a birth:adult weight ratio phenotype.

Table S12. < in a separate .xlsx file >

Enrichment of pre/postnatal scQTLs in HAQERs. Summary statistics from overlap enrichment analysis of HAQERs, HARs, and RAND sequences with single-cell QTLs of developing and adult neurons. Results shown in Figure S7 C-D.

Table S13. < in a separate .xlsx file >

Enrichment of birth head circumference GWAS loci in HAQERs. Summary statistics from overlap enrichment analysis of HAQERs, HARs, and RAND sequences with suggestive GWAS loci for head circumference at birth. Results shown in Figure S7 E.

Table S14. < in a separate .xlsx file > **Enrichment of mammalian vocal learning enhancer regions in HAQERs.**

Summary statistics from overlap enrichment analysis of HAQERs, HARs, and RAND sequences with previously identified mammalian vocal learning enhancer genomic regions. Results shown in Figure S7 F.

Table S15. < in a separate .xlsx file >

Results from hominin-gained TFBS analysis in relation to EpiSLI F1. Summary statistics from analysis of predicted hominin gained TFBS across HAQERs, HARs, and RAND sequences and TFBS score correlations with F1 scores from the EpiSLI cohort. Results shown in Figure 5 A-D.

Table S16. < in a separate .xlsx file >

Enrichment results of TF families with both hominin-gained TF motif integrity and positive motif integrity-language associations. Summary statistics from analysis of enrichment of TF families for predicted hominin gained TFBS that show positive correlations with F1 scores in EpiSLI. Results shown in Figure 5 E.

Table S17. < in a separate .xlsx file >

EpiSLI whole genome sequencing coverage statistics. Whole genome sequencing coverage statistics for the newly generated EpiSLI genomic dataset.

Table S18. < in a separate .xlsx file >

Enrichment of human-specific and conserved cCREs in HAQERs. Summary statistics from overlap enrichment analysis of HAQERs, HARs, and RAND sequences with brain single-cell chromatin accessible regions specific to humans (”Human-specific”) or evolutionarily conserved between humans and mice (”Conserved”). Results shown in Figure S7 A-B.

REFERENCES

1. M. V. Suntsova, A. A. Buzdin, Differences between human and chimpanzee genomes and their implications in gene expression, protein functions and biochemical properties of the two species. *BMC Genomics* **21**, 535 (2020).

2. R. J. Britten, Divergence between samples of chimpanzee and human DNA sequences is 5%, counting indels. *Proc. Natl. Acad. Sci. U.S.A.* **99**, 13633–13635 (2002).

3. A. Varki, T. K. Altheide, Comparing the human and chimpanzee genomes: Searching for needles in a haystack. *Genome Res.* **15**, 1746–1758 (2005).

4. S. E. Fisher, Evolution of language: Lessons from the genome. *Psychon. Bull. Rev.* **24**, 34–40 (2017).

5. C. S. L. Lai, S. E. Fisher, J. A. Hurst, F. Vargha-Khadem, A. P. Monaco, A forkhead-domain gene is mutated in a severe speech and language disorder. *Nature* **413**, 519–523 (2001).

6. S. E. Fisher, C. Scharff, FOXP2 as a molecular window into speech and language. *Trends Genet.* **25**, 166–177 (2009).

7. E. G. Atkinson, A. J. Audesse, J. A. Palacios, D. M. Bobo, A. E. Webb, S. Ramachandran, B. M. Henn, No evidence for recent selection at FOXP2 among diverse human populations. *Cell* **174**, 1424–1435.e15 (2018).

8. K. L. Mueller, J. C. Murray, J. J. Michaelson, M. H. Christiansen, S. Reilly, J. B. Tomblin, Common genetic variants in FOXP2 are not associated with individual differences in language development. *PLOS ONE* **11**, e0152576 (2016).

9. E. Eising, N. Mirza-Schreiber, E. L. de Zeeuw, C. A. Wang, D. T. Truong, A. G. Allegrini, C. Y. Shapland, G. Zhu, K. G. Wigg, M. L. Gerritse, B. Molz, G. Alagöz, A. Gialluisi, F. Abbondanza, K. Rimfeld, M. van Donkelaar, Z. Liao, P. R. Jansen, T. F. M. Andlauer, T. C. Bates, M. Bernard, K. Blokland, M. Bonte, A. D. Børglum, T. Bourgeron, D. Brandeis, F. Ceroni, V. Csépe, P. S. Dale, P. F. de Jong, J. C. DeFries, J.-F. Démonet, D. Demontis, Y. Feng, S. D. Gordon, S. L. Guger, M. E. Hayiou-Thomas, J. A. Hernández-Cabrera, J.-J. Hottenga, C.

Hulme, J. Kere, E. N. Kerr, T. Koomar, K. Landerl, G. T. Leonard, M. W. Lovett, H. Lyytinen, N. G. Martin, A. Martinelli, U. Maurer, J. J. Michaelson, K. Moll, A. P. Monaco, A. T. Morgan, M. M. Nöthen, Z. Pausova, C. E. Pennell, B. F. Pennington, K. M. Price, V. M. Rajagopal, F. Ramus, L. Richer, N. H. Simpson, S. D. Smith, M. J. Snowling, J. Stein, L. J. Strug, J. B. Talcott, H. Tiemeier, M. P. van der Schroeff, E. Verhoef, K. E. Watkins, M. Wilkinson, M. J. Wright, C. L. Barr, D. I. Boomsma, M. Carreiras, M. C. J. Franken, J. R. Gruen, M. Luciano, B. Müller-Myhsok, D. F. Newbury, R. K. Olson, S. Paracchini, T. Paus, R. Plomin, S. Reilly, G. Schulte-Körne, J. B. Tomblin, E. van Bergen, A. J. O. Whitehouse, E. G. Willcutt, B. St Pourcain, C. Francks, S. E. Fisher, Genome-wide analyses of individual differences in quantitatively assessed reading- and language-related skills in up to 34,000 people. *Proc. Natl. Acad. Sci. U.S.A.* **119**, e2202764119 (2022).

10. H. G. Polikowsky, A. C. Scartozzi, D. M. Shaw, D. G. Pruett, H.-H. Chen, L. E. Petty, A. S. Petty, E. J. Lowther, S.-H. Cho, Y. Yu, 23andMe Research Team, S. Aslibekyan, A. Auton, E. Babalola, R. K. Bell, J. Bielenberg, K. Bryc, E. Bullis, D. Coker, G. C. Partida, D. Dhamija, S. das, S. L. Elson, T. Filshtein, K. Fletez-Brant, P. Fontanillas, W. Freyman, P. M. Gandhi, K. Heilbron, B. Hicks, D. A. Hinds, E. M. Jewett, Y. Jiang, K. Kukar, K. H. Lin, M. Lowe, J. McCreight, M. H. McIntyre, S. J. Micheletti, M. E. Moreno, J. L. Mountain, P. Nandakumar, E. S. Noblin, J. O'Connell, A. A. Petrakovitz, G. D. Poznik, M. Schumacher, A. J. Shastri, J. F. Shelton, J. Shi, S. Shringarpure, V. Tran, J. Y. Tung, X. Wang, W. Wang, C. H. Weldon, P. Wilton, A. Hernandez, C. Wong, C. T. Tchakouté, S. Mozaffari, C. L. Avery, K. M. Harris, R. L. Gordon, J. M. Beilby, K. Z. Viljoen, R. M. Jones, C. D. Huff, H. M. Highland, S. J. Kraft, J. E. Below, Large-scale genome-wide analyses of stuttering. *Nat. Genet.* **57**, 1835–1847 (2025).

11. E. Verhoef, A. G. Allegrini, P. R. Jansen, K. Lange, C. A. Wang, A. T. Morgan, T. S. Ahluwalia, C. Symeonides, O. A. Andreassen, M. Bartels, D. Boomsma, P. S. Dale, E. Ehli, D. Fernandez-Orth, M. Guxens, C. Hakulinen, K. M. Harris, S. Haworth, L. de Hoyos, V. Jaddoe, L. Keltikangas-Järvinen, T. Lehtimäki, C. Middeldorp, J. L. Min, P. P. Mishra, P. R. Njølstad, J. Sunyer, A. E. Tate, N. Timpson, C. van der Laan, M. Vrijheid, E. Vuoksimaa, A. Whipp, E. Ystrom, ACTION Consortium, Barwon Infant Study investigator group, E. Eising, M. C. Franken, E. Hypponen, T. Mansell, M. Olislagers, E. Omerovic, K. Rimfeld, F. Schlag, S. Selzam, C. Y. Shapland, H. Tiemeier, A. J. O. Whitehouse, R. Saffery, K. Bønnelykke, S.

Reilly, C. E. Pennell, M. Wake, C. A. M. Cecil, R. Plomin, S. E. Fisher, B. St. Pourcain, Genome-wide analyses of vocabulary size in infancy and toddlerhood: Associations with attention-deficit/hyperactivity disorder, literacy, and cognition-related traits. *Biol. Psychiatry* **95**, 859–869 (2024).

12. G. Alagöz, E. Eising, Y. Mekki, G. Bignardi, P. Fontanillas, 23andMe Research Team, S. Aslibekyan, A. Auton, E. Babalola, R. K. Bell, J. Bielenberg, J. Bowes, K. Bryc, N. S. Chaudhary, D. Coker, S. das, E. DelloRusso, S. L. Elson, N. Eriksson, T. Filshtein, W. Freyman, Z. Fuller, C. German, J. M. Granka, K. Heilbron, A. Hernandez, B. Hicks, D. A. Hinds, E. M. Jewett, Y. Jiang, K. Kukar, A. Kwong, Y. Liang, K. H. Lin, B. A. Llamas, M. H. McIntyre, S. J. Micheletti, M. E. Moreno, P. Nandakumar, D. T. Nguyen, J. O'Connell, A. A. Petrakovitz, G. D. Poznik, A. Reynoso, S. Saini, M. Schumacher, L. Selcer, A. J. Shastri, J. F. Shelton, J. Shi, S. Shringarpure, Q. J. Su, S. A. Tat, V. Tran, J. Y. Tung, X. Wang, W. Wang, C. H. Weldon, P. Wilton, C. D. Wong, M. G. Nivard, M. Luciano, N. J. Cox, S. E. Fisher, R. L. Gordon, The shared genetic architecture and evolution of human language and musical rhythm. *Nat. Hum. Behav.* **9**, 376–390 (2025).

13. C. Doust, P. Fontanillas, E. Eising, S. D. Gordon, Z. Wang, G. Alagöz, B. Molz, 23andMe Research Team, S. Aslibekyan, A. Auton, E. Babalola, R. K. Bell, J. Bielenberg, K. Bryc, E. Bullis, D. Coker, G. C. Partida, D. Dhamija, S. das, S. L. Elson, T. Filshtein, K. Fletez-Brant, W. Freyman, P. M. Gandhi, K. Heilbron, B. Hicks, D. A. Hinds, E. M. Jewett, Y. Jiang, K. Kukar, K. H. Lin, M. Lowe, J. McCreight, M. H. McIntyre, S. J. Micheletti, M. E. Moreno, J. L. Mountain, P. Nandakumar, E. S. Noblin, J. O'Connell, A. A. Petrakovitz, G. D. Poznik, M. Schumacher, A. J. Shastri, J. F. Shelton, J. Shi, S. Shringarpure, V. Tran, J. Y. Tung, X. Wang, W. Wang, C. H. Weldon, P. Wilton, A. Hernandez, C. Wong, C. T. Tchakouté, Quantitative Trait Working Group of the GenLang Consortium, F. Abbondanza, A. G. Allegrini, T. F. M. Andlauer, C. L. Barr, M. Bernard, K. Blokland, M. Bonte, D. I. Boomsma, T. Bourgeron, D. Brandeis, M. Carreiras, F. Ceroni, V. Csépe, P. S. Dale, P. F. de Jong, J. F. Démonet, E. L. de Zeeuw, Y. Feng, M. C. J. Franken, M. Gerritse, A. Gialluisi, S. L. Guger, M. E. Hayiou-Thomas, J. Hernández-Cabrera, J. J. Hottenga, C. Hulme, P. R. Jansen, J. Kere, E. N. Kerr, T. Koomar, K. Landerl, G. T. Leonard, Z. Liao, M. W. Lovett, H. Lyytinen, A. Martinelli, U. Maurer, J. J. Michaelson, N. Mirza-Schreiber, K. Moll, A. T. Morgan, B. Müller-Myhsok, D. F. Newbury, M. M. Nöthen, T. Paus, Z. Pausova, C. E. Pennell, R. J. Plomin, K. M. Price, F.

Ramus, S. Reilly, L. Richer, K. Rimfeld, G. Schulte-Körne, C. Y. Shapland, N. H. Simpson, M. J. Snowling, J. F. Stein, L. J. Strug, H. Tiemeier, J. B. Tomblin, D. T. Truong, E. van Bergen, M. P. van der Schroeff, M. van Donkelaar, E. Verhoef, C. A. Wang, K. E. Watkins, A. J. O. Whitehouse, K. G. Wigg, M. Wilkinson, G. Zhu, B. S. Pourcain, C. Francks, R. E. Marioni, J. Zhao, S. Paracchini, J. B. Talcott, A. P. Monaco, J. F. Stein, J. R. Gruen, R. K. Olson, E. G. Willcutt, J. C. DeFries, B. F. Pennington, S. D. Smith, M. J. Wright, N. G. Martin, A. Auton, T. C. Bates, S. E. Fisher, M. Luciano, Discovery of 42 genome-wide significant loci associated with dyslexia. *Nat. Genet.* **54**, 1621–1629 (2022).

14. M. Niarchou, D. E. Gustavson, J. F. Sathirapongsasuti, M. Anglada-Tort, E. Eising, E. Bell, E. McArthur, P. Straub, 23andMe Research Team, S. Aslibekyan, A. Auton, R. K. Bell, K. Bryc, S. K. Clark, S. L. Elson, K. Fletez-Brant, P. Fontanillas, N. A. Furlotte, P. M. Gandhi, K. Heilbron, B. Hicks, K. E. Huber, E. M. Jewett, Y. Jiang, A. Kleinman, K. H. Lin, N. K. Litterman, J. C. McCreight, M. H. McIntyre, K. F. McManus, J. L. Mountain, S. V. Mozaffari, P. Nandakumar, E. S. Noblin, C. A. M. Northover, J. O'Connell, S. J. Pitts, G. D. Poznik, A. J. Shastri, J. F. Shelton, S. Shringarpure, C. Tian, J. Y. Tung, R. J. Tunney, V. Vacic, X. Wang, J. D. McAuley, J. A. Capra, F. Ullén, N. Creanza, M. A. Mosing, D. A. Hinds, L. K. Davis, N. Jacoby, R. L. Gordon, Genome-wide association study of musical beat synchronization demonstrates high polygenicity. *Nat. Hum. Behav.* **6**, 1292–1309 (2022).

15. M. E. Wirthlin, T. A. Schmid, J. E. Elie, X. Zhang, A. Kowalczyk, R. Redlich, V. A. Shvareva, A. Rakuljic, M. B. Ji, N. S. Bhat, I. M. Kaplow, D. E. Schäffer, A. J. Lawler, A. Z. Wang, B. Doi N Phan, S. Annaldasula, A. R. Brown, T. Lu, B. K. Lim, E. Azim, Z. Consortium, N. L. Clark, W. K. Meyer, S. L. Kosakovsky Pond, M. Chikina, M. M. Yartsev, A. R. Pfenning, G. Andrews, J. C. Armstrong, M. Bianchi, B. W. Birren, K. R. Bredemeyer, A. M. Breit, M. J. Christmas, H. Clawson, J. Damas, F. D. Palma, M. Diekhans, M. X. Dong, E. Eizirik, K. Fan, C. Fanter, N. M. Foley, K. Forsberg-Nilsson, C. J. Garcia, J. Gatesy, S. Gazal, D. P. Genereux, L. Goodman, J. Grimshaw, M. K. Halsey, A. J. Harris, G. Hickey, M. Hiller, A. G. Hindle, R. M. Hubley, G. M. Hughes, J. Johnson, D. Juan, I. M. Kaplow, E. K. Karlsson, K. C. Keough, B. Kirilenko, K.-P. Koepfli, J. M. Korstian, A. Kowalczyk, S. V. Kozyrev, A. J. Lawler, C. Lawless, T. Lehmann, D. L. Levesque, H. A. Lewin, X. Li, A. Lind, K. Lindblad-Toh, A. Mackay-Smith, V. D. Marinescu, T. Marques-Bonet, V. C. Mason, J. R. S. Meadows, W. K. Meyer, J. E. Moore, L. R. Moreira, D. D. Moreno-Santillan, K. M.

Morrill, G. Muntané, W. J. Murphy, A. Navarro, M. Nweeia, S. Ortmann, A. Osmanski, B. Paten, N. S. Paulat, A. R. Pfenning, B. Doi N Phan, K. S. Pollard, H. E. Pratt, D. A. Ray, S. K. Reilly, J. R. Rosen, I. Ruf, L. Ryan, O. A. Ryder, P. C. Sabeti, D. E. Schäffer, A. Serres, B. Shapiro, A. F. A. Smit, M. Springer, C. Srinivasan, C. Steiner, J. M. Storer, K. A. M. Sullivan, P. F. Sullivan, E. Sundström, M. A. Supple, R. Swofford, J.-E. Talbot, E. Teeling, J. Turner-Maier, A. Valenzuela, F. Wagner, O. Wallerman, C. Wang, J. Wang, Z. Weng, A. P. Wilder, M. E. Wirthlin, J. R. Xue, X. Zhang, Vocal learning-associated convergent evolution in mammalian proteins and regulatory elements. *Science* **383**, eabn3263 (2024).

16. I. M. Kaplow, A. J. Lawler, D. E. Schäffer, C. Srinivasan, H. H. Sestili, M. E. Wirthlin, B. D. N. Phan, K. Prasad, A. R. Brown, X. Zhang, K. Foley, D. P. Genereux, Zoonomia Consortium**, E. K. Karlsson, K. Lindblad-Toh, W. K. Meyer, A. R. Pfenning, G. Andrews, J. C. Armstrong, M. Bianchi, B. W. Birren, K. R. Bredemeyer, A. M. Breit, M. J. Christmas, H. Clawson, J. Damas, F. di Palma, M. Diekhans, M. X. Dong, E. Eizirik, K. Fan, C. Fanter, N. M. Foley, K. Forsberg-Nilsson, C. J. Garcia, J. Gatesy, S. Gazal, D. P. Genereux, L. Goodman, J. Grimshaw, M. K. Halsey, A. J. Harris, G. Hickey, M. Hiller, A. G. Hindle, R. M. Hubley, G. M. Hughes, J. Johnson, D. Juan, I. M. Kaplow, E. K. Karlsson, K. C. Keough, B. Kirilenko, K. P. Koepfli, J. M. Korstian, A. Kowalczyk, S. V. Kozyrev, A. J. Lawler, C. Lawless, T. Lehmann, D. L. Levesque, H. A. Lewin, X. Li, A. Lind, K. Lindblad-Toh, A. Mackay-Smith, V. D. Marinescu, T. Marques-Bonet, V. C. Mason, J. R. S. Meadows, W. K. Meyer, J. E. Moore, L. R. Moreira, D. D. Moreno-Santillan, K. M. Morrill, G. Muntané, W. J. Murphy, A. Navarro, M. Nweeia, S. Ortmann, A. Osmanski, B. Paten, N. S. Paulat, A. R. Pfenning, B. D. N. Phan, K. S. Pollard, H. E. Pratt, D. A. Ray, S. K. Reilly, J. R. Rosen, I. Ruf, L. Ryan, O. A. Ryder, P. C. Sabeti, D. E. Schäffer, A. Serres, B. Shapiro, A. F. A. Smit, M. Springer, C. Srinivasan, C. Steiner, J. M. Storer, K. A. M. Sullivan, P. F. Sullivan, E. Sundström, M. A. Supple, R. Swofford, J. E. Talbot, E. Teeling, J. Turner-Maier, A. Valenzuela, F. Wagner, O. Wallerman, C. Wang, J. Wang, Z. Weng, A. P. Wilder, M. E. Wirthlin, J. R. Xue, X. Zhang, Relating enhancer genetic variation across mammals to complex phenotypes using machine learning. *Science* **380**, eabm7993 (2023).

17. E. D. Jarvis, Evolution of vocal learning and spoken language. *Science* **366**, 50–54 (2019).

18. R. L. Gordon, A. Ravignani, J. Hyland Bruno, C. M. Robinson, A. Scartozzi, R. Embalabala, M. Niarchou, 23andMe Research Team, N. J. Cox, N. Creanza, Linking the genomic signatures of human beat synchronization and learned song in birds. *Philos. Trans. R. Soc. Lond. B Biol. Sci.* **376**, 20200329 (2021).

19. J. A. Cahill, J. Armstrong, A. Deran, C. J. Khoury, B. Paten, D. Haussler, E. D. Jarvis, Positive selection in noncoding genomic regions of vocal learning birds is associated with genes implicated in vocal learning and speech functions in humans. *Genome Res.* **31**, 2035–2049 (2021).

20. M. Sebastianelli, S. M. Lukhele, S. Secomandi, S. G. de Souza, B. Haase, M. Moysi, C. Nikiforou, A. Hutfluss, J. Mountcastle, J. Balacco, S. Pelan, W. Chow, O. Fedrigo, C. T. Downs, A. Monadjem, N. J. Dingemanse, E. D. Jarvis, A. Brelsford, B. M. vonHoldt, A. N. G. Kirschel, A genomic basis of vocal rhythm in birds. *Nat. Commun.* **15**, 3095 (2024).

21. J. B. Tomblin, The EpiSLI database: A publicly available database on speech and language. *Lang. Speech Hear. Serv. Sch.* **41**, 108–117 (2010).

22. M. Klem, M. Melby-Lervåg, B. Hagtvet, S. A. H. Lyster, J. E. Gustafsson, C. Hulme, Sentence repetition is a measure of children's language skills rather than working memory limitations. *Dev. Sci.* **18**, 146–154 (2015).

23. I. Rujas, S. Mariscal, E. Murillo, M. Lázaro, Sentence repetition tasks to detect and prevent language difficulties: A scoping review. *Children* **8**, 578 (2021).

24. S. H. Lee, T. R. De Candia, S. Ripke, J. Yang, Schizophrenia Psychiatric Genome-Wide Association Study Consortium (PGC-SCZ), International Schizophrenia Consortium (ISC), Molecular Genetics of Schizophrenia Collaboration (MGS), P. F. Sullivan, M. E. Goddard, M. C. Keller, P. M. Visscher, N. R. Wray, Estimating the proportion of variation in susceptibility to schizophrenia captured by common SNPs. *Nat. Genet.* **44**, 247–250 (2012).

25. H. K. Finucane, B. Bulik-Sullivan, A. Gusev, G. Trynka, Y. Reshef, P.-R. Loh, V. Anttila, H. Xu, C. Zang, K. Farh, S. Ripke, F. R. Day, ReproGen Consortium, Schizophrenia Working Group of the Psychiatric Genomics Consortium, RACI Consortium, S. Purcell, E. Stahl, S.

Lindstrom, J. R. B. Perry, Y. Okada, S. Raychaudhuri, M. J. Daly, N. Patterson, B. M. Neale, A. L. Price, Partitioning heritability by functional annotation using genome-wide association summary statistics. *Nat. Genet.* **47**, 1228–1235 (2015).

26. X. Wei, C. R. Robles, A. Pazokitoroudi, A. Ganna, A. Gusev, A. Durvasula, S. Gazal, P. R. Loh, D. Reich, S. Sankararaman, The lingering effects of Neanderthal introgression on human complex traits. *eLife* **12**, e80757 (2023).

27. S. W. Choi, J. García-González, Y. Ruan, H. M. Wu, C. Porras, J. Johnson, Bipolar Disorder Working group of the Psychiatric Genomics Consortium, C. J. Hoggart, P. F. O'Reilly, PRSet: Pathway-based polygenic risk score analyses and software. *PLOS Genet.* **19**, e1010624 (2023).

28. J. J. Lee, R. Wedow, A. Okbay, E. Kong, O. Maghzian, M. Zacher, T. A. Nguyen-Viet, P. Bowers, J. Sidorenko, R. K. Linnér, M. A. Fontana, T. Kundu, C. Lee, H. Li, R. Li, R. Royer, P. N. Timshel, R. K. Walters, E. A. Willoughby, L. Yengo, 23andMe Research Team, COGENT (Cognitive Genomics Consortium), Social Science Genetic Association Consortium, M. Alver, Y. Bao, D. W. Clark, F. R. Day, N. A. Furlotte, P. K. Joshi, K. E. Kemper, A. Kleinman, C. Langenberg, R. Mägi, J. W. Trampush, S. S. Verma, Y. Wu, M. Lam, J. H. Zhao, Z. Zheng, J. D. Boardman, H. Campbell, J. Freese, K. M. Harris, C. Hayward, P. Herd, M. Kumari, T. Lencz, J. Luan, A. K. Malhotra, A. Metspalu, L. Milani, K. K. Ong, J. R. B. Perry, D. J. Porteous, M. D. Ritchie, M. C. Smart, B. H. Smith, J. Y. Tung, N. J. Wareham, J. F. Wilson, J. P. Beauchamp, D. C. Conley, T. Esko, S. F. Lehrer, P. K. E. Magnusson, S. Oskarsson, T. H. Pers, M. R. Robinson, K. Thom, C. Watson, C. F. Chabris, M. N. Meyer, D. I. Laibson, J. Yang, M. Johannesson, P. D. Koellinger, P. Turley, P. M. Visscher, D. J. Benjamin, D. Cesarini, Gene discovery and polygenic prediction from a genome-wide association study of educational attainment in 1.1 million individuals. *Nat. Genet.* **50**, 1112–1121 (2018).

29. L. F. K. Kuderna, J. C. Ulirsch, S. Rashid, M. Ameen, L. Sundaram, G. Hickey, A. J. Cox, H. Gao, A. Kumar, F. Aguet, M. J. Christmas, H. Clawson, M. Haeussler, M. C. Janiak, M. Kuhlwilm, J. D. Orkin, T. Bataillon, S. Manu, A. Valenzuela, J. Bergman, M. Rouselle, F. E. Silva, L. Agueda, J. Blanc, M. Gut, D. de Vries, I. Goodhead, R. A. Harris, M. Raveendran,

A. Jensen, I. S. Chuma, J. E. Horvath, C. Hvilsom, D. Juan, P. Frandsen, J. G. Schraiber, F. R. de Melo, F. Bertuol, H. Byrne, I. Sampaio, I. Farias, J. Valsecchi, M. Messias, M. N. F. da Silva, M. Trivedi, R. Rossi, T. Hrbek, N. Andriaholinirina, C. J. Rabarivola, A. Zaramody, C. J. Jolly, J. Phillips-Conroy, G. Wilkerson, C. Abee, J. H. Simmons, E. Fernandez-Duque, S. Kanthaswamy, F. Shiferaw, D. Wu, L. Zhou, Y. Shao, G. Zhang, J. D. Keyyu, S. Knauf, M. D. le, E. Lizano, S. Merker, A. Navarro, T. Nadler, C. C. Khor, J. Lee, P. Tan, W. K. Lim, A. C. Kitchener, D. Zinner, I. Gut, A. D. Melin, K. Guschanski, M. H. Schierup, R. M. D. Beck, I. Karakikes, K. C. Wang, G. Umapathy, C. Roos, J. P. Boubli, A. Siepel, A. Kundaje, B. Paten, K. Lindblad-Toh, J. Rogers, T. Marques Bonet, K. K. H. Farh, Identification of constrained sequence elements across 239 primate genomes. *Nature* **625**, 735–742 (2024).

30. X. Bi, L. Zhou, J. J. Zhang, S. Feng, M. Hu, D. N. Cooper, J. Lin, J. Li, D. D. Wu, G. Zhang, Lineage-specific accelerated sequences underlying primate evolution. *Sci. Adv.* **9**, eadc9507 (2023).

31. D. Yoo, A. Rhie, P. Hebbar, F. Antonacci, G. A. Logsdon, S. J. Solar, D. Antipov, B. D. Pickett, Y. Safonova, F. Montinaro, Y. Luo, J. Malukiewicz, J. M. Storer, J. Lin, A. N. Sequeira, R. J. Mangan, G. Hickey, G. Monfort Anez, P. Balachandran, A. Bankevich, C. R. Beck, A. Biddanda, M. Borchers, G. G. Bouffard, E. Brannan, S. Y. Brooks, L. Carbone, L. Carrel, A. P. Chan, J. Crawford, M. Diekhans, E. Engelbrecht, C. Feschotte, G. Formenti, G. H. Garcia, L. de Gennaro, D. Gilbert, R. E. Green, A. Guarracino, I. Gupta, D. Haddad, J. Han, R. S. Harris, G. A. Hartley, W. T. Harvey, M. Hiller, K. Hoekzema, M. L. Houck, H. Jeong, K. Kamali, M. Kellis, B. Kille, C. Lee, Y. Lee, W. Lees, A. P. Lewis, Q. Li, M. Loftus, Y. H. E. Loh, H. Loucks, J. Ma, Y. Mao, J. F. I. Martinez, P. Masterson, R. C. McCoy, B. McGrath, S. McKinney, B. S. Meyer, K. H. Miga, S. K. Mohanty, K. M. Munson, K. Pal, M. Pennell, P. A. Pevzner, D. Porubsky, T. Potapova, F. R. Ringeling, J. L. Rocha, O. A. Ryder, S. Sacco, S. Saha, T. Sasaki, M. C. Schatz, N. J. Schork, C. Shanks, L. Smeds, D. R. Son, C. Steiner, A. P. Sweeten, M. G. Tassia, F. Thibaud-Nissen, E. Torres-González, M. Trivedi, W. Wei, J. Wertz, M. Yang, P. Zhang, S. Zhang, Y. Zhang, Z. Zhang, S. A. Zhao, Y. Zhu, E. D. Jarvis, J. L. Gerton, I. Rivas-González, B. Paten, Z. A. Szpiech, C. D. Huber, T. L. Lenz, M. K. Konkel, S. V. Yi, S. Canzar, C. T. Watson, P. H. Sudmant, E. Molloy, E. Garrison, C. B. Lowe, M. Ventura, R. J. O'Neill, S. Koren, K. D. Makova, A. M. Phillippy, E. E. Eichler, Complete sequencing of ape genomes. *Nature* **641**, 401–418 (2025).

32. J. A. Capra, G. D. Erwin, G. McKinsey, J. L. R. Rubenstein, K. S. Pollard, Many human accelerated regions are developmental enhancers. *Philos. Trans. R. Soc. Lond. B Biol. Sci.* **368**, 20130025 (2013).

33. S. Peyrégne, M. J. Boyle, M. Dannemann, K. Prüfer, Detecting ancient positive selection in humans using extended lineage sorting. *Genome Res.* **27**, 1563–1572 (2017).

34. R. J. Mangan, F. C. Alsina, F. Mosti, J. E. Sotelo-Fonseca, D. A. Snellings, E. H. Au, J. Carvalho, L. Sathyan, G. D. Johnson, T. E. Reddy, D. L. Silver, C. B. Lowe, Adaptive sequence divergence forged new neurodevelopmental enhancers in humans. *Cell* **185**, 4587–4603.e23 (2022).

35. K. S. Pollard, S. R. Salama, B. King, A. D. Kern, T. Dreszer, S. Katzman, A. Siepel, J. S. Pedersen, G. Bejerano, R. Baertsch, K. R. Rosenbloom, J. Kent, D. Haussler, Forces shaping the fastest evolving regions in the human genome. *PLOS Genet.* **2**, e168 (2006).

36. P. Feliciano, A. M. Daniels, L. A. Green Snyder, A. Beaumont, A. Camba, A. Esler, A. G. Gulsrud, A. Mason, A. Gutierrez, A. Nicholson, A. M. Paolicelli, A. P. McKenzie, A. L. Rachubinski, A. N. Stephens, A. R. Simon, A. Stedman, A. D. Shocklee, A. Swanson, B. Finucane, B. A. Hilscher, B. Hauf, B. J. O'Roak, B. McKenna, B. E. Robertson, B. Rodriguez, B. M. Vernoia, B. van Metre, C. Bradley, C. Cohen, C. A. Erickson, C. Harkins, C. Hayes, C. Lord, C. L. Martin, C. Ortiz, C. Ochoa-Lubinoff, C. Peura, C. E. Rice, C. R. Rosenberg, C. J. Smith, C. Thomas, C. M. Taylor, L. C. White, C. H. Walston, D. G. Amaral, D. L. Coury, D. E. Sarver, D. Istephanous, D. Li, D. C. Nugyen, E. A. Fox, E. M. Butter, E. Berry-Kravis, E. Courchesne, E. J. Fombonne, E. Hofammann, E. Lamarche, E. L. Wodka, E. T. Matthews, E. O'Connor, E. Palen, F. Miller, G. S. Dichter, G. Marzano, G. Stein, H. Hutter, H. E. Kaplan, H. Li, H. Lechniak, H. L. Schneider, H. Zaydens, I. Arriaga, J. A. Gerdts, J. F. Cubells, J. M. Cordova, J. Gunderson, J. Lillard, J. Manoharan, J. T. McCracken, J. J. Michaelson, J. Neely, J. Orobio, J. Pandey, J. Piven, J. Scherr, J. S. Sutcliffe, J. Tjernagel, J. Wallace, K. Callahan, K. Dent, K. A. Schweers, K. E. Hamer, J. K. Law, K. Lowe, K. O'Brien, K. Smith, K. G. Pawlowski, K. L. Pierce, K. Roeder, L. J. Abbeduto, L. N. Berry, L. A. Cartner, L. A. Coppola, L. Carpenter, L. Cordeiro, L. DeMarco, L. P. Grosvenor, L. Higgins, L. Y. Huang-Storms, L. Hosmer-Quint, L. M. Herbert, L. Kasparson,

L. M. Prock, L. D. Pacheco, L. Raymond, L. Simon, L. V. Soorya, L. Wasserburg, M. Lazar, M. Alessandri, M. Brown, M. H. Currin, M. L. F. Gwynette, M. Heyman, M. N. Hale, M. Jones, M. Jordy, M. J. Morrier, M. Sahin, M. S. Siegel, M. Verdi, M. V. Parlade, M. Yinger, N. Bardett, N. Hanna, N. Harris, N. Pottschmidt, N. Russo-Ponsaran, N. Takahashi, O. Y. Ousley, A. P. Juarez, P. Manning, R. D. Annett, R. A. Bernier, R. D. Clark, R. J. Landa, R. P. Goin-Kochel, R. Remington, R. T. Schultz, S. J. Brewster, S. Booker, S. Carpenter, S. Eldred, S. Francis, S. L. Friedman, S. Horner, S. Hepburn, S. Jacob, S. Kanne, S. J. Lee, S. A. Mastel, S. Plate, S. Qiu, S. Sandhu, S. Thompson, S. White, V. J. Myers, V. Singh, W. S. Yang, Z. Warren, A. Amatya, A. J. Ace, A. S. Chatha, A. E. Lash, B. Negron, C. Rigby, C. Ridenour, C. M. Stock, D. Schmidt, I. Fisk, J. Acampado, J. L. Nestle, J. A. Nestle, K. Layman, M. E. Butler, M. Kent, M. D. Mallardi, N. Carriero, N. Lawson, N. Volfovsky, R. Edgar, R. Marini, R. Rana, S. Ganesan, S. Shah, T. Ramsey, W. Chin, W. Jensen, A. D. Krentz, A. J. Gruber, A. Sabo, A. Salomatov, C. Eng, D. Muzny, I. Astrovskaya, R. A. Gibbs, X. Han, Y. Shen, L. F. Reichardt, W. K. Chung, SPARK: A US cohort of 50,000 families to accelerate autism research. *Neuron* **97**, 488–493 (2018).

37. K. M. Lisdahl, K. J. Sher, K. P. Conway, R. Gonzalez, S. W. Feldstein Ewing, S. J. Nixon, S. Tapert, H. Bartsch, R. Z. Goldstein, M. Heitzeg, Adolescent brain cognitive development (ABCD) study: Overview of substance use assessment methods. *Dev. Cogn. Neurosci.* **32**, 80–96 (2018).

38. G. A. Jonsdottir, G. Einarsson, G. Thorleifsson, S. H. Magnusson, A. F. Gunnarsson, M. L. Frigge, R. S. Gisladottir, U. Unnsteinsdottir, B. Gunnarsson, G. B. Walters, V. Steinthorsdottir, A. Helgadottir, I. Jonsdottir, T. Gislason, H. S. Thorsteinsson, E. Sigurdsson, M. Haraldsson, E. L. Sigurdsson, R. Bjarnason, I. Olafsson, G. Thorgeirsson, P. Sulem, H. Holm, U. Thorsteinsdottir, D. F. Gudbjartsson, G. Bjornsdottir, T. E. Thorgeirsson, H. Stefansson, K. Stefansson, Genetic propensities for verbal and spatial ability have opposite effects on body mass index and risk of schizophrenia. *Intelligence* **88**, 101565 (2021).

39. Y. E. Li, S. Preissl, M. Miller, N. D. Johnson, Z. Wang, H. Jiao, C. Zhu, Z. Wang, Y. Xie, O. Poirion, C. Kern, A. Pinto-Duarte, W. Tian, K. Siletti, N. Emerson, J. Osteen, J. Lucero, L. Lin, Q. Yang, Q. Zhu, N. Zemke, S. Espinoza, A. M. Yanny, J. Nyhus, N. Dee, T. Casper, N. Shapovalova, D. Hirschstein, R. D. Hodge, S. Linnarsson, T. Bakken, B. Levi, C. D. Keene,

J. Shang, E. Lein, A. Wang, M. M. Behrens, J. R. Ecker, B. Ren, A comparative atlas of single-cell chromatin accessibility in the human brain. *Science* **382**, eadf7044 (2023).

40. L. Xiao, D. P. Merullo, T. M. I. Koch, M. Cao, M. Co, A. Kulkarni, G. Konopka, T. F. Roberts, Expression of FoxP2 in the basal ganglia regulates vocal motor sequences in the adult songbird. *Nat. Commun.* **12**, 2617 (2021).

41. M. T. Ullman, G. M. Clark, M. Y. Pullman, J. T. Lovelett, E. I. Pierpont, X. Jiang, P. E. Turkeltaub, The neuroanatomy of developmental language disorder: A systematic review and meta-analysis. *Nat. Hum. Behav.* **8**, 962–975 (2024).

42. S. Mallick, A. Micco, M. Mah, H. Ringbauer, I. Lazaridis, I. Olalde, N. Patterson, D. Reich, The Allen Ancient DNA Resource (AADR) a curated compendium of ancient human genomes. *Sci. Data* **11**, 182 (2024).

43. Y. Ding, K. Hou, Z. Xu, A. Pimplaskar, E. Petter, K. Boulier, F. Privé, B. J. Vilhjálmsson, L. M. Olde Loohuis, B. Pasaniuc, Polygenic scoring accuracy varies across the genetic ancestry continuum. *Nature* **618**, 774–781 (2023).

44. D. Dediu, S. C. Levinson, Neanderthal language revisited: Not only us. *Curr. Opin. Behav. Sci.* **21**, 49–55 (2018).

45. M. Conde-Valverde, I. Martínez, R. M. Quam, M. Rosa, A. D. Velez, C. Lorenzo, P. Jarabo, J. M. Bermúdez de Castro, E. Carbonell, J. L. Arsuaga, Neanderthals and *Homo sapiens* had similar auditory and speech capacities. *Nat. Ecol. Evol.* **5**, 609–615 (2021).

46. J. Krause, C. Lalueza-Fox, L. Orlando, W. Enard, R. E. Green, H. A. Burbano, J. J. Hublin, C. Hänni, J. Fortea, M. de la Rasilla, J. Bertranpetit, A. Rosas, S. Pääbo, The derived FOXP2 variant of modern humans was shared with Neandertals. *Curr. Biol.* **17**, 1908–1912 (2007).

47. V. Llaurens, A. Whibley, M. Joron, Genetic architecture and balancing selection: The life and death of differentiated variants. *Mol. Ecol.* **26**, 2430–2448 (2017).

48. B. D. Bitarello, D. Y. C. Brandt, D. Meyer, A. M. Andrés, Inferring balancing selection from genome-scale data. *Genome Biol. Evol.* **15**, evad032 (2023).

49. J. Jerber, D. D. Seaton, A. S. E. Cuomo, N. Kumasaka, J. Haldane, J. Steer, M. Patel, D. Pearce, M. Andersson, M. J. Bonder, E. Mountjoy, M. Ghoussaini, M. A. Lancaster, HipSci Consortium, J. C. Marioni, F. T. Merkle, D. J. Gaffney, O. Stegle, Population-scale single-cell RNA-seq profiling across dopaminergic neuron differentiation. *Nat. Genet.* **53**, 304–312 (2021).

50. R. A. I. Bethlehem, J. Seidlitz, S. R. White, J. W. Vogel, K. M. Anderson, C. Adamson, S. Adler, G. S. Alexopoulos, E. Anagnostou, A. Areces-Gonzalez, D. E. Astle, B. Auyeung, M. Ayub, J. Bae, G. Ball, S. Baron-Cohen, R. Beare, S. A. Bedford, V. Benegal, F. Beyer, J. Blangero, M. Blesa Cábez, J. P. Boardman, M. Borzage, J. F. Bosch-Bayard, N. Bourke, V. D. Calhoun, M. M. Chakravarty, C. Chen, C. Chertavian, G. Chetelat, Y. S. Chong, J. H. Cole, A. Corvin, M. Costantino, E. Courchesne, F. Crivello, V. L. Cropley, J. Crosbie, N. Crossley, M. Delarue, R. Delorme, S. Desrivieres, G. A. Devenyi, M. A. di Biase, R. Dolan, K. A. Donald, G. Donohoe, K. Dunlop, A. D. Edwards, J. T. Elison, C. T. Ellis, J. A. Elman, L. Eyler, D. A. Fair, E. Feczko, P. C. Fletcher, P. Fonagy, C. E. Franz, L. Galan-Garcia, A. Gholipour, J. Giedd, J. H. Gilmore, D. C. Glahn, I. M. Goodyer, P. E. Grant, N. A. Groenewold, F. M. Gunning, R. E. Gur, R. C. Gur, C. F. Hammill, O. Hansson, T. Hedden, A. Heinz, R. N. Henson, K. Heuer, J. Hoare, B. Holla, A. J. Holmes, R. Holt, H. Huang, K. Im, J. Ipser, C. R. Jack Jr., A. P. Jackowski, T. Jia, K. A. Johnson, P. B. Jones, D. T. Jones, R. S. Kahn, H. Karlsson, L. Karlsson, R. Kawashima, E. A. Kelley, S. Kern, K. W. Kim, M. G. Kitzbichler, W. S. Kremen, F. Lalonde, B. Landeau, S. Lee, J. Lerch, J. D. Lewis, J. Li, W. Liao, C. Liston, M. V. Lombardo, J. Lv, C. Lynch, T. T. Mallard, M. Marcelis, R. D. Markello, S. R. Mathias, B. Mazoyer, P. McGuire, M. J. Meaney, A. Mechelli, N. Medic, B. Misic, S. E. Morgan, D. Mothersill, J. Nigg, M. Q. W. Ong, C. Ortinau, R. Ossenkoppele, M. Ouyang, L. Palaniyappan, L. Paly, P. M. Pan, C. Pantelis, M. M. Park, T. Paus, Z. Pausova, D. Paz-Linares, A. Pichet Binette, K. Pierce, X. Qian, J. Qiu, A. Qiu, A. Raznahan, T. Rittman, A. Rodrigue, C. K. Rollins, R. Romero-Garcia, L. Ronan, M. D. Rosenberg, D. H. Rowitch, G. A. Salum, T. D. Satterthwaite, H. L. Schaare, R. J. Schachar, A. P. Schultz, G. Schumann, M. Schöll, D. Sharp, R. T. Shinohara, I. Skoog, C. D. Smyser, R. A. Sperling, D. J. Stein, A. Stolicyn, J. Suckling, G. Sullivan, Y. Taki, B. Thyreau, R. Toro, N. Traut, K. A. Tsvetanov, N. B. Turk-Browne, J. J. Tuulari, C. Tzourio, É. Vachon-Presseau, M. J. Valdes-Sosa, P. A. Valdes-Sosa, S. L. Valk, T. van Amelsvoort, S. N. Vandekar, L. Vasung, L. W. Victoria, S. Villeneuve, A. Villringer, P. E. Vértes, K. Wagstyl, Y. S. Wang, S. K. Warfield, V.

Warrier, E. Westman, M. L. Westwater, H. C. Whalley, A. V. Witte, N. Yang, B. Yeo, H. Yun, A. Zalesky, H. J. Zar, A. Zettergren, J. H. Zhou, H. Ziauddeen, A. Zugman, X. N. Zuo, 3R-BRAIN, AIBL, C. Rowe, Alzheimer's Disease Neuroimaging Initiative, Alzheimer's Disease Repository Without Borders Investigators, G. B. Frisoni, CALM Team, Cam-CAN, CCNP, COBRE, cVEDA, ENIGMA Developmental Brain Age Working Group, Developing Human Connectome Project, FinnBrain, Harvard Aging Brain Study, IMAGEN, KNE96, The Mayo Clinic Study of Aging, NSPN, POND, The PREVENT-AD Research Group, A. P. Binette, VETSA, E. T. Bullmore, A. F. Alexander-Bloch, Brain charts for the human lifespan. *Nature* **604**, 525–533 (2022).

51. P. S. Emani, J. J. Liu, D. Clarke, M. Jensen, J. Warrell, C. Gupta, R. Meng, C. Y. Lee, S. Xu, C. Dursun, S. Lou, Y. Chen, Z. Chu, T. Galeev, A. Hwang, Y. Li, P. Ni, X. Zhou, PsychENCODE Consortium, T. E. Bakken, J. Bendl, L. Bicks, T. Chatterjee, L. Cheng, Y. Cheng, Y. Dai, Z. Duan, M. Flaherty, J. F. Fullard, M. Gancz, D. Garrido-Martín, S. Gaynor-Gillett, J. Grundman, N. Hawken, E. Henry, G. E. Hoffman, A. Huang, Y. Jiang, T. Jin, N. L. Jorstad, R. Kawaguchi, S. Khullar, J. Liu, J. Liu, S. Liu, S. Ma, M. Margolis, S. Mazariegos, J. Moore, J. R. Moran, E. Nguyen, N. Phalke, M. Pjanic, H. Pratt, D. Quintero, A. S. Rajagopalan, T. R. Riesenmy, N. Shedd, M. Shi, M. Spector, R. Terwilliger, K. J. Travaglini, B. Wamsley, G. Wang, Y. Xia, S. Xiao, A. C. Yang, S. Zheng, M. J. Gandal, D. Lee, E. S. Lein, P. Roussos, N. Sestan, Z. Weng, K. P. White, H. Won, M. J. Girgenti, J. Zhang, D. Wang, D. Geschwind, M. Gerstein, S. Akbarian, A. Abyzov, N. Ahituv, D. Arasappan, J. J. Almagro Armenteros, B. J. Beliveau, S. Berretta, R. A. Bharadwaj, A. Bhattacharya, K. Brennand, D. Capauto, F. A. Champagne, C. Chatzinakos, H. I. Chen, L. Cheng, A. Chess, J. F. Chien, A. Clement, L. Collado-Torres, G. M. Cooper, G. E. Crawford, R. Dai, N. P. Daskalakis, J. Davila-Velderrain, A. Deep-Soboslay, C. Deng, C. P. DiPietro, S. Dracheva, S. Drusinsky, D. Duong, N. J. Eagles, J. Edelstein, K. Galani, K. Girdhar, F. S. Goes, W. Greenleaf, H. Guo, Q. Guo, Y. Hadas, J. Hallmayer, X. Han, V. Haroutunian, C. He, S. C. Hicks, M. Ho, L. L. Ho, Y. Huang, L. A. Huuki-Myers, T. M. Hyde, A. Iatrou, F. Inoue, A. Jajoo, L. Jiang, P. Jin, C. Jops, A. Jourdon, M. Kellis, J. E. Kleinman, S. P. Kleopoulos, A. Kozlenkov, A. Kriegstein, A. Kundaje, S. Kundu, J. Li, M. Li, X. Lin, S. Liu, C. Liu, J. M. Loupe, D. Lu, L. Ma, J. Mariani, K. Martinowich, K. R. Maynard, R. M. Myers, C. Micallef, T. Mikhailova, G. L. Ming, S. Mohammadi, E. Monte, K. S. Montgomery, E. A. Mukamel, A. C. Nairn, C. B. Nemeroff, S. Norton, T. Nowakowski, L. Omberg, S. C. Page, S. Park, A.

Patowary, R. Pattni, G. Pertea, M. A. Peters, D. Pinto, S. Pochareddy, K. S. Pollard, A. Pollen, P. F. Przytycki, C. Purmann, Z. S. Qin, P. P. Qu, T. Raj, S. Reach, T. Reimonn, K. J. Ressler, D. Ross, J. Rozowsky, M. Ruth, W. B. Ruzicka, S. J. Sanders, J. M. Schneider, S. Scuderi, R. Sebra, N. Seyfried, Z. Shao, A. W. Shieh, J. H. Shin, M. Skarica, C. Snijders, H. Song, M. W. State, J. Stein, M. Steyert, S. Subburaju, T. Sudhof, M. Snyder, R. Tao, K. Therrien, L. H. Tsai, A. E. Urban, F. M. Vaccarino, H. van Bakel, D. Vo, G. Voloudakis, T. Wang, S. H. Wang, Y. Wang, Y. Wei, A. K. Weimer, D. R. Weinberger, C. Wen, S. Whalen, A. J. Willsey, W. Wong, H. Wu, F. Wu, S. Wuchty, D. Wylie, C. X. Yap, B. Zeng, P. Zhang, C. Zhang, B. Zhang, Y. Zhang, R. Ziffra, Z. R. Zeier, T. M. Zintel, Single-cell genomics and regulatory networks for 388 human brains. *Science* **384**, eadi5199 (2024).

52. S. Vogelezang, J. P. Bradfield, the Early Growth Genetics Consortium, S. Vogelezang, J. P. Bradfield, S. Johansson, E. Stergiakouli, E. Thiering, C. E. Pennell, T. S. Ahluwalia, V. Karhunen, M. Scholz, X. Liu, C. Iñiguez, O. T. Raitakari, J. Bacelis, T. M. Schnurr, T. A. Lakka, I. Ntalla, M. V. Lind, L. Lauritzen, S. Herrala, F. T. J. Lin, C. Frithioff-Bøjsøe, R. N. Beaumont, M. H. Zafarmand, P. Rzehak, J. R. Bilbao, N. Junna, J. M. Vonk, S. L. Rifas-Shiman, V. D. Janjanam, M. J. Knol, S. Ramdas, L. Beilin, K. Bønnelykke, M. Casas, J. G. Eriksson, J. Escribano, T. E. Evans, U. Gehring, F. Geller, V. Grote, D. Gruszfeld, H. Hakonarson, A. T. Hattersley, C. T. Have, M. Geoffrey Hayes, J. Heinrich, Ø. Helgeland, J. Holloway, R. Joro, J. Juodakis, B. A. Knight, B. Koletzko, A. Körner, J. P. Langhendries, J. T. Leinonen, V. Lindi, M. A. V. Lund, S. Lye, M. Melbye, K. F. Michaelsen, C. S. Morgen, H. Niinikoski, K. Pahkala, K. Panoutsopoulou, O. Pedersen, F. Rivadeneira, L. Santa-Marina, B. D. C. Schaik, D. Scholtens, S. Sebert, I. Tamayo, N. J. Timpson, M. Torrent, A. G. Uitterlinden, M. Vaudel, E. Verduci, R. Vinding, M. Vogel, E. Zeggini, C. Brown, H. H. H. Adams, W. Karmaus, M. F. Hivert, G. H. Koppelman, E. Widén, N. Fernandez-Jimenez, M. Waldenberger, T. G. M. Vrijkotte, R. M. Freathy, J. C. Holm, W. L. Lowe Jr., N. Grarup, T. Hansen, G. V. Dedoussis, M. Atalay, E. A. Nohr, B. Jacobsson, N. Pitkänen, M. Vrijheid, B. Feenstra, W. Kiess, M. R. Jarvelin, H. Bisgaard, C. Wang C, M. Standl, M. I. McCarthy, B. S. Pourcain, P. R. Njølstad, S. F. A. Grant, J. F. Felix, V. W. V. Jaddoe, Genetics of early-life head circumference and genetic correlations with neurological, psychiatric and cognitive outcomes. *BMC Med. Genomics* **15**, 124 (2022).

53. N. D. S. Grunstra, L. Betti, B. Fischer, M. Haeusler, M. Pavlicev, E. Stansfield, W. Trevathan, N. M. Webb, J. C. K. Wells, K. R. Rosenberg, P. Mitteroecker, There is an obstetrical dilemma: Misconceptions about the evolution of human childbirth and pelvic form. *Am. J. Biol. Anthropol.* **181**, 535–544 (2023).

54. L. Xu, E. Kun, D. Pandey, J. Y. Wang, M. F. Brasil, T. Singh, V. M. Narasimhan, The genetic architecture of and evolutionary constraints on the human pelvic form. *Science* **388**, eadq1521 (2025).

55. L. F. K. Kuderna, H. Gao, M. C. Janiak, M. Kuhlwilm, J. D. Orkin, T. Bataillon, S. Manu, A. Valenzuela, J. Bergman, M. Rousselle, F. E. Silva, L. Agueda, J. Blanc, M. Gut, D. de Vries, I. Goodhead, R. A. Harris, M. Raveendran, A. Jensen, I. S. Chuma, J. E. Horvath, C. Hvilsom, D. Juan, P. Frandsen, J. G. Schraiber, F. R. de Melo, F. Bertuol, H. Byrne, I. Sampaio, I. Farias, J. Valsecchi, M. Messias, M. N. F. da Silva, M. Trivedi, R. Rossi, T. Hrbek, N. Andriaholinirina, C. J. Rabarivola, A. Zaramody, C. J. Jolly, J. Phillips-Conroy, G. Wilkerson, C. Abee, J. H. Simmons, E. Fernandez-Duque, S. Kanthaswamy, F. Shiferaw, D. Wu, L. Zhou, Y. Shao, G. Zhang, J. D. Keyyu, S. Knauf, M. D. le, E. Lizano, S. Merker, A. Navarro, T. Nadler, C. C. Khor, J. Lee, P. Tan, W. K. Lim, A. C. Kitchener, D. Zinner, I. Gut, A. D. Melin, K. Guschanski, M. H. Schierup, R. M. D. Beck, G. Umapathy, C. Roos, J. P. Boubli, J. Rogers, K. K. H. Farh, T. Marques Bonet, A global catalog of whole-genome diversity from 233 primate species. *Science* **380**, 906–913 (2023).

56. A. R. Ives, T. Garland Jr., Phylogenetic logistic regression for binary dependent variables. *Syst. Biol.* **59**, 9–26 (2010).

57. L. s. T. Ho, C. Ané, A linear-time algorithm for Gaussian and non-Gaussian trait evolution models. *Syst. Biol.* **63**, 397–408 (2014).

58. S. C. Vernes, D. F. Newbury, B. S. Abrahams, L. Winchester, J. Nicod, M. Groszer, M. Alarcón, P. L. Oliver, K. E. Davies, D. H. Geschwind, A. P. Monaco, S. E. Fisher, A functional genetic link between distinct developmental language disorders. *N. Engl. J. Med.* **359**, 2337–2345 (2008).

59. V. M. Rajagopal, A. Ganna, J. R. I. Coleman, A. Allegrini, G. Voloudakis, J. Grove, T. D. Als, H. T. Horsdal, L. Petersen, V. Appadurai, A. Schork, A. Buil, C. M. Bulik, J. Bybjerg-

Grauholm, M. Bækvad-Hansen, D. M. Hougaard, O. Mors, M. Nordentoft, T. Werge, iPSYCH-Broad Consortium, R. Belliveau, C. E. Carey, F. Cerrato, K. Chambert, C. Churchhouse, M. J. Daly, A. Dumont, J. Goldstein, C. S. Hansen, D. P. Howrigan, H. Huang, J. Maller, A. R. Martin, J. Martin, M. Mattheisen, J. Moran, B. M. Neale, J. Pallesen, D. S. Palmer, C. B. Pedersen, M. G. Pedersen, T. Poterba, S. Ripke, F. K. Satterstrom, W. K. Thompson, P. Turley, R. K. Walters, P. B. Mortensen, G. Breen, P. Roussos, R. Plomin, E. Agerbo, A. D. Børglum, D. Demontis, Genome-wide association study of school grades identifies genetic overlap between language ability, psychopathology and creativity. *Sci. Rep.* **13**, 429 (2023).

60. S. L. Hickey, S. Berto, G. Konopka, Chromatin decondensation by FOXP2 promotes human neuron maturation and expression of neurodevelopmental disease genes. *Cell Rep.* **27**, 1699–1711.e9 (2019).

61. S. A. Lambert, A. Jolma, L. F. Campitelli, P. K. das, Y. Yin, M. Albu, X. Chen, J. Taipale, T. R. Hughes, M. T. Weirauch, The human transcription factors. *Cell* **172**, 650–665 (2018).

62. O. Hobert, Homeobox genes and the specification of neuronal identity. *Nat. Rev. Neurosci.* **22**, 627–636 (2021).

63. M. Co, A. G. Anderson, G. Konopka, FOXP transcription factors in vertebrate brain development, function, and disorders. *Wiley Interdiscip. Rev. Dev. Biol.* **9**, e375 (2020).

64. B. Mariani, G. Nicoletti, G. Barzon, M. C. Ortiz Barajas, M. Shukla, R. Guevara, S. S. Suweis, J. Gervain, Prenatal experience with language shapes the brain. *Sci. Adv.* **9**, eadj3524 (2023).

65. S. Madigan, M. Wade, A. Plamondon, D. Browne, J. M. Jenkins, Birth weight variability and language development: Risk, resilience, and responsive parenting. *J. Pediatr. Psychol.* **40**, 869–877 (2015).

66. C. Sudlow, J. Gallacher, N. Allen, V. Beral, P. Burton, J. Danesh, P. Downey, P. Elliott, J. Green, M. Landray, B. Liu, P. Matthews, G. Ong, J. Pell, A. Silman, A. Young, T. Sprosen, T.

Peakman, R. Collins, UK Biobank: An open access resource for identifying the causes of a wide range of complex diseases of middle and old age. *PLOS Med.* **12**, e1001779 (2015).

67. O. Fornes, J. A. Castro-Mondragon, A. Khan, R. van der Lee, X. Zhang, P. A. Richmond, B. P. Modi, S. Correard, M. Gheorghe, D. Baranašić, W. Santana-Garcia, G. Tan, J. Chèneby, B. Ballester, F. Parcy, A. Sandelin, B. Lenhard, W. W. Wasserman, A. Mathelier, JASPAR 2020: Update of the open-access database of transcription factor binding profiles. *Nucleic Acids Res.* **48**, D87–D92 (2020).

68. K. E. Jones, J. Bielby, M. Cardillo, S. A. Fritz, J. O'Dell, C. D. L. Orme, K. Safi, W. Sechrest, E. H. Boakes, C. Carbone, C. Connolly, M. J. Cutts, J. K. Foster, R. Grenyer, M. Habib, C. A. Plaster, S. A. Price, E. A. Rigby, J. Rist, A. Teacher, O. R. P. Bininda-Emonds, J. L. Gittleman, G. M. Mace, A. Purvis, PanTHERIA: A species-level database of life history, ecology, and geography of extant and recently extinct mammals. *Ecology* **90**, 2648–2648 (2009).

69. J. B. Tomblin, N. L. Records, X. Zhang, A system for the diagnosis of specific language impairment in kindergarten children. *J. Speech Lang. Hear. Res.* **39**, 1284–1294 (1996).

70. J. B. Tomblin, M. A. Nippold, Eds., *Understanding Individual Differences in Language Development Across the School Years* (Psychology Press, 2014).

71. P. L. Newcomer, D. D. Hammill, *Test of Language Development - 2 Primary* (Pro-Ed, 1988).

72. D. Wechsler, APA PsycTests, Wechsler Preschool and Primary Scale of Intelligence—Revised (1989); https://doi.org/10.1037/t48859-000.

73. B. Culatta, J. L. Page, J. Ellis, Story retelling as a communicative performance screening tool. *Lang. Speech Hear. Serv. Sch.* **14**, 66–74 (1983).

74. R. W. Woodcock, APA PsycTests, Woodcock Reading Mastery Tests—Revised (1987), https://doi.org/10.1037/t15178-000.

75. H. W. Catts, The relationship between speech-language impairments and reading disabilities. *J. Speech Lang. Hear. Res.* **36**, 948–958 (1993).

76. T. M. Achenbach, in *Child Behavior Checklist* (Springer, 2011), pp. 546–552.

77. R Core Team, R: A Language and Environment for Statistical Computing (R Foundation for Statistical Computing, 2020); www.R-project.org/.

78. F. E. Harrell Jr., Hmisc: Harrell Miscellaneous, R package version 4.4-0 (2020); https://CRAN.R-project.org/package=Hmisc.

79. M. Mayer, missRanger: Fast Imputation of Missing Values, R package version 2.1.0 (2019); https://CRAN.R-project.org/package=missRanger.

80. S. Andrews, F. Krueger, A. Segonds-Pichon, L. Biggins, C. Krueger, S. Wingett, FastQC (Babraham Institute, 2012); http://www.bioinformatics.babraham.ac.uk/projects/fastqc/.

81. B. Chapman, Rory Kirchner, L. Pantano, T. Khotiainsteva, M. De Smet, L. Beltrame, V. Saveliev, R. V. Guimera, S. Naumenko, J. Kern, C. Brueffer, G. Carrasco, M. Giovacchini, I. Sytchev, P. Tang, M. Ahdesmaki, S. Kanwal, J. J. Porter, S. Möller, V. Le, A. Coman, V. Svensson, bogdang, M. Mistry, M. Edwards, J. Hammerbacher, B. Pedersen, P. Cock, apastore, S. Turner, bcbio/bcbio-nextgen: v1.1.9 (2019); https://doi.org/10.5281/zenodo.3564939.

82. H. Li, Aligning sequence reads, clone sequences and assembly contigs with BWA-MEM. arXiv:1303.3997 (2013).

83. A. McKenna, M. Hanna, E. Banks, A. Sivachenko, K. Cibulskis, A. Kernytsky, K. Garimella, D. Altshuler, S. Gabriel, M. Daly, M. A. DePristo, The genome analysis toolkit: A MapReduce framework for analyzing next-generation DNA sequencing data. *Genome Res.* **20**, 1297–1303 (2010).

84. E. Garrison, G. Marth, Haplotype-based variant detection from short-read sequencing. arXiv:1207.3907 (2012).

85. A. Rimmer, H. Phan, I. Mathieson, Z. Iqbal, S. R. F. Twigg, WGS500 Consortium, A. O. M. Wilkie, G. M. Vean, G. Lunter, Integrating mapping-, assembly- and haplotype-based

approaches for calling variants in clinical sequencing applications. *Nat. Genet.* **46**, 912–918 (2014).

86.1000G enomes Project Consortium, A. Auton, L. D. Brooks, R. M. Durbin, E. P. Garrison, H. M. Kang, J. O. Korbel, J. L. Marchini, S. M. Carthy, G. A. M. Vean, G. R. Abecasis, A global reference for human genetic variation. *Nature* **526**, 68–74 (2015).

87. W. McLaren, L. Gil, S. E. Hunt, H. S. Riat, G. R. S. Ritchie, A. Thormann, P. Flicek, F. Cunningham, The ensembl variant effect predictor. *Genome Biol.* **17**, 122 (2016).

88. K. J. Karczewski, L. C. Francioli, G. Tiao, B. B. Cummings, J. Alföldi, Q. Wang, R. L. Collins, K. M. Laricchia, A. Ganna, D. P. Birnbaum, L. D. Gauthier, H. Brand, M. Solomonson, N. A. Watts, D. Rhodes, M. Singer-Berk, E. M. England, E. G. Seaby, J. A. Kosmicki, R. K. Walters, K. Tashman, Y. Farjoun, E. Banks, T. Poterba, A. Wang, C. Seed, N. Whiffin, J. X. Chong, K. E. Samocha, E. Pierce-Hoffman, Z. Zappala, A. H. O'Donnell-Luria, E. V. Minikel, B. Weisburd, M. Lek, J. S. Ware, C. Vittal, I. M. Armean, L. Bergelson, K. Cibulskis, K. M. Connolly, M. Covarrubias, S. Donnelly, S. Ferriera, S. Gabriel, J. Gentry, N. Gupta, T. Jeandet, D. Kaplan, C. Llanwarne, R. Munshi, S. Novod, N. Petrillo, D. Roazen, V. Ruano-Rubio, A. Saltzman, M. Schleicher, J. Soto, K. Tibbetts, C. Tolonen, G. Wade, M. E. Talkowski, Genome Aggregation Database Consortium, B. M. Neale, M. J. Daly, D. G. M. Arthur, The mutational constraint spectrum quantified from variation in 141,456 humans. *Nature* **581**, 434–443 (2020).

89. E. M. Smigielski, dbSNP: A database of single nucleotide polymorphisms. *Nucleic Acids Res.* **28**, 352–355 (2000).

90. B. S. Pedersen, R. M. Layer, A. R. Quinlan, Vcfanno: Fast, flexible annotation of genetic variants. *Genome Biol.* **17**, 118 (2016).

91. S. W. Choi, T. S.-H. Mak, P. F. O'Reilly, Tutorial: A guide to performing polygenic risk score analyses. *Nat. Protoc.* **15**, 2759–2772 (2020).

92. F. Privé, C. Albiñana, J. Arbel, B. Pasaniuc, B. J. Vilhjálmsson, Inferring disease architecture and predictive ability with LDpred2-auto. *Am. J. Human Genet.* **110**, 2042–2055 (2023).

93. D. Demontis, R. K. Walters, J. Martin, M. Mattheisen, T. D. Als, E. Agerbo, G. Baldursson, R. Belliveau, J. Bybjerg-Grauholm, M. Bækvad-Hansen, F. Cerrato, K. Chambert, C. Churchhouse, A. Dumont, N. Eriksson, M. Gandal, J. I. Goldstein, K. L. Grasby, J. Grove, O. O. Gudmundsson, C. S. Hansen, M. E. Hauberg, M. V. Hollegaard, D. P. Howrigan, H. Huang, J. B. Maller, A. R. Martin, N. G. Martin, J. Moran, J. Pallesen, D. S. Palmer, C. B. Pedersen, M. G. Pedersen, T. Poterba, J. B. Poulsen, S. Ripke, E. B. Robinson, F. K. Satterstrom, H. Stefansson, C. Stevens, P. Turley, G. B. Walters, H. Won, M. J. Wright, ADHD Working Group of the Psychiatric Genomics Consortium (PGC), Early Lifecourse and Genetic Epidemiology (EAGLE) Consortium, 23andMe Research Team, O. A. Andreassen, P. Asherson, C. L. Burton, D. I. Boomsma, B. Cormand, S. Dalsgaard, B. Franke, J. Gelernter, D. Geschwind, H. Hakonarson, J. Haavik, H. R. Kranzler, J. Kuntsi, K. Langley, K.-P. Lesch, C. Middeldorp, A. Reif, L. A. Rohde, P. Roussos, R. Schachar, P. Sklar, E. J. S. Sonuga-Barke, P. F. Sullivan, A. Thapar, J. Y. Tung, I. D. Waldman, S. E. Medland, K. Stefansson, M. Nordentoft, D. M. Hougaard, T. Werge, O. Mors, P. B. Mortensen, M. J. Daly, S. V. Faraone, A. D. Børglum, B. M. Neale, Discovery of the first genome-wide significant risk loci for attention deficit/hyperactivity disorder. *Nat. Genet.* **51**, 63–75 (2019).

94. A. S. Hatoum, S. M. C. Colbert, E. C. Johnson, S. B. Huggett, J. D. Deak, G. A. Pathak, M. V. Jennings, S. E. Paul, N. R. Karcher, I. Hansen, D. A. A. Baranger, A. Edwards, A. D. Grotzinger, Substance Use Disorder Working Group of the Psychiatric Genomics Consortium, D. E. Adkins, A. E. Adkins, M. Alanne-Kinnunen, J. C. Alexander, F. Aliev, S. A. Bacanu, A. Batzler, J. M. Biernacka, L. J. Bierut, T. B. Bigdeli, A. Blagonravova, J. D. Boardman, J. M. Boden, D. I. Boomsma, S. A. Brown, K. K. Bucholz, D. Chen, L. S. Chen, D. S. Choi, S. P. Chou, S. Cichon, W. E. Copeland, R. P. Corley, F. Degenhardt, M. di Forti, N. Diazgranados, D. M. Dick, B. W. Domingue, J. G. Eriksson, L. A. Farrer, J. C. Foo, T. M. Foroud, L. Fox, J. Frank, M. A. Frye, W. Gaebel, R. R. Gainetdinov, I. Giegling, N. A. Gillespie, A. M. Goate, D. Goldman, S. Gordon, L. M. Hack, D. B. Hancock, K. M. Harris, A. M. Hartmann, A. C. Heath, S. Heilmann-Heimbach, S. Herms, V. Hesselbrock, J. K. Hewitt, I. Hickie, C. Hodgkinson, P. Hoffmann, C. Hopfer, J. Horwood, J. J. Hottenga, D. P. Howrigan, W. G. Iacono, M. Ising, E. O. Johnson, J. Kaprio, V. M. Karpyak, K. S. Kendler, M. A. Kennedy, M. Keyes, A. Kibitov, F. Kiefer, B. Konte, J. Kramer, K. Krauter, E. M. Krupitsky, S. Kuperman, J. Lahti, M. Lahti-Pulkkinen, D. Lai, A. Levchenko, L. Ligthart, P. A. Lind, S. Lucae, M. T. Lynskey, P. A. F. Madden, H. H. Maes, P. K. E. Magnusson, B. S.

Maher, K. Mann, S. Männistö, N. G. Martin, H. Mbarek, M. McGue, M. B. McQueen, S. E. Medland, J. L. Meyers, G. W. Montgomery, B. Müller-Myhsok, B. M. Neale, E. C. Nelson, M. M. Nöthen, J. I. Nurnberger, A. Palotie, T. Palviainen, J. F. Pearson, N. L. Pedersen, B. W. J. H. Penninx, R. E. Peterson, B. Porjesz, U. W. Preuss, D. Quattrone, K. Räikkönen, M. D. Reynolds, J. P. Rice, M. Ridinger, M. Rietschel, B. P. Riley, S. Ripatti, R. J. Rose, D. Rujescu, K. V. Rybakova, E. Ryu, N. L. Saccone, J. E. Salvatore, N. Scherbaum, M. A. Schuckit, M. Schwandt, P. H. Shen, R. Sherva, J. Silberg, M. C. Stallings, D. J. Stein, F. Streit, J. Strohmaier, R. E. Tarter, N. Thomas, M. M. Vanyukov, S. Vrieze, T. L. Wall, R. K. Walters, B. T. Webb, R. Wedow, F. Wendt, L. Wetherill, J. B. Whitfield, S. Witt, N. Wodarz, M. J. Wright, S. M. Hartz, S. Zellers, H. Zhang, H. Zhao, H. Zhou, P. Zill, L. Zillich, E. M. Tucker-Drob, H. R. Kranzler, L. K. Davis, S. Sanchez-Roige, R. Polimanti, J. Gelernter, H. J. Edenberg, R. Bogdan, A. Agrawal, Multivariate genome-wide association meta-analysis of over 1 million subjects identifies loci underlying multiple substance use disorders. *Nat. Ment. Health* **1**, 210–223 (2023).

95. R. K. Walters, R. Polimanti, E. C. Johnson, J. N. M. Clintick, M. J. Adams, A. E. Adkins, F. Aliev, S.-A. Bacanu, A. Batzler, S. Bertelsen, J. M. Biernacka, T. B. Bigdeli, L.-S. Chen, T.-K. Clarke, Y.-L. Chou, F. Degenhardt, A. R. Docherty, A. C. Edwards, P. Fontanillas, J. C. Foo, L. Fox, J. Frank, I. Giegling, S. Gordon, L. M. Hack, A. M. Hartmann, S. M. Hartz, S. Heilmann-Heimbach, S. Herms, C. Hodgkinson, P. Hoffmann, J. J. Hottenga, M. A. Kennedy, M. Alanne-Kinnunen, B. Konte, J. Lahti, M. Lahti-Pulkkinen, D. Lai, L. Ligthart, A. Loukola, B. S. Maher, H. Mbarek, A. M. McIntosh, M. B. McQueen, J. L. Meyers, Y. Milaneschi, T. Palviainen, J. F. Pearson, R. E. Peterson, S. Ripatti, E. Ryu, N. L. Saccone, J. E. Salvatore, S. Sanchez-Roige, M. Schwandt, R. Sherva, F. Streit, J. Strohmaier, N. Thomas, J.-C. Wang, B. T. Webb, R. Wedow, L. Wetherill, A. G. Wills, 23andMe Research Team, J. D. Boardman, D. Chen, D.-S. Choi, W. E. Copeland, R. C. Culverhouse, N. Dahmen, L. Degenhardt, B. W. Domingue, S. L. Elson, M. A. Frye, W. Gäbel, C. Hayward, M. Ising, M. Keyes, F. Kiefer, J. Kramer, S. Kuperman, S. Lucae, M. T. Lynskey, W. Maier, K. Mann, S. Männistö, B. Müller-Myhsok, A. D. Murray, J. I. Nurnberger, A. Palotie, U. Preuss, K. Räikkönen, M. D. Reynolds, M. Ridinger, N. Scherbaum, M. A. Schuckit, M. Soyka, J. Treutlein, S. Witt, N. Wodarz, P. Zill, D. E. Adkins, J. M. Boden, D. I. Boomsma, L. J. Bierut, S. A. Brown, K. K. Bucholz, S. Cichon, E. J. Costello, H. de Wit, N. Diazgranados, D. M. Dick, J. G. Eriksson, L. A. Farrer, T. M. Foroud, N. A. Gillespie, A. M. Goate, D. Goldman,

R. A. Grucza, D. B. Hancock, K. M. Harris, A. C. Heath, V. Hesselbrock, J. K. Hewitt, C. J. Hopfer, J. Horwood, W. Iacono, E. O. Johnson, J. A. Kaprio, V. M. Karpyak, K. S. Kendler, H. R. Kranzler, K. Krauter, P. Lichtenstein, P. A. Lind, M. M. Gue, J. M. Killop, P. A. F. Madden, H. H. Maes, P. Magnusson, N. G. Martin, S. E. Medland, G. W. Montgomery, E. C. Nelson, M. M. Nöthen, A. A. Palmer, N. L. Pedersen, B. W. J. H. Penninx, B. Porjesz, J. P. Rice, M. Rietschel, B. P. Riley, R. Rose, D. Rujescu, P.-H. Shen, J. Silberg, M. C. Stallings, R. E. Tarter, M. M. Vanyukov, S. Vrieze, T. L. Wall, J. B. Whitfield, H. Zhao, B. M. Neale, J. Gelernter, H. J. Edenberg, A. Agrawal, Transancestral GWAS of alcohol dependence reveals common genetic underpinnings with psychiatric disorders. *Nat. Neurosci.* **21**, 1656–1669 (2018).

96. D. P. Wightman, I. E. Jansen, J. E. Savage, A. A. Shadrin, S. Bahrami, D. Holland, A. Rongve, S. Børte, B. S. Winsvold, O. K. Drange, A. E. Martinsen, A. H. Skogholt, C. Willer, G. Bråthen, I. Bosnes, J. B. Nielsen, L. G. Fritsche, L. F. Thomas, L. M. Pedersen, M. E. Gabrielsen, M. B. Johnsen, T. W. Meisingset, W. Zhou, P. Proitsi, A. Hodges, R. Dobson, L. Velayudhan, K. Heilbron, A. Auton, 23andMe Research Team, M. Agee, S. Aslibekyan, E. Babalola, R. K. Bell, J. Bielenberg, K. Bryc, E. Bullis, B. Cameron, D. Coker, G. C. Partida, D. Dhamija, S. das, S. L. Elson, T. Filshtein, K. Fletez-Brant, P. Fontanillas, W. Freyman, P. M. Gandhi, B. Hicks, D. A. Hinds, K. E. Huber, E. M. Jewett, Y. Jiang, A. Kleinman, K. Kukar, V. Lane, K. H. Lin, M. Lowe, M. K. Luff, J. C. McCreight, M. H. McIntyre, K. F. McManus, S. J. Micheletti, M. E. Moreno, J. L. Mountain, S. V. Mozaffari, P. Nandakumar, E. S. Noblin, J. O'Connell, A. A. Petrakovitz, G. D. Poznik, M. Schumacher, A. J. Shastri, J. F. Shelton, J. Shi, S. Shringarpure, C. Tian, V. Tran, J. Y. Tung, X. Wang, W. Wang, C. H. Weldon, P. Wilton, J. M. Sealock, L. K. Davis, N. L. Pedersen, C. A. Reynolds, I. K. Karlsson, S. Magnusson, H. Stefansson, S. Thordardottir, P. V. Jonsson, J. Snaedal, A. Zettergren, I. Skoog, S. Kern, M. Waern, H. Zetterberg, K. Blennow, E. Stordal, K. Hveem, J. A. Zwart, L. Athanasiu, P. Selnes, I. Saltvedt, S. B. Sando, I. Ulstein, S. Djurovic, T. Fladby, D. Aarsland, G. Selbæk, S. Ripke, K. Stefansson, O. A. Andreassen, D. Posthuma, A genome-wide association study with 1,126,563 individuals identifies new risk loci for Alzheimer's disease. *Nat. Genet.* **53**, 1276–1282 (2021).

97. J. Grove, S. Ripke, T. D. Als, M. Mattheisen, R. K. Walters, H. Won, J. Pallesen, E. Agerbo, O. A. Andreassen, R. Anney, S. Awashti, R. Belliveau, F. Bettella, J. D. Buxbaum, J. Bybjerg-

Grauholm, M. Bækvad-Hansen, F. Cerrato, K. Chambert, J. H. Christensen, C. Churchhouse, K. Dellenvall, D. Demontis, S. De Rubeis, B. Devlin, S. Djurovic, A. L. Dumont, J. I. Goldstein, C. S. Hansen, M. E. Hauberg, M. V. Hollegaard, S. Hope, D. P. Howrigan, H. Huang, C. M. Hultman, L. Klei, J. Maller, J. Martin, A. R. Martin, J. L. Moran, M. Nyegaard, T. Nærland, D. S. Palmer, A. Palotie, C. B. Pedersen, M. G. Pedersen, T. dPoterba, J. B. Poulsen, B. S. Pourcain, P. Qvist, K. Rehnström, A. Reichenberg, J. Reichert, E. B. Robinson, K. Roeder, P. Roussos, E. Saemundsen, S. Sandin, F. K. Satterstrom, G. D. Smith, H. Stefansson, S. Steinberg, C. R. Stevens, P. F. Sullivan, P. Turley, G. B. Walters, X. Xu, Autism Spectrum Disorder Working Group of the Psychiatric Genomics Consortium, BUPGEN, Major Depressive Disorder Working Group of the Psychiatric Genomics Consortium, 23andMe Research Team, K. Stefansson, D. H. Geschwind, M. Nordentoft, D. M. Hougaard, T. Werge, O. Mors, P. B. Mortensen, B. M. Neale, M. J. Daly, A. D. Børglum, Identification of common genetic risk variants for autism spectrum disorder. *Nat. Genet.* **51**, 431–444 (2019).

98. H. J. Watson, Z. Yilmaz, L. M. Thornton, C. Hübel, J. R. I. Coleman, H. A. Gaspar, J. Bryois, A. Hinney, V. M. Leppä, M. Mattheisen, S. E. Medland, S. Ripke, S. Yao, P. Giusti-Rodríguez, Anorexia Nervosa Genetics Initiative, K. B. Hanscombe, K. L. Purves, Eating Disorders Working Group of the Psychiatric Genomics Consortium, R. A. H. Adan, L. Alfredsson, T. Ando, O. A. Andreassen, J. H. Baker, W. H. Berrettini, I. Boehm, C. Boni, V. B. Perica, K. Buehren, R. Burghardt, M. Cassina, S. Cichon, M. Clementi, R. D. Cone, P. Courtet, S. Crow, J. J. Crowley, U. N. Danner, O. S. P. Davis, M. de Zwaan, G. Dedoussis, D. Degortes, J. E. De Socio, D. M. Dick, D. Dikeos, C. Dina, M. Dmitrzak-Weglarz, E. Docampo, L. E. Duncan, K. Egberts, S. Ehrlich, G. Escaramís, T. Esko, X. Estivill, A. Farmer, A. Favaro, F. Fernández-Aranda, M. M. Fichter, K. Fischer, M. Föcker, L. Foretova, A. J. Forstner, M. Forzan, C. S. Franklin, S. Gallinger, I. Giegling, J. Giuranna, F. Gonidakis, P. Gorwood, M. G. Mayora, S. Guillaume, Y. Guo, H. Hakonarson, K. Hatzikotoulas, J. Hauser, J. Hebebrand, S. G. Helder, S. Herms, B. Herpertz-Dahlmann, W. Herzog, L. M. Huckins, J. I. Hudson, H. Imgart, H. Inoko, V. Janout, S. Jiménez-Murcia, A. Julià, G. Kalsi, D. Kaminská, J. Kaprio, L. Karhunen, A. Karwautz, M. J. H. Kas, J. L. Kennedy, A. Keski-Rahkonen, K. Kiezebrink, Y.-R. Kim, L. Klareskog, K. L. Klump, G. P. S. Knudsen, M. C. La Via, S. L. Hellard, R. D. Levitan, D. Li, L. Lilenfeld, B. D. Lin, J. Lissowska, J. Luykx, P. J. Magistretti, M. Maj, K. Mannik, S. Marsal, C. R. Marshall, M. Mattingsdal, S. M. Devitt,

P. M. Guffin, A. Metspalu, I. Meulenbelt, N. Micali, K. Mitchell, A. M. Monteleone, P. Monteleone, M. A. Munn-Chernoff, B. Nacmias, M. Navratilova, I. Ntalla, J. K. O'Toole, R. A. Ophoff, L. Padyukov, A. Palotie, J. Pantel, H. Papezova, D. Pinto, R. Rabionet, A. Raevuori, N. Ramoz, T. Reichborn-Kjennerud, V. Ricca, S. Ripatti, F. Ritschel, M. Roberts, A. Rotondo, D. Rujescu, F. Rybakowski, P. Santonastaso, A. Scherag, S. W. Scherer, U. Schmidt, N. J. Schork, A. Schosser, J. Seitz, L. Slachtova, P. E. Slagboom, M. C. T. Slof-Op 't Landt, A. Slopien, S. Sorbi, B. Świątkowska, J. P. Szatkiewicz, I. Tachmazidou, E. Tenconi, A. Tortorella, F. Tozzi, J. Treasure, A. Tsitsika, M. Tyszkiewicz-Nwafor, K. Tziouvas, A. A. van Elburg, E. F. van Furth, G. Wagner, E. Walton, E. Widen, E. Zeggini, S. Zerwas, S. Zipfel, A. W. Bergen, J. M. Boden, H. Brandt, S. Crawford, K. A. Halmi, L. J. Horwood, C. Johnson, A. S. Kaplan, W. H. Kaye, J. E. Mitchell, C. M. Olsen, J. F. Pearson, N. L. Pedersen, M. Strober, T. Werge, D. C. Whiteman, D. B. Woodside, G. D. Stuber, S. Gordon, J. Grove, A. K. Henders, A. Juréus, K. M. Kirk, J. T. Larsen, R. Parker, L. Petersen, J. Jordan, M. Kennedy, G. W. Montgomery, T. D. Wade, A. Birgegård, P. Lichtenstein, C. Norring, M. Landén, N. G. Martin, P. B. Mortensen, P. F. Sullivan, G. Breen, Genome-wide association study identifies eight risk loci and implicates metabo-psychiatric origins for anorexia nervosa. *Nat. Genet.* **51**, 1207–1214 (2019).

99. N. Mullins, A. J. Forstner, K. S. O'Connell, B. Coombes, J. R. I. Coleman, Z. Qiao, T. D. Als, T. B. Bigdeli, S. Børte, J. Bryois, A. W. Charney, O. K. Drange, M. J. Gandal, S. P. Hagenaars, M. Ikeda, N. Kamitaki, M. Kim, K. Krebs, G. Panagiotaropoulou, B. M. Schilder, L. G. Sloofman, S. Steinberg, V. Trubetskoy, B. S. Winsvold, H. H. Won, L. Abramova, K. Adorjan, E. Agerbo, M. al Eissa, D. Albani, N. Alliey-Rodriguez, A. Anjorin, V. Antilla, A. Antoniou, S. Awasthi, J. H. Baek, M. Bækvad-Hansen, N. Bass, M. Bauer, E. C. Beins, S. E. Bergen, A. Birner, C. Bøcker Pedersen, E. Bøen, M. P. Boks, R. Bosch, M. Brum, B. M. Brumpton, N. Brunkhorst-Kanaan, M. Budde, J. Bybjerg-Grauholm, W. Byerley, M. Cairns, M. Casas, P. Cervantes, T. K. Clarke, C. Cruceanu, A. Cuellar-Barboza, J. Cunningham, D. Curtis, P. M. Czerski, A. M. Dale, N. Dalkner, F. S. David, F. Degenhardt, S. Djurovic, A. L. Dobbyn, A. Douzenis, T. Elvsåshagen, V. Escott-Price, I. N. Ferrier, A. Fiorentino, T. M. Foroud, L. Forty, J. Frank, O. Frei, N. B. Freimer, L. Frisén, K. Gade, J. Garnham, J. Gelernter, M. Giørtz Pedersen, I. R. Gizer, S. D. Gordon, K. Gordon-Smith, T. A. Greenwood, J. Grove, J. Guzman-Parra, K. Ha, M. Haraldsson, M. Hautzinger, U. Heilbronner, D. Hellgren, S. Herms, P. Hoffmann, P. A. Holmans, L. Huckins, S. Jamain, J. S.

Johnson, J. L. Kalman, Y. Kamatani, J. L. Kennedy, S. Kittel-Schneider, J. A. Knowles, M. Kogevinas, M. Koromina, T. M. Kranz, H. R. Kranzler, M. Kubo, R. Kupka, S. A. Kushner, C. Lavebratt, J. Lawrence, M. Leber, H. J. Lee, P. H. Lee, S. E. Levy, C. Lewis, C. Liao, S. Lucae, M. Lundberg, D. J. MacIntyre, S. H. Magnusson, W. Maier, A. Maihofer, D. Malaspina, E. Maratou, L. Martinsson, M. Mattheisen, S. A. McCarroll, N. W. McGregor, P. McGuffin, J. D. McKay, H. Medeiros, S. E. Medland, V. Millischer, G. W. Montgomery, J. L. Moran, D. W. Morris, T. W. Mühleisen, N. O'Brien, C. O'Donovan, L. M. Olde Loohuis, L. Oruc, S. Papiol, A. F. Pardiñas, A. Perry, A. Pfennig, E. Porichi, J. B. Potash, D. Quested, T. Raj, M. H. Rapaport, J. R. DePaulo, E. J. Regeer, J. P. Rice, F. Rivas, M. Rivera, J. Roth, P. Roussos, D. M. Ruderfer, C. Sánchez-Mora, E. C. Schulte, F. Senner, S. Sharp, P. D. Shilling, E. Sigurdsson, L. Sirignano, C. Slaney, O. B. Smeland, D. J. Smith, J. L. Sobell, C. Søholm Hansen, M. Soler Artigas, A. T. Spijker, D. J. Stein, J. S. Strauss, B. Świątkowska, C. Terao, T. E. Thorgeirsson, C. Toma, P. Tooney, E. E. Tsermpini, M. P. Vawter, H. Vedder, J. T. R. Walters, S. H. Witt, S. Xi, W. Xu, J. M. K. Yang, A. H. Young, H. Young, P. P. Zandi, H. Zhou, L. Zillich, HUNT All-In Psychiatry, R. Adolfsson, I. Agartz, M. Alda, L. Alfredsson, G. Babadjanova, L. Backlund, B. T. Baune, F. Bellivier, S. Bengesser, W. H. Berrettini, D. H. R. Blackwood, M. Boehnke, A. D. Børglum, G. Breen, V. J. Carr, S. Catts, A. Corvin, N. Craddock, U. Dannlowski, D. Dikeos, T. Esko, B. Etain, P. Ferentinos, M. Frye, J. M. Fullerton, M. Gawlik, E. S. Gershon, F. S. Goes, M. J. Green, M. Grigoroiu-Serbanescu, J. Hauser, F. Henskens, J. Hillert, K. S. Hong, D. M. Hougaard, C. M. Hultman, K. Hveem, N. Iwata, A. V. Jablensky, I. Jones, L. A. Jones, R. S. Kahn, J. R. Kelsoe, G. Kirov, M. Landén, M. Leboyer, C. M. Lewis, Q. S. Li, J. Lissowska, C. Lochner, C. Loughland, N. G. Martin, C. A. Mathews, F. Mayoral, S. L. McElroy, A. M. McIntosh, F. J. McMahon, I. Melle, P. Michie, L. Milani, P. B. Mitchell, G. Morken, O. Mors, P. B. Mortensen, B. Mowry, B. Müller-Myhsok, R. M. Myers, B. M. Neale, C. M. Nievergelt, M. Nordentoft, M. M. Nöthen, M. C. O'Donovan, K. J. Oedegaard, T. Olsson, M. J. Owen, S. A. Paciga, C. Pantelis, C. Pato, M. T. Pato, G. P. Patrinos, R. H. Perlis, D. Posthuma, J. A. Ramos-Quiroga, A. Reif, E. Z. Reininghaus, M. Ribasés, M. Rietschel, S. Ripke, G. A. Rouleau, T. Saito, U. Schall, M. Schalling, P. R. Schofield, T. G. Schulze, L. J. Scott, R. J. Scott, A. Serretti, C. Shannon Weickert, J. W. Smoller, H. Stefansson, K. Stefansson, E. Stordal, F. Streit, P. F. Sullivan, G. Turecki, A. E. Vaaler, E. Vieta, J. B. Vincent, I. D. Waldman, T. W. Weickert, T. Werge, N. R. Wray, J. A. Zwart, J. M. Biernacka, J. I. Nurnberger, S. Cichon, H. J. Edenberg, E. A. Stahl,

A. McQuillin, A. di Florio, R. A. Ophoff, O. A. Andreassen, Genome-wide association study of more than 40,000 bipolar disorder cases provides new insights into the underlying biology. *Nat. Genet.* **53**, 817–829 (2021).

100. E. C. Johnson, D. Demontis, T. E. Thorgeirsson, R. K. Walters, R. Polimanti, A. S. Hatoum, S. Sanchez-Roige, S. E. Paul, F. R. Wendt, T. K. Clarke, D. Lai, G. W. Reginsson, H. Zhou, J. He, D. A. A. Baranger, D. F. Gudbjartsson, R. Wedow, D. E. Adkins, A. E. Adkins, J. Alexander, S. A. Bacanu, T. B. Bigdeli, J. Boden, S. A. Brown, K. K. Bucholz, J. Bybjerg-Grauholm, R. P. Corley, L. Degenhardt, D. M. Dick, B. W. Domingue, L. Fox, A. M. Goate, S. D. Gordon, L. M. Hack, D. B. Hancock, S. M. Hartz, I. B. Hickie, D. M. Hougaard, K. Krauter, P. A. Lind, J. N. McClintick, M. B. McQueen, J. L. Meyers, G. W. Montgomery, O. Mors, P. B. Mortensen, M. Nordentoft, J. F. Pearson, R. E. Peterson, M. D. Reynolds, J. P. Rice, V. Runarsdottir, N. L. Saccone, R. Sherva, J. L. Silberg, R. E. Tarter, T. Tyrfingsson, T. L. Wall, B. T. Webb, T. Werge, L. Wetherill, M. J. Wright, S. Zellers, M. J. Adams, L. J. Bierut, J. D. Boardman, W. E. Copeland, L. A. Farrer, T. M. Foroud, N. A. Gillespie, R. A. Grucza, K. M. Harris, A. C. Heath, V. Hesselbrock, J. K. Hewitt, C. J. Hopfer, J. Horwood, W. G. Iacono, E. O. Johnson, K. S. Kendler, M. A. Kennedy, H. R. Kranzler, P. A. F. Madden, H. H. Maes, B. S. Maher, N. G. Martin, M. McGue, A. M. McIntosh, S. E. Medland, E. C. Nelson, B. Porjesz, B. P. Riley, M. C. Stallings, M. M. Vanyukov, S. Vrieze, R. Walters, R. Polimanti, E. Johnson, J. McClintick, A. Hatoum, J. He, F. Wendt, H. Zhou, M. Adams, A. Adkins, F. Aliev, S. A. Bacanu, A. Batzler, S. Bertelsen, J. Biernacka, T. Bigdeli, L. S. Chen, T. K. Clarke, Y. L. Chou, F. Degenhardt, A. Docherty, A. Edwards, P. Fontanillas, J. Foo, L. Fox, J. Frank, I. Giegling, S. Gordon, L. Hack, A. Hartmann, S. Hartz, S. Heilmann-Heimbach, S. Herms, C. Hodgkinson, P. Hoffman, J. Hottenga, M. Kennedy, M. Alanne-Kinnunen, B. Konte, J. Lahti, M. Lahti-Pulkkinen, D. Lai, L. Ligthart, A. Loukola, B. Maher, H. Mbarek, A. McIntosh, M. McQueen, J. Meyers, Y. Milaneschi, T. Palviainen, J. Pearson, R. Peterson, S. Ripatti, E. Ryu, N. Saccone, J. Salvatore, S. Sanchez-Roige, M. Schwandt, R. Sherva, F. Streit, J. Strohmaier, N. Thomas, J. C. Wang, B. Webb, R. Wedow, L. Wetherill, A. Wills, J. Boardman, D. Chen, D. S. Choi, W. Copeland, R. Culverhouse, N. Dahmen, L. Degenhardt, B. Domingue, S. Elson, M. Frye, W. Gäbel, C. Hayward, M. Ising, M. Keyes, F. Kiefer, J. Kramer, S. Kuperman, S. Lucae, M. Lynskey, W. Maier, K. Mann, S. Männistö, B. Müller-Myhsok, A. Murray, J. Nurnberger, A. Palotie, U. Preuss, K. Räikkönen, M. Reynolds, M. Ridinger, N. Scherbaum, M. Schuckit, M. Soyka, J.

Treutlein, S. Witt, N. Wodarz, P. Zill, D. Adkins, J. Boden, D. Boomsma, L. Bierut, S. Brown, K. Bucholz, S. Cichon, E. J. Costello, H. de Wit, N. Diazgranados, D. Dick, J. Eriksson, L. Farrer, T. Foroud, N. Gillespie, A. Goate, D. Goldman, R. Grucza, D. Hancock, K. M. Harris, A. Heath, V. Hesselbrock, J. Hewitt, C. Hopfer, J. Horwood, W. Iacono, E. Johnson, J. Kaprio, V. Karpyak, K. Kendler, H. Kranzler, K. Krauter, P. Lichtenstein, P. Lind, M. McGue, J. MacKillop, P. Madden, H. Maes, P. Magnusson, N. Martin, S. Medland, G. Montgomery, E. Nelson, M. Nöthen, A. Palmer, N. Pederson, B. Penninx, B. Porjesz, J. Rice, M. Rietschel, B. Riley, R. Rose, D. Rujescu, P. H. Shen, J. Silberg, M. Stallings, R. Tarter, M. Vanyukov, S. Vrieze, T. Wall, J. Whitfield, H. Zhao, B. Neale, J. Gelernter, H. Edenberg, A. Agrawal, L. K. Davis, R. Bogdan, J. Gelernter, H. J. Edenberg, K. Stefansson, A. D. Børglum, A. Agrawal, A large-scale genome-wide association study meta-analysis of cannabis use disorder. *Lancet Psychiatry* **7**, 1032–1045 (2020).

101. D. M. Howard, M. J. Adams, T. K. Clarke, J. D. Hafferty, J. Gibson, M. Shirali, J. R. I. Coleman, S. P. Hagenaars, J. Ward, E. M. Wigmore, C. Alloza, X. Shen, M. C. Barbu, E. Y. Xu, H. C. Whalley, R. E. Marioni, D. J. Porteous, G. Davies, I. J. Deary, G. Hemani, K. Berger, H. Teismann, R. Rawal, V. Arolt, B. T. Baune, U. Dannlowski, K. Domschke, C. Tian, D. A. Hinds, 23andMe Research Team, Major Depressive Disorder Working Group of the Psychiatric Genomics Consortium, M. Trzaskowski, E. M. Byrne, S. Ripke, D. J. Smith, P. F. Sullivan, N. R. Wray, G. Breen, C. M. Lewis, A. M. McIntosh, Genome-wide meta-analysis of depression identifies 102 independent variants and highlights the importance of the prefrontal brain regions. *Nat. Neurosci.* **22**, 343–352 (2019).

102. International League Against Epilepsy Consortium on Complex Epilepsies, GWAS meta-analysis of over 29,000 people with epilepsy identifies 26 risk loci and subtype-specific genetic architecture. *Nat. Genet.* **55**, 1471–1482 (2023).

103. K. Watanabe, P. R. Jansen, J. E. Savage, P. Nandakumar, X. Wang, 23andMe Research Team, M. Agee, S. Aslibekyan, A. Auton, R. K. Bell, K. Bryc, S. K. Clark, S. L. Elson, K. Fletez-Brant, P. Fontanillas, N. A. Furlotte, P. M. Gandhi, K. Heilbron, B. Hicks, K. E. Huber, E. M. Jewett, Y. Jiang, A. Kleinman, K. H. Lin, N. K. Litterman, J. C. McCreight, M. H. McIntyre, K. F. McManus, J. L. Mountain, S. V. Mozaffari, E. S. Noblin, C. A. M. Northover, J. O'Connell, S. J. Pitts, G. D. Poznik, J. F. Sathirapongsasuti, J. F. Shelton, J.

Shi, S. Shringarpure, C. Tian, J. Y. Tung, R. J. Tunney, V. Vacic, W. Wang, D. A. Hinds, J. Gelernter, D. F. Levey, R. Polimanti, M. B. Stein, E. J. W. van Someren, A. B. Smit, D. Posthuma, Genome-wide meta-analysis of insomnia prioritizes genes associated with metabolic and psychiatric pathways. *Nat. Genet.* **54**, 1125–1132 (2022).

104. Q. Q. Huang, E. M. Wigdor, D. S. Malawsky, P. Campbell, K. E. Samocha, V. K. Chundru, P. Danecek, S. Lindsay, T. Marchant, M. Koko, S. Amanat, D. Bonfanti, E. Sheridan, E. J. Radford, J. C. Barrett, C. F. Wright, H. V. Firth, V. Warrier, A. Strudwick Young, M. E. Hurles, H. C. Martin, Examining the role of common variants in rare neurodevelopmental conditions. *Nature* **636**, 404–411 (2024).

105. C. M. Nievergelt, A. X. Maihofer, T. Klengel, E. G. Atkinson, C. Y. Chen, K. W. Choi, J. R. I. Coleman, S. Dalvie, L. E. Duncan, J. Gelernter, D. F. Levey, M. W. Logue, R. Polimanti, A. C. Provost, A. Ratanatharathorn, M. B. Stein, K. Torres, A. E. Aiello, L. M. Almli, A. B. Amstadter, S. B. Andersen, O. A. Andreassen, P. A. Arbisi, A. E. Ashley-Koch, S. B. Austin, E. Avdibegovic, D. Babić, M. Bækvad-Hansen, D. G. Baker, J. C. Beckham, L. J. Bierut, J. I. Bisson, M. P. Boks, E. A. Bolger, A. D. Børglum, B. Bradley, M. Brashear, G. Breen, R. A. Bryant, A. C. Bustamante, J. Bybjerg-Grauholm, J. R. Calabrese, J. M. Caldas- de-Almeida, A. M. Dale, M. J. Daly, N. P. Daskalakis, J. Deckert, D. L. Delahanty, M. F. Dennis, S. G. Disner, K. Domschke, A. Dzubur-Kulenovic, C. R. Erbes, A. Evans, L. A. Farrer, N. C. Feeny, J. D. Flory, D. Forbes, C. E. Franz, S. Galea, M. E. Garrett, B. Gelaye, E. Geuze, C. Gillespie, A. G. Uka, S. D. Gordon, G. Guffanti, R. Hammamieh, S. Harnal, M. A. Hauser, A. C. Heath, S. M. J. Hemmings, D. M. Hougaard, M. Jakovljevic, M. Jett, E. O. Johnson, I. Jones, T. Jovanovic, X. J. Qin, A. G. Junglen, K. I. Karstoft, M. L. Kaufman, R. C. Kessler, A. Khan, N. A. Kimbrel, A. P. King, N. Koen, H. R. Kranzler, W. S. Kremen, B. R. Lawford, L. A. M. Lebois, C. E. Lewis, S. D. Linnstaedt, A. Lori, B. Lugonja, J. J. Luykx, M. J. Lyons, J. Maples-Keller, C. Marmar, A. R. Martin, N. G. Martin, D. Maurer, M. R. Mavissakalian, A. McFarlane, R. E. McGlinchey, K. A. McLaughlin, S. A. McLean, S. McLeay, D. Mehta, W. P. Milberg, M. W. Miller, R. A. Morey, C. P. Morris, O. Mors, P. B. Mortensen, B. M. Neale, E. C. Nelson, M. Nordentoft, S. B. Norman, M. O'Donnell, H. K. Orcutt, M. S. Panizzon, E. S. Peters, A. L. Peterson, M. Peverill, R. H. Pietrzak, M. A. Polusny, J. P. Rice, S. Ripke, V. B. Risbrough, A. L. Roberts, A. O. Rothbaum, B. O. Rothbaum, P. Roy-Byrne, K. Ruggiero, A. Rung, B. P. F. Rutten, N. L. Saccone, S. E.

Sanchez, D. Schijven, S. Seedat, A. V. Seligowski, J. S. Seng, C. M. Sheerin, D. Silove, A. K. Smith, J. W. Smoller, S. R. Sponheim, D. J. Stein, J. S. Stevens, J. A. Sumner, M. H. Teicher, W. K. Thompson, E. Trapido, M. Uddin, R. J. Ursano, L. L. van den Heuvel, M. van Hooff, E. Vermetten, C. H. Vinkers, J. Voisey, Y. Wang, Z. Wang, T. Werge, M. A. Williams, D. E. Williamson, S. Winternitz, C. Wolf, E. J. Wolf, J. D. Wolff, R. Yehuda, R. M. D. Young, K. A. Young, H. Zhao, L. A. Zoellner, I. Liberzon, K. J. Ressler, M. Haas, K. C. Koenen, International meta-analysis of PTSD genome-wide association studies identifies sex- and ancestry-specific genetic risk loci. *Nat. Commun.* **10**, 4558 (2019).

106. V. Trubetskoy, A. F. Pardiñas, T. Qi, G. Panagiotaropoulou, S. Awasthi, T. B. Bigdeli, J. Bryois, C. Y. Chen, C. A. Dennison, L. S. Hall, M. Lam, K. Watanabe, O. Frei, T. Ge, J. C. Harwood, F. Koopmans, S. Magnusson, A. L. Richards, J. Sidorenko, Y. Wu, J. Zeng, J. Grove, M. Kim, Z. Li, G. Voloudakis, W. Zhang, M. Adams, I. Agartz, E. G. Atkinson, E. Agerbo, M. al Eissa, M. Albus, M. Alexander, B. Z. Alizadeh, K. Alptekin, T. D. Als, F. Amin, V. Arolt, M. Arrojo, L. Athanasiu, M. H. Azevedo, S. A. Bacanu, N. J. Bass, M. Begemann, R. A. Belliveau, J. Bene, B. Benyamin, S. E. Bergen, G. Blasi, J. Bobes, S. Bonassi, A. Braun, R. A. Bressan, E. J. Bromet, R. Bruggeman, P. F. Buckley, R. L. Buckner, J. Bybjerg-Grauholm, W. Cahn, M. J. Cairns, M. E. Calkins, V. J. Carr, D. Castle, S. V. Catts, K. D. Chambert, R. C. K. Chan, B. Chaumette, W. Cheng, E. F. C. Cheung, S. A. Chong, D. Cohen, A. Consoli, Q. Cordeiro, J. Costas, C. Curtis, M. Davidson, K. L. Davis, L. de Haan, F. Degenhardt, L. E. DeLisi, D. Demontis, F. Dickerson, D. Dikeos, T. Dinan, S. Djurovic, J. Duan, G. Ducci, F. Dudbridge, J. G. Eriksson, L. Fañanás, S. V. Faraone, A. Fiorentino, A. Forstner, J. Frank, N. B. Freimer, M. Fromer, A. Frustaci, A. Gadelha, G. Genovese, E. S. Gershon, M. Giannitelli, I. Giegling, P. Giusti-Rodríguez, S. Godard, J. I. Goldstein, J. González Peñas, A. González-Pinto, S. Gopal, J. Gratten, M. F. Green, T. A. Greenwood, O. Guillin, S. Gülöksüz, R. E. Gur, R. C. Gur, B. Gutiérrez, E. Hahn, H. Hakonarson, V. Haroutunian, A. M. Hartmann, C. Harvey, C. Hayward, F. A. Henskens, S. Herms, P. Hoffmann, D. P. Howrigan, M. Ikeda, C. Iyegbe, I. Joa, A. Julià, A. K. Kähler, T. Kam-Thong, Y. Kamatani, S. Karachanak-Yankova, O. Kebir, M. C. Keller, B. J. Kelly, A. Khrunin, S. W. Kim, J. Klovins, N. Kondratiev, B. Konte, J. Kraft, M. Kubo, V. Kučinskas, Z. A. Kučinskiene, A. Kusumawardhani, H. Kuzelova-Ptackova, S. Landi, L. C. Lazzeroni, P. H. Lee, S. E. Legge, D. S. Lehrer, R. Lencer, B. Lerer, M. Li, J. Lieberman, G. A. Light, S. Limborska, C. M. Liu, J. Lönnqvist, C. M. Loughland, J. Lubinski, J. J. Luykx, A.

Lynham, M. Macek Jr., A. Mackinnon, P. K. E. Magnusson, B. S. Maher, W. Maier, D. Malaspina, J. Mallet, S. R. Marder, S. Marsal, A. R. Martin, L. Martorell, M. Mattheisen, R. W. McCarley, C. McDonald, J. J. McGrath, H. Medeiros, S. Meier, B. Melegh, I. Melle, R. I. Mesholam-Gately, A. Metspalu, P. T. Michie, L. Milani, V. Milanova, M. Mitjans, E. Molden, E. Molina, M. D. Molto, V. Mondelli, C. Moreno, C. P. Morley, G. Muntané, K. C. Murphy, I. Myin-Germeys, I. Nenadić, G. Nestadt, L. Nikitina-Zake, C. Noto, K. H. Nuechterlein, N. L. O'Brien, F. A. O'Neill, S. Y. Oh, A. Olincy, V. K. Ota, C. Pantelis, G. N. Papadimitriou, M. Parellada, T. Paunio, R. Pellegrino, S. Periyasamy, D. O. Perkins, B. Pfuhlmann, O. Pietiläinen, J. Pimm, D. Porteous, J. Powell, D. Quattrone, D. Quested, A. D. Radant, A. Rampino, M. H. Rapaport, A. Rautanen, A. Reichenberg, C. Roe, J. L. Roffman, J. Roth, M. Rothermundt, B. P. F. Rutten, S. Saker-Delye, V. Salomaa, J. Sanjuan, M. L. Santoro, A. Savitz, U. Schall, R. J. Scott, L. J. Seidman, S. I. Sharp, J. Shi, L. J. Siever, E. Sigurdsson, K. Sim, N. Skarabis, P. Slominsky, H. C. So, J. L. Sobell, E. Söderman, H. J. Stain, N. E. Steen, A. A. Steixner-Kumar, E. Stögmann, W. S. Stone, R. E. Straub, F. Streit, E. Strengman, T. S. Stroup, M. Subramaniam, C. A. Sugar, J. Suvisaari, D. M. Svrakic, N. R. Swerdlow, J. P. Szatkiewicz, T. M. T. Ta, A. Takahashi, C. Terao, F. Thibaut, D. Toncheva, P. A. Tooney, S. Torretta, S. Tosato, G. B. Tura, B. I. Turetsky, A. Üçok, A. Vaaler, T. van Amelsvoort, R. van Winkel, J. Veijola, J. Waddington, H. Walter, A. Waterreus, B. T. Webb, M. Weiser, N. M. Williams, S. H. Witt, B. K. Wormley, J. Q. Wu, Z. Xu, R. Yolken, C. C. Zai, W. Zhou, F. Zhu, F. Zimprich, E. C. Atbaşoğlu, M. Ayub, C. Benner, A. Bertolino, D. W. Black, N. J. Bray, G. Breen, N. G. Buccola, W. F. Byerley, W. J. Chen, C. R. Cloninger, B. Crespo-Facorro, G. Donohoe, R. Freedman, C. Galletly, M. J. Gandal, M. Gennarelli, D. M. Hougaard, H. G. Hwu, A. V. Jablensky, S. A. McCarroll, J. L. Moran, O. Mors, P. B. Mortensen, B. Müller-Myhsok, A. L. Neil, M. Nordentoft, M. T. Pato, T. L. Petryshen, M. Pirinen, A. E. Pulver, T. G. Schulze, J. M. Silverman, J. W. Smoller, E. A. Stahl, D. W. Tsuang, E. Vilella, S. H. Wang, S. Xu, Indonesia Schizophrenia Consortium, N. Dai, Q. Wenwen, D. B. Wildenauer, F. Agiananda, N. Amir, R. Antoni, T. Arsianti, A. Asmarahadi, H. Diatri, P. Djatmiko, I. Irmansyah, S. Khalimah, I. Kusumadewi, P. Kusumaningrum, P. R. Lukman, M. W. Nasrun, N. S. Safyuni, P. Prasetyawan, G. Semen, K. Siste, H. Tobing, N. Widiasih, T. Wiguna, D. Wulandari, N. Evalina, A. J. Hananto, J. H. Ismoyo, T. M. Marini, S. Henuhili, M. Reza, S. Yusnadewi, PsychENCODE, A. Abyzov, S. Akbarian, A. Ashley-Koch, H. van Bakel, M. Breen, M. Brown, J. Bryois, B. Carlyle, A.

Charney, G. Coetzee, G. Crawford, S. Dracheva, P. Emani, P. Farnham, M. Fromer, T. Galeev, M. Gandal, M. Gerstein, G. Giase, K. Girdhar, F. Goes, K. Grennan, M. Gu, B. Guerra, G. Gursoy, G. Hoffman, T. Hyde, A. Jaffe, S. Jiang, Y. Jiang, A. Kefi, Y. Kim, R. Kitchen, J. A. Knowles, F. Lay, D. Lee, M. Li, C. Liu, S. Liu, E. Mattei, F. Navarro, X. Pan, M. A. Peters, D. Pinto, S. Pochareddy, D. Polioudakis, M. Purcaro, S. Purcell, H. Pratt, T. Reddy, S. Rhie, P. Roussos, J. Rozowsky, S. Sanders, N. Sestan, A. Sethi, X. Shi, A. Shieh, V. Swarup, A. Szekely, D. Wang, J. Warrell, S. Weissman, Z. Weng, K. White, J. Wiseman, H. Witt, H. Won, S. Wood, F. Wu, X. Xu, L. Yao, P. Zandi, Psychosis Endophenotypes International Consortium, M. J. Arranz, S. Bakker, S. Bender, E. Bramon, D. A. Collier, B. Crepo-Facorro, J. Hall, C. Iyegbe, R. Kahn, S. Lawrie, C. Lewis, K. Lin, D. H. Linszen, I. Mata, A. McIntosh, R. M. Murray, R. A. Ophoff, J. van Os, J. Powell, D. Rujescu, M. Walshe, M. Weisbrod, The SynGO Consortium, T. Achsel, M. Andres-Alonso, C. Bagni, À. Bayés, T. Biederer, N. Brose, T. C. Brown, J. J. E. Chua, M. P. Coba, L. N. Cornelisse, A. P. H. de Jong, J. de Juan-Sanz, D. C. Dieterich, G. Feng, H. L. Goldschmidt, E. D. Gundelfinger, C. Hoogenraad, R. L. Huganir, S. E. Hyman, C. Imig, R. Jahn, H. Jung, P. S. Kaeser, E. Kim, F. Koopmans, M. R. Kreutz, N. Lipstein, H. D. MacGillavry, R. Malenka, P. S. McPherson, V. O'Connor, R. Pielot, T. A. Ryan, D. Sahasrabudhe, C. Sala, M. Sheng, K. H. Smalla, A. B. Smit, T. C. Südhof, P. D. Thomas, R. F. Toonen, J. R. T. van Weering, M. Verhage, C. Verpelli, R. Adolfsson, C. Arango, B. T. Baune, S. I. Belangero, A. D. Børglum, D. Braff, E. Bramon, J. D. Buxbaum, D. Campion, J. A. Cervilla, S. Cichon, D. A. Collier, A. Corvin, D. Curtis, M. D. Forti, E. Domenici, H. Ehrenreich, V. Escott-Price, T. Esko, A. H. Fanous, A. Gareeva, M. Gawlik, P. V. Gejman, M. Gill, S. J. Glatt, V. Golimbet, K. S. Hong, C. M. Hultman, S. E. Hyman, N. Iwata, E. G. Jönsson, R. S. Kahn, J. L. Kennedy, E. Khusnutdinova, G. Kirov, J. A. Knowles, M. O. Krebs, C. Laurent-Levinson, J. Lee, T. Lencz, D. F. Levinson, Q. S. Li, J. Liu, A. K. Malhotra, D. Malhotra, A. McIntosh, A. McQuillin, P. R. Menezes, V. A. Morgan, D. W. Morris, B. J. Mowry, R. M. Murray, V. Nimgaonkar, M. M. Nöthen, R. A. Ophoff, S. A. Paciga, A. Palotie, C. N. Pato, S. Qin, M. Rietschel, B. P. Riley, M. Rivera, D. Rujescu, M. C. Saka, A. R. Sanders, S. G. Schwab, A. Serretti, P. C. Sham, Y. Shi, D. St Clair, H. Stefánsson, K. Stefansson, M. T. Tsuang, J. van Os, M. P. Vawter, D. R. Weinberger, T. Werge, D. B. Wildenauer, X. Yu, W. Yue, P. A. Holmans, A. J. Pocklington, P. Roussos, E. Vassos, M. Verhage, P. M. Visscher, J. Yang, D. Posthuma, O. A. Andreassen, K. S. Kendler, M. J. Owen, N. R. Wray, M. J. Daly, H. Huang,

B. M. Neale, P. F. Sullivan, S. Ripke, J. T. R. Walters, M. C. O'Donovan, Schizophrenia Working Group of the Psychiatric Genomics Consortium, L. de Haan, T. van Amelsvoort, R. van Winkel, A. Gareeva, P. C. Sham, Y. Shi, D. St Clair, J. van Os, Mapping genomic loci implicates genes and synaptic biology in schizophrenia. *Nature* **604**, 502–508 (2022).

107. D. Yu, J. H. Sul, F. Tsetsos, M. S. Nawaz, A. Y. Huang, I. Zelaya, C. Illmann, L. Osiecki, S. M. Darrow, M. E. Hirschtritt, E. Greenberg, K. R. Muller-Vahl, M. Stuhrmann, Y. Dion, G. Rouleau, H. Aschauer, M. Stamenkovic, M. Schlögelhofer, P. Sandor, C. L. Barr, M. Grados, H. S. Singer, M. M. Nöthen, J. Hebebrand, A. Hinney, R. A. King, T. V. Fernandez, C. Barta, Z. Tarnok, P. Nagy, C. Depienne, Y. Worbe, A. Hartmann, C. L. Budman, R. Rizzo, G. J. Lyon, W. M. McMahon, J. R. Batterson, D. C. Cath, I. A. Malaty, M. S. Okun, C. Berlin, D. W. Woods, P. C. Lee, J. Jankovic, M. M. Robertson, D. L. Gilbert, L. W. Brown, B. J. Coffey, A. Dietrich, P. J. Hoekstra, S. Kuperman, S. H. Zinner, P. Luðvigsson, E. Sæmundsen, Ó. Thorarensen, G. Atzmon, N. Barzilai, M. Wagner, R. Moessner, R. Ophoff, C. N. Pato, M. T. Pato, J. A. Knowles, J. L. Roffman, J. W. Smoller, R. L. Buckner, A. J. Willsey, J. A. Tischfield, G. A. Heiman, H. Stefansson, K. Stefansson, D. Posthuma, N. J. Cox, D. L. Pauls, N. B. Freimer, B. M. Neale, L. K. Davis, P. Paschou, G. Coppola, C. A. Mathews, J. M. Scharf, on behalf of the Tourette Association of America International Consortium for Genetics, the Gilles de la Tourette GWAS Replication Initiative, the Tourette International Collaborative Genetics Study, and the Psychiatric Genomics Consortium Tourette Syndrome Working Group, Interrogating the genetic determinants of Tourette's syndrome and other tic disorders through genome-wide association studies. *Am. J. Psychiatry* **176**, 217–227 (2019).

108. A. Okbay, Y. Wu, N. Wang, H. Jayashankar, M. Bennett, S. M. Nehzati, J. Sidorenko, H. Kweon, G. Goldman, T. Gjorgjieva, Y. Jiang, B. Hicks, C. Tian, D. A. Hinds, R. Ahlskog, P. K. E. Magnusson, S. Oskarsson, C. Hayward, A. Campbell, D. J. Porteous, J. Freese, P. Herd, 23andMe Research Team, M. Agee, B. Alipanahi, A. Auton, R. K. Bell, K. Bryc, S. L. Elson, P. Fontanillas, N. A. Furlotte, D. A. Hinds, K. E. Huber, A. Kleinman, N. K. Litterman, J. C. McCreight, M. H. McIntyre, J. L. Mountain, C. A. M. Northover, S. J. Pitts, J. F. Sathirapongsasuti, O. V. Sazonova, J. F. Shelton, S. Shringarpure, J. Y. Tung, V. Vacic, C. H. Wilson, Social Science Genetic Association Consortium, M. A. Fontana, T. H. Pers, C. A. Rietveld, G. B. Chen, V. Emilsson, S. F. W. Meddens, J. K. Pickrell, K. Thom, P.

Timshel, R. de Vlaming, A. Abdellaoui, T. S. Ahluwalia, J. Bacelis, C. Baumbach, G. Bjornsdottir, J. H. Brandsma, M. P. Concas, J. Derringer, T. E. Galesloot, G. Girotto, R. Gupta, L. M. Hall, S. E. Harris, E. Hofer, M. Horikoshi, J. E. Huffman, K. Kaasik, I. P. Kalafati, R. Karlsson, J. Lahti, S. J. van der Lee, C. de Leeuw, P. A. Lind, K. O. Lindgren, T. Liu, M. Mangino, J. Marten, E. Mihailov, M. B. Miller, P. J. van der Most, C. Oldmeadow, A. Payton, N. Pervjakova, W. J. Peyrot, Y. Qian, O. Raitakari, R. Rueedi, E. Salvi, B. Schmidt, K. E. Schraut, J. Shi, A. V. Smith, R. A. Poot, B. S. Pourcain, A. Teumer, G. Thorleifsson, N. Verweij, D. Vuckovic, J. Wellmann, H. J. Westra, J. Yang, W. Zhao, Z. Zhu, B. Z. Alizadeh, N. Amin, A. Bakshi, S. E. Baumeister, G. Biino, K. Bønnelykke, P. A. Boyle, H. Campbell, F. P. Cappuccio, G. Davies, J. E. de Neve, P. Deloukas, I. Demuth, J. Ding, P. Eibich, L. Eisele, N. Eklund, D. M. Evans, J. D. Faul, M. F. Feitosa, A. J. Forstner, I. Gandin, B. Gunnarsson, B. V. Halldórsson, T. B. Harris, A. C. Heath, L. J. Hocking, E. G. Holliday, G. Homuth, M. A. Horan, J. J. Hottenga, P. L. de Jager, P. K. Joshi, A. Jugessur, M. A. Kaakinen, M. Kähönen, S. Kanoni, L. Keltigangas-Järvinen, L. A. L. M. Kiemeney, I. Kolcic, S. Koskinen, A. T. Kraja, M. Kroh, Z. Kutalik, A. Latvala, L. J. Launer, M. P. Lebreton, D. F. Levinson, P. Lichtenstein, P. Lichtner, D. C. M. Liewald, LifeLines Cohort Study, A. Loukola, P. A. Madden, R. Mägi, T. Mäki-Opas, R. E. Marioni, P. Marques-Vidal, G. A. Meddens, G. McMahon, C. Meisinger, T. Meitinger, Y. Milaneschi, L. Milani, G. W. Montgomery, R. Myhre, C. P. Nelson, D. R. Nyholt, W. E. R. Ollier, A. Palotie, L. Paternoster, N. L. Pedersen, K. E. Petrovic, K. Räikkönen, S. M. Ring, A. Robino, O. Rostapshova, I. Rudan, A. Rustichini, V. Salomaa, A. R. Sanders, A. P. Sarin, H. Schmidt, R. J. Scott, B. H. Smith, J. A. Smith, J. A. Staessen, E. Steinhagen-Thiessen, K. Strauch, A. Terracciano, M. D. Tobin, S. Ulivi, S. Vaccargiu, L. Quaye, F. J. A. van Rooij, C. Venturini, A. A. E. Vinkhuyzen, U. Völker, H. Völzke, J. M. Vonk, D. Vozzi, J. Waage, E. B. Ware, G. Willemsen, J. R. Attia, D. A. Bennett, K. Berger, L. Bertram, H. Bisgaard, D. I. Boomsma, I. B. Borecki, U. Bültmann, C. F. Chabris, F. Cucca, D. Cusi, I. J. Deary, G. V. Dedoussis, C. M. van Duijn, J. G. Eriksson, B. Franke, L. Franke, P. Gasparini, P. V. Gejman, C. Gieger, H. J. Grabe, J. Gratten, P. J. F. Groenen, V. Gudnason, P. van der Harst, W. Hoffmann, E. Hyppönen, W. G. Iacono, B. Jacobsson, M. R. Järvelin, K. H. Jöckel, J. Kaprio, S. L. R. Kardia, T. Lehtimäki, S. F. Lehrer, N. G. Martin, M. McGue, A. Metspalu, N. Pendleton, B. W. J. H. Penninx, M. Perola, N. Pirastu, M. Pirastu, O. Polasek, D. Posthuma, C. Power, M. A. Province, N. J. Samani, D. Schlessinger, R. Schmidt, T. I. A. Sørensen, T. D. Spector, K.

Stefansson, U. Thorsteinsdottir, A. R. Thurik, N. J. Timpson, H. Tiemeier, A. G. Uitterlinden, V. Vitart, P. Vollenweider, D. R. Weir, J. F. Wilson, A. F. Wright, D. C. Conley, R. F. Krueger, G. D. Smith, A. Hofman, D. I. Laibson, S. E. Medland, J. Yang, T. Esko, C. Watson, J. Jala, D. Conley, P. D. Koellinger, M. Johannesson, D. Laibson, M. N. Meyer, J. J. Lee, A. Kong, L. Yengo, D. Cesarini, P. Turley, P. M. Visscher, J. P. Beauchamp, D. J. Benjamin, A. I. Young, Polygenic prediction of educational attainment within and between families from genome-wide association analyses in 3 million individuals. *Nat. Genet.* **54**, 437–449 (2022).

109. A. S. Hatoum, C. L. Morrison, E. C. Mitchell, M. Lam, C. E. Benca-Bachman, A. E. Reineberg, R. H. C. Palmer, L. M. Evans, M. C. Keller, N. P. Friedman, Genome-wide association study shows that executive functioning is influenced by GABAergic processes and is a neurocognitive genetic correlate of psychiatric disorders. *Biol. Psychiatry* **93**, 59–70 (2023).

110. J. de la Fuente, G. Davies, A. D. Grotzinger, E. M. Tucker-Drob, I. J. Deary, A general dimension of genetic sharing across diverse cognitive traits inferred from molecular data. *Nat. Hum. Behav.* **5**, 49–58 (2021).

111. H. F. Ip, C. M. van der Laan, E. M. L. Krapohl, I. Brikell, C. Sánchez-Mora, I. M. Nolte, B. St Pourcain, K. Bolhuis, T. Palviainen, H. Zafarmand, L. Colodro-Conde, S. Gordon, T. Zayats, F. Aliev, C. Jiang, C. A. Wang, G. Saunders, V. Karhunen, A. R. Hammerschlag, D. E. Adkins, R. Border, R. E. Peterson, J. A. Prinz, E. Thiering, I. Seppälä, N. Vilor-Tejedor, T. S. Ahluwalia, F. R. Day, J. J. Hottenga, A. G. Allegrini, K. Rimfeld, Q. Chen, Y. Lu, J. Martin, M. Soler Artigas, P. Rovira, R. Bosch, G. Español, J. A. Ramos Quiroga, A. Neumann, J. Ensink, K. Grasby, J. J. Morosoli, X. Tong, S. Marrington, C. Middeldorp, J. G. Scott, A. Vinkhuyzen, A. A. Shabalin, R. Corley, L. M. Evans, K. Sugden, S. Alemany, L. Sass, R. Vinding, K. Ruth, J. Tyrrell, G. E. Davies, E. A. Ehli, F. A. Hagenbeek, E. de Zeeuw, T. C. E. M. van Beijsterveldt, H. Larsson, H. Snieder, F. C. Verhulst, N. Amin, A. M. Whipp, T. Korhonen, E. Vuoksimaa, R. J. Rose, A. G. Uitterlinden, A. C. Heath, P. Madden, J. Haavik, J. R. Harris, Ø. Helgeland, S. Johansson, G. P. S. Knudsen, P. R. Njolstad, Q. Lu, A. Rodriguez, A. K. Henders, A. Mamun, J. M. Najman, S. Brown, C. Hopfer, K. Krauter, C. Reynolds, A. Smolen, M. Stallings, S. Wadsworth, T. L. Wall, J. L. Silberg, A. Miller, L.

Keltikangas-Järvinen, C. Hakulinen, L. Pulkki-Råback, A. Havdahl, P. Magnus, O. T. Raitakari, J. R. B. Perry, S. Llop, M. J. Lopez-Espinosa, K. Bønnelykke, H. Bisgaard, J. Sunyer, T. Lehtimäki, L. Arseneault, M. Standl, J. Heinrich, J. Boden, J. Pearson, L. J. Horwood, M. Kennedy, R. Poulton, L. J. Eaves, H. H. Maes, J. Hewitt, W. E. Copeland, E. J. Costello, G. M. Williams, N. Wray, M. R. Järvelin, M. McGue, W. Iacono, A. Caspi, T. E. Moffitt, A. Whitehouse, C. E. Pennell, K. L. Klump, S. A. Burt, D. M. Dick, T. Reichborn-Kjennerud, N. G. Martin, S. E. Medland, T. Vrijkotte, J. Kaprio, H. Tiemeier, G. Davey Smith, C. A. Hartman, A. J. Oldehinkel, M. Casas, M. Ribasés, P. Lichtenstein, S. Lundström, R. Plomin, M. Bartels, M. G. Nivard, D. I. Boomsma, Genetic association study of childhood aggression across raters, instruments, and age. *Transl. Psychiatry* **11**, 413 (2021).

112. J. J. Tielbeek, E. Uffelmann, B. S. Williams, L. Colodro-Conde, É. Gagnon, T. T. Mallard, B. E. Levitt, P. R. Jansen, A. Johansson, H. M. Sallis, G. Pistis, G. R. B. Saunders, A. G. Allegrini, K. Rimfeld, B. Konte, M. Klein, A. M. Hartmann, J. E. Salvatore, I. M. Nolte, D. Demontis, A. L. K. Malmberg, S. A. Burt, J. E. Savage, K. Sugden, R. Poulton, K. M. Harris, S. Vrieze, M. McGue, W. G. Iacono, N. R. Mota, J. Mill, J. F. Viana, B. L. Mitchell, J. J. Morosoli, T. F. M. Andlauer, I. Ouellet-Morin, R. E. Tremblay, S. M. Côté, J. P. Gouin, M. R. Brendgen, G. Dionne, F. Vitaro, M. K. Lupton, N. G. Martin, COGA Consortium, B. Porjesz, V. Hesselbrock, T. Foroud, A. Agrawal, H. J. Edenberg, Y. Liu, M. H. Plawecki, S. Kuperman, J. R. Kramer, J. M. Meyers, C. Kamarajan, A. Pandey, L. Bierut, J. Rice, K. K. Bucholz, M. A. Schuckit, J. Tischfield, R. Hart, L. Almasy, A. Goate, P. Slesinger, D. Scott, Spit for Science Working Group, E. Castelao, K. Räikkönen, J. G. Eriksson, J. Lahti, C. A. Hartman, A. J. Oldehinkel, H. Snieder, H. Liu, M. Preisig, A. Whipp, E. Vuoksimaa, Y. Lu, P. Jern, D. Rujescu, I. Giegling, T. Palviainen, J. Kaprio, K. P. Harden, M. R. Munafò, G. Morneau-Vaillancourt, R. Plomin, E. Viding, B. B. Boutwell, F. Aliev, D. M. Dick, A. Popma, S. V. Faraone, A. D. Børglum, S. E. Medland, B. Franke, M. Boivin, J. B. Pingault, J. C. Glennon, J. C. Barnes, S. E. Fisher, T. E. Moffitt, A. Caspi, T. J. C. Polderman, D. Posthuma, Uncovering the genetic architecture of broad antisocial behavior through a genome-wide association study meta-analysis. *Mol. Psychiatry* **27**, 4453–4463 (2022).

113. V. Warrier, R. Toro, B. Chakrabarti, iPSYCH-Broad autism group, A. D. Børglum, J. Grove, 23andMe Research Team, D. A. Hinds, T. Bourgeron, S. Baron-Cohen, Genome-wide

analyses of self-reported empathy: Correlations with autism, schizophrenia, and anorexia nervosa. *Transl. Psychiatry* **8**, 35 (2018).

114. P. Gupta, M. Galimberti, Y. Liu, S. Beck, A. Wingo, T. Wingo, K. Adhikari, H. R. Kranzler, VA Million Veteran Program, M. B. Stein, J. Gelernter, D. F. Levey, A genome-wide investigation into the underlying genetic architecture of personality traits and overlap with psychopathology. *Nat. Human Behav.* **8**, 2235–2249 (2024).

115. W. D. Hill, N. M. Davies, S. J. Ritchie, N. G. Skene, J. Bryois, S. Bell, E. di Angelantonio, D. J. Roberts, S. Xueyi, G. Davies, D. C. M. Liewald, D. J. Porteous, C. Hayward, A. S. Butterworth, A. M. McIntosh, C. R. Gale, I. J. Deary, Genome-wide analysis identifies molecular systems and 149 genetic loci associated with income. *Nat. Commun.* **10**, 5741 (2019).

116. B. M. Neale, UK Biobank GWAS Round 2 results (2018); http://www.nealelab.is/uk-biobank/.

117. K. L. Grasby, N. Jahanshad, J. N. Painter, L. Colodro-Conde, J. Bralten, D. P. Hibar, P. A. Lind, F. Pizzagalli, C. R. K. Ching, M. A. B. McMahon, N. Shatokhina, L. C. P. Zsembik, S. I. Thomopoulos, A. H. Zhu, L. T. Strike, I. Agartz, S. Alhusaini, M. A. A. Almeida, D. Alnæs, I. K. Amlien, M. Andersson, T. Ard, N. J. Armstrong, A. Ashley-Koch, J. R. Atkins, M. Bernard, R. M. Brouwer, E. E. L. Buimer, R. Bülow, C. Bürger, D. M. Cannon, M. Chakravarty, Q. Chen, J. W. Cheung, B. Couvy-Duchesne, A. M. Dale, S. Dalvie, T. K. de Araujo, G. I. de Zubicaray, S. M. C. de Zwarte, A. den Braber, N. T. Doan, K. Dohm, S. Ehrlich, H. R. Engelbrecht, S. Erk, C. C. Fan, I. O. Fedko, S. F. Foley, J. M. Ford, M. Fukunaga, M. E. Garrett, T. Ge, S. Giddaluru, A. L. Goldman, M. J. Green, N. A. Groenewold, D. Grotegerd, T. P. Gurholt, B. A. Gutman, N. K. Hansell, M. A. Harris, M. B. Harrison, C. C. Haswell, M. Hauser, S. Herms, D. J. Heslenfeld, N. F. Ho, D. Hoehn, P. Hoffmann, L. Holleran, M. Hoogman, J. J. Hottenga, M. Ikeda, D. Janowitz, I. E. Jansen, T. Jia, C. Jockwitz, R. Kanai, S. Karama, D. Kasperaviciute, T. Kaufmann, S. Kelly, M. Kikuchi, M. Klein, M. Knapp, A. R. Knodt, B. Krämer, M. Lam, T. M. Lancaster, P. H. Lee, T. A. Lett, L. B. Lewis, I. Lopes-Cendes, M. Luciano, F. Macciardi, A. F. Marquand, S. R. Mathias, T. R. Melzer, Y. Milaneschi, N. Mirza-Schreiber, J. C. V. Moreira, T. W.

Mühleisen, B. Müller-Myhsok, P. Najt, S. Nakahara, K. Nho, L. M. Olde Loohuis, D. P. Orfanos, J. F. Pearson, T. L. Pitcher, B. Pütz, Y. Quidé, A. Ragothaman, F. M. Rashid, W. R. Reay, R. Redlich, C. S. Reinbold, J. Repple, G. Richard, B. C. Riedel, S. L. Risacher, C. S. Rocha, N. R. Mota, L. Salminen, A. Saremi, A. J. Saykin, F. Schlag, L. Schmaal, P. R. Schofield, R. Secolin, C. Y. Shapland, L. Shen, J. Shin, E. Shumskaya, I. E. Sønderby, E. Sprooten, K. E. Tansey, A. Teumer, A. Thalamuthu, D. Tordesillas-Gutiérrez, J. A. Turner, A. Uhlmann, C. L. Vallerga, D. van der Meer, M. M. J. van Donkelaar, L. van Eijk, T. G. M. van Erp, N. E. M. van Haren, D. van Rooij, M. J. van Tol, J. H. Veldink, E. Verhoef, E. Walton, M. Wang, Y. Wang, J. M. Wardlaw, W. Wen, L. T. Westlye, C. D. Whelan, S. H. Witt, K. Wittfeld, C. Wolf, T. Wolfers, J. Q. Wu, C. L. Yasuda, D. Zaremba, Z. Zhang, M. P. Zwiers, E. Artiges, A. A. Assareh, R. Ayesa-Arriola, A. Belger, C. L. Brandt, G. G. Brown, S. Cichon, J. E. Curran, G. E. Davies, F. Degenhardt, M. F. Dennis, B. Dietsche, S. Djurovic, C. P. Doherty, R. Espiritu, D. Garijo, Y. Gil, P. A. Gowland, R. C. Green, A. N. Häusler, W. Heindel, B. C. Ho, W. U. Hoffmann, F. Holsboer, G. Homuth, N. Hosten, C. R. Jack Jr., M. H. Jang, A. Jansen, N. A. Kimbrel, K. Kolskår, S. Koops, A. Krug, K. O. Lim, J. J. Luykx, D. H. Mathalon, K. A. Mather, V. S. Mattay, S. Matthews, J. Mayoral van Son, S. C. McEwen, I. Melle, D. W. Morris, B. A. Mueller, M. Nauck, J. E. Nordvik, M. M. Nöthen, D. S. O'Leary, N. Opel, M. L. P. Martinot, G. B. Pike, A. Preda, E. B. Quinlan, P. E. Rasser, V. Ratnakar, S. Reppermund, V. M. Steen, P. A. Tooney, F. R. Torres, D. J. Veltman, J. T. Voyvodic, R. Whelan, T. White, H. Yamamori, H. H. H. Adams, J. C. Bis, S. Debette, C. Decarli, M. Fornage, V. Gudnason, E. Hofer, M. A. Ikram, L. Launer, W. T. Longstreth, O. L. Lopez, B. Mazoyer, T. H. Mosley, G. V. Roshchupkin, C. L. Satizabal, R. Schmidt, S. Seshadri, Q. Yang, Alzheimer's Disease Neuroimaging Initiative¶, CHARGE Consortium¶, EPIGEN Consortium¶, IMAGEN Consortium¶, SYS Consortium¶, Parkinson's Progression Markers Initiative¶, M. K. M. Alvim, D. Ames, T. J. Anderson, O. A. Andreassen, A. Arias-Vasquez, M. E. Bastin, B. T. Baune, J. C. Beckham, J. Blangero, D. I. Boomsma, H. Brodaty, H. G. Brunner, R. L. Buckner, J. K. Buitelaar, J. R. Bustillo, W. Cahn, M. J. Cairns, V. Calhoun, V. J. Carr, X. Caseras, S. Caspers, G. L. Cavalleri, F. Cendes, A. Corvin, B. Crespo-Facorro, J. C. Dalrymple-Alford, U. Dannlowski, E. J. C. de Geus, I. J. Deary, N. Delanty, C. Depondt, S. Desrivières, G. Donohoe, T. Espeseth, G. Fernández, S. E. Fisher, H. Flor, A. J. Forstner, C. Francks, B. Franke, D. C. Glahn, R. L. Gollub, H. J. Grabe, O. Gruber, A. K. Håberg, A. R. Hariri, C. A. Hartman, R. Hashimoto, A. Heinz, F. A. Henskens,

M. H. J. Hillegers, P. J. Hoekstra, A. J. Holmes, L. E. Hong, W. D. Hopkins, H. E. Hulshoff Pol, T. L. Jernigan, E. G. Jönsson, R. S. Kahn, M. A. Kennedy, T. T. J. Kircher, P. Kochunov, J. B. J. Kwok, S. le Hellard, C. M. Loughland, N. G. Martin, J. L. Martinot, C. McDonald, K. L. McMahon, A. Meyer-Lindenberg, P. T. Michie, R. A. Morey, B. Mowry, L. Nyberg, J. Oosterlaan, R. A. Ophoff, C. Pantelis, T. Paus, Z. Pausova, B. W. J. H. Penninx, T. J. C. Polderman, D. Posthuma, M. Rietschel, J. L. Roffman, L. M. Rowland, P. S. Sachdev, P. G. Sämann, U. Schall, G. Schumann, R. J. Scott, K. Sim, S. M. Sisodiya, J. W. Smoller, I. E. Sommer, B. St Pourcain, D. J. Stein, A. W. Toga, J. N. Trollor, N. J. A. van der Wee, D. van 't Ent, H. Völzke, H. Walter, B. Weber, D. R. Weinberger, M. J. Wright, J. Zhou, J. L. Stein, P. M. Thompson, S. E. Medland, Enhancing NeuroImaging Genetics through Meta-Analysis Consortium (ENIGMA)—Genetics working group, The genetic architecture of the human cerebral cortex. *Science* **367**, eaay6690 (2020).

118. E. Tissink, S. C. de Lange, J. E. Savage, D. P. Wightman, C. A. de Leeuw, K. M. Kelly, M. Nagel, M. P. van den Heuvel, D. Posthuma, Genome-wide association study of cerebellar volume provides insights into heritable mechanisms underlying brain development and mental health. *Commun. Biol.* **5**, 710 (2022).

119. E. Tissink, J. Werme, S. C. de Lange, J. E. Savage, Y. Wei, C. A. de Leeuw, M. Nagel, D. Posthuma, M. P. van den Heuvel, The genetic architectures of functional and structural connectivity properties within cerebral resting-state networks. *eNeuro* **10**, ENEURO.0242-22.2023 (2023).

120. L. Yengo, S. Vedantam, E. Marouli, J. Sidorenko, E. Bartell, S. Sakaue, M. Graff, A. U. Eliasen, Y. Jiang, S. Raghavan, J. Miao, J. D. Arias, S. E. Graham, R. E. Mukamel, C. N. Spracklen, X. Yin, S. H. Chen, T. Ferreira, H. H. Highland, Y. Ji, T. Karaderi, K. Lin, K. Lüll, D. E. Malden, C. Medina-Gomez, M. Machado, A. Moore, S. Rüeger, X. Sim, S. Vrieze, T. S. Ahluwalia, M. Akiyama, M. A. Allison, M. Alvarez, M. K. Andersen, A. Ani, V. Appadurai, L. Arbeeva, S. Bhaskar, L. F. Bielak, S. Bollepalli, L. L. Bonnycastle, J. Bork-Jensen, J. P. Bradfield, Y. Bradford, P. S. Braund, J. A. Brody, K. S. Burgdorf, B. E. Cade, H. Cai, Q. Cai, A. Campbell, M. Cañadas-Garre, E. Catamo, J. F. Chai, X. Chai, L. C. Chang, Y. C. Chang, C. H. Chen, A. Chesi, S. H. Choi, R. H. Chung, M. Cocca, M. P. Concas, C. Couture, G. Cuellar-Partida, R. Danning, E. W. Daw, F. Degenhard, G. E.

Delgado, A. Delitala, A. Demirkan, X. Deng, P. Devineni, A. Dietl, M. Dimitriou, L. Dimitrov, R. Dorajoo, A. B. Ekici, J. E. Engmann, Z. Fairhurst-Hunter, A. E. Farmaki, J. D. Faul, J. C. Fernandez-Lopez, L. Forer, M. Francescatto, S. Freitag-Wolf, C. Fuchsberger, T. E. Galesloot, Y. Gao, Z. Gao, F. Geller, O. Giannakopoulou, F. Giulianini, A. P. Gjesing, A. Goel, S. D. Gordon, M. Gorski, J. Grove, X. Guo, S. Gustafsson, J. Haessler, T. F. Hansen, A. S. Havulinna, S. J. Haworth, J. He, N. Heard-Costa, P. Hebbar, G. Hindy, Y. L. A. Ho, E. Hofer, E. Holliday, K. Horn, W. E. Hornsby, J. J. Hottenga, H. Huang, J. Huang, A. Huerta-Chagoya, J. E. Huffman, Y. J. Hung, S. Huo, M. Y. Hwang, H. Iha, D. D. Ikeda, M. Isono, A. U. Jackson, S. Jäger, I. E. Jansen, I. Johansson, J. B. Jonas, A. Jonsson, T. Jørgensen, I. P. Kalafati, M. Kanai, S. Kanoni, L. L. Kårhus, A. Kasturiratne, T. Katsuya, T. Kawaguchi, R. L. Kember, K. A. Kentistou, H. N. Kim, Y. J. Kim, M. E. Kleber, M. J. Knol, A. Kurbasic, M. Lauzon, P. le, R. Lea, J. Y. Lee, H. L. Leonard, S. A. Li, X. Li, X. Li, J. Liang, H. Lin, S. Y. Lin, J. Liu, X. Liu, K. S. Lo, J. Long, L. Lores-Motta, J. Luan, V. Lyssenko, L. P. Lyytikäinen, A. Mahajan, V. Mamakou, M. Mangino, A. Manichaikul, J. Marten, M. Mattheisen, L. Mavarani, A. F. McDaid, K. Meidtner, T. L. Melendez, J. M. Mercader, Y. Milaneschi, J. E. Miller, I. Y. Millwood, P. P. Mishra, R. E. Mitchell, L. T. Møllehave, A. Morgan, S. Mucha, M. Munz, M. Nakatochi, C. P. Nelson, M. Nethander, C. W. Nho, A. A. Nielsen, I. M. Nolte, S. S. Nongmaithem, R. Noordam, I. Ntalla, T. Nutile, A. Pandit, P. Christofidou, K. Pärna, M. Pauper, E. R. B. Petersen, L. V. Petersen, N. Pitkänen, O. Polašek, A. Poveda, M. H. Preuss, S. Pyarajan, L. M. Raffield, H. Rakugi, J. Ramirez, A. Rasheed, D. Raven, N. W. Rayner, C. Riveros, R. Rohde, D. Ruggiero, S. E. Ruotsalainen, K. A. Ryan, M. Sabater-Lleal, R. Saxena, M. Scholz, A. Sendamarai, B. Shen, J. Shi, J. H. Shin, C. Sidore, C. M. Sitlani, R. C. Slieker, R. A. J. Smit, A. V. Smith, J. A. Smith, L. J. Smyth, L. Southam, V. Steinthorsdottir, L. Sun, F. Takeuchi, D. S. P. Tallapragada, K. D. Taylor, B. O. Tayo, C. Tcheandjieu, N. Terzikhan, P. Tesolin, A. Teumer, E. Theusch, D. J. Thompson, G. Thorleifsson, P. R. H. J. Timmers, S. Trompet, C. Turman, S. Vaccargiu, S. W. van der Laan, P. J. van der Most, J. B. van Klinken, J. van Setten, S. S. Verma, N. Verweij, Y. Veturi, C. A. Wang, C. Wang, L. Wang, Z. Wang, H. R. Warren, W. Bin Wei, A. R. Wickremasinghe, M. Wielscher, K. L. Wiggins, B. S. Winsvold, A. Wong, Y. Wu, M. Wuttke, R. Xia, T. Xie, K. Yamamoto, J. Yang, J. Yao, H. Young, N. A. Yousri, L. Yu, L. Zeng, W. Zhang, X. Zhang, J. H. Zhao, W. Zhao, W. Zhou, M. E. Zimmermann, M. Zoledziewska, L. S. Adair, H. H. H. Adams, C. A. Aguilar-Salinas, F. al-Mulla, D. K. Arnett,

F. W. Asselbergs, B. O. Åsvold, J. Attia, B. Banas, S. Bandinelli, D. A. Bennett, T. Bergler, D. Bharadwaj, G. Biino, H. Bisgaard, E. Boerwinkle, C. A. Böger, K. Bønnelykke, D. I. Boomsma, A. D. Børglum, J. B. Borja, C. Bouchard, D. W. Bowden, I. Brandslund, B. Brumpton, J. E. Buring, M. J. Caulfield, J. C. Chambers, G. R. Chandak, S. J. Chanock, N. Chaturvedi, Y. D. I. Chen, Z. Chen, C. Y. Cheng, I. E. Christophersen, M. Ciullo, J. W. Cole, F. S. Collins, R. S. Cooper, M. Cruz, F. Cucca, L. A. Cupples, M. J. Cutler, S. M. Damrauer, T. M. Dantoft, G. J. de Borst, L. C. P. G. M. de Groot, P. L. de Jager, D. P. V. de Kleijn, H. Janaka de Silva, G. V. Dedoussis, A. I. den Hollander, S. du, D. F. Easton, P. J. M. Elders, A. H. Eliassen, P. T. Ellinor, S. Elmståhl, J. Erdmann, M. K. Evans, D. Fatkin, B. Feenstra, M. F. Feitosa, L. Ferrucci, I. Ford, M. Fornage, A. Franke, P. W. Franks, B. I. Freedman, P. Gasparini, C. Gieger, G. Girotto, M. E. Goddard, Y. M. Golightly, C. Gonzalez-Villalpando, P. Gordon-Larsen, H. Grallert, S. F. A. Grant, N. Grarup, L. Griffiths, V. Gudnason, C. Haiman, H. Hakonarson, T. Hansen, C. A. Hartman, A. T. Hattersley, C. Hayward, S. R. Heckbert, C. K. Heng, C. Hengstenberg, A. W. Hewitt, H. Hishigaki, C. B. Hoyng, P. L. Huang, W. Huang, S. C. Hunt, K. Hveem, E. Hyppönen, W. G. Iacono, S. Ichihara, M. A. Ikram, C. R. Isasi, R. D. Jackson, M. R. Jarvelin, Z. B. Jin, K. H. Jöckel, P. K. Joshi, P. Jousilahti, J. W. Jukema, M. Kähönen, Y. Kamatani, K. D. Kang, J. Kaprio, S. L. R. Kardia, F. Karpe, N. Kato, F. Kee, T. Kessler, A. V. Khera, C. C. Khor, L. A. L. M. Kiemeney, B. J. Kim, E. K. Kim, H. L. Kim, P. Kirchhof, M. Kivimaki, W. P. Koh, H. A. Koistinen, G. D. Kolovou, J. S. Kooner, C. Kooperberg, A. Köttgen, P. Kovacs, A. Kraaijeveld, P. Kraft, R. M. Krauss, M. Kumari, Z. Kutalik, M. Laakso, L. A. Lange, C. Langenberg, L. J. Launer, L. le Marchand, H. Lee, N. R. Lee, T. Lehtimäki, H. Li, L. Li, W. Lieb, X. Lin, L. Lind, A. Linneberg, C. T. Liu, J. Liu, M. Loeffler, B. London, S. A. Lubitz, S. J. Lye, D. A. Mackey, R. Mägi, P. K. E. Magnusson, G. M. Marcus, P. M. Vidal, N. G. Martin, W. März, F. Matsuda, R. W. McGarrah, M. McGue, A. J. McKnight, S. E. Medland, D. Mellström, A. Metspalu, B. D. Mitchell, P. Mitchell, D. O. Mook-Kanamori, A. D. Morris, L. A. Mucci, P. B. Munroe, M. A. Nalls, S. Nazarian, A. E. Nelson, M. J. Neville, C. Newton-Cheh, C. S. Nielsen, M. M. Nöthen, C. Ohlsson, A. J. Oldehinkel, L. Orozco, K. Pahkala, P. Pajukanta, C. N. A. Palmer, E. J. Parra, C. Pattaro, O. Pedersen, C. E. Pennell, B. W. J. H. Penninx, L. Perusse, A. Peters, P. A. Peyser, D. J. Porteous, D. Posthuma, C. Power, P. P. Pramstaller, M. A. Province, Q. Qi, J. Qu, D. J. Rader, O. T. Raitakari, S. Ralhan, L. S. Rallidis, D. C. Rao, S. Redline, D. F. Reilly, A. P. Reiner, S. Y. Rhee, P. M. Ridker, M. Rienstra, S. Ripatti, M.

D. Ritchie, D. M. Roden, F. R. Rosendaal, J. I. Rotter, I. Rudan, F. Rutters, C. Sabanayagam, D. Saleheen, V. Salomaa, N. J. Samani, D. K. Sanghera, N. Sattar, B. Schmidt, H. Schmidt, R. Schmidt, M. B. Schulze, H. Schunkert, L. J. Scott, R. J. Scott, P. Sever, E. J. Shiroma, M. B. Shoemaker, X. O. Shu, E. M. Simonsick, M. Sims, J. R. Singh, A. B. Singleton, M. F. Sinner, J. G. Smith, H. Snieder, T. D. Spector, M. J. Stampfer, K. J. Stark, D. P. Strachan, L. M. 't Hart, Y. Tabara, H. Tang, J. C. Tardif, T. A. Thanaraj, N. J. Timpson, A. Tönjes, A. Tremblay, T. Tuomi, J. Tuomilehto, M. T. Tusié-Luna, A. G. Uitterlinden, R. M. van Dam, P. van der Harst, N. van der Velde, C. M. van Duijn, N. M. van Schoor, V. Vitart, U. Völker, P. Vollenweider, H. Völzke, N. H. Wacher-Rodarte, M. Walker, Y. X. Wang, N. J. Wareham, R. M. Watanabe, H. Watkins, D. R. Weir, T. M. Werge, E. Widen, L. R. Wilkens, G. Willemsen, W. C. Willett, J. F. Wilson, T. Y. Wong, J. T. Woo, A. F. Wright, J. Y. Wu, H. Xu, C. S. Yajnik, M. Yokota, J. M. Yuan, E. Zeggini, B. S. Zemel, W. Zheng, X. Zhu, J. M. Zmuda, A. B. Zonderman, J. A. Zwart, 23andMe Research Team, G. C. Partida, VA Million Veteran Program, Y. Sun, DiscovEHR (DiscovEHR and MyCode Community Health Initiative), eMERGE (Electronic Medical Records and Genomics Network), D. Croteau-Chonka, Lifelines Cohort Study, J. M. Vonk, The PRACTICAL Consortium, S. Chanock, L. le Marchand, Understanding Society Scientific Group, D. I. Chasman, Y. S. Cho, I. M. Heid, M. I. McCarthy, M. C. Y. Ng, C. J. O'Donnell, F. Rivadeneira, U. Thorsteinsdottir, Y. V. Sun, E. S. Tai, M. Boehnke, P. Deloukas, A. E. Justice, C. M. Lindgren, R. J. F. Loos, K. L. Mohlke, K. E. North, K. Stefansson, R. G. Walters, T. W. Winkler, K. L. Young, P. R. Loh, J. Yang, T. Esko, T. L. Assimes, A. Auton, G. R. Abecasis, C. J. Willer, A. E. Locke, S. I. Berndt, G. Lettre, T. M. Frayling, Y. Okada, A. R. Wood, P. M. Visscher, J. N. Hirschhorn, A saturated map of common genetic variants associated with human height. *Nature* **610**, 704–712 (2022).

121. S. Dalvie, A. X. Maihofer, J. R. I. Coleman, B. Bradley, G. Breen, L. A. Brick, C. Y. Chen, K. W. Choi, L. E. Duncan, G. Guffanti, M. Haas, S. Harnal, I. Liberzon, N. R. Nugent, A. C. Provost, K. J. Ressler, K. Torres, A. B. Amstadter, S. Bryn Austin, D. G. Baker, E. A. Bolger, R. A. Bryant, J. R. Calabrese, D. L. Delahanty, L. A. Farrer, N. C. Feeny, J. D. Flory, D. Forbes, S. Galea, A. Gautam, J. Gelernter, R. Hammamieh, M. Jett, A. G. Junglen, M. L. Kaufman, R. C. Kessler, A. Khan, H. R. Kranzler, L. A. M. Lebois, C. Marmar, M. R. Mavissakalian, A. McFarlane, M. O'Donnell, H. K. Orcutt, R. H. Pietrzak, V. B. Risbrough, A. L. Roberts, A. O. Rothbaum, P. Roy-Byrne, K. Ruggiero, A. V. Seligowski, C. M.

Sheerin, D. Silove, J. W. Smoller, M. B. Stein, M. H. Teicher, R. J. Ursano, M. van Hooff, S. Winternitz, J. D. Wolff, R. Yehuda, H. Zhao, L. A. Zoellner, D. J. Stein, K. C. Koenen, C. M. Nievergelt, Genomic influences on self-reported childhood maltreatment. *Transl. Psychiatry* **10**, 38 (2020).

122. R. S. Gisladottir, A. Helgason, B. V. Halldorsson, H. Helgason, M. Borsky, Y. R. Chien, J. Gudnason, S. A. Gudjonsson, S. Moisik, D. Dediu, G. Thorleifsson, V. Tragante, M. Bustamante, G. A. Jonsdottir, L. Stefansdottir, G. Rutsdottir, S. H. Magnusson, M. Hardarson, E. Ferkingstad, G. H. Halldorsson, S. Rognvaldsson, A. Skuladottir, E. V. Ivarsdottir, G. Norddahl, G. Thorgeirsson, I. Jonsdottir, M. O. Ulfarsson, H. Holm, H. Stefansson, U. Thorsteinsdottir, D. F. Gudbjartsson, P. Sulem, K. Stefansson, Sequence variants affecting voice pitch in humans. *Sci. Adv.* **9**, eabq2969 (2023).

123. A. R. Quinlan, I. M. Hall, BEDTools: A flexible suite of utilities for comparing genomic features. *Bioinformatics* **26**, 841–842 (2010).

124. E. S. Davis, W. Mu, S. Lee, M. G. Dozmorov, M. I. Love, D. H. Phanstiel, matchRanges: Generating null hypothesis genomic ranges via covariate-matched sampling. *Bioinformatics* **39**, btad197 (2023).

125. R. M. Kuhn, D. Haussler, W. J. Kent, The UCSC genome browser and associated tools. *Brief. Bioinform.* **14**, 144–161 (2013).

126. T. R. Thomas, A. J. Tener, A. M. Pearlman, K. L. Imborek, J. S. Yang, J. F. Strang, J. J. Michaelson, Polygenic scores clarify the relationship between mental health and gender diversity. *Biol. Psychiatry Glob. Open Scie.* **4**, 100291 (2024).

127. L. G. Casten, T. Koomar, M. Elsadany, C. M. Kone, B. Tysseling, M. Sasidharan, J. Bruce Tomblin, J. J. Michaelson, Lingo: An automated, web-based deep phenotyping platform for language ability. medRxiv:2024.03.29.24305034 (2024).

128. K. Prüfer, F. Racimo, N. Patterson, F. Jay, S. Sankararaman, S. Sawyer, A. Heinze, G. Renaud, P. H. Sudmant, C. de Filippo, H. Li, S. Mallick, M. Dannemann, Q. Fu, M. Kircher, M. Kuhlwilm, M. Lachmann, M. Meyer, M. Ongyerth, M. Siebauer, C. Theunert, A.

Tandon, P. Moorjani, J. Pickrell, J. C. Mullikin, S. H. Vohr, R. E. Green, I. Hellmann, P. L. F. Johnson, H. Blanche, H. Cann, J. O. Kitzman, J. Shendure, E. E. Eichler, E. S. Lein, T. E. Bakken, L. V. Golovanova, V. B. Doronichev, M. V. Shunkov, A. P. Derevianko, B. Viola, M. Slatkin, D. Reich, J. Kelso, S. Pääbo, The complete genome sequence of a Neanderthal from the Altai Mountains. *Nature* **505**, 43–49 (2014).

129. F. Mafessoni, S. Grote, C. de Filippo, V. Slon, K. A. Kolobova, B. Viola, S. V. Markin, M. Chintalapati, S. Peyrégne, L. Skov, P. Skoglund, A. I. Krivoshapkin, A. P. Derevianko, M. Meyer, J. Kelso, B. Peter, K. Prüfer, S. Pääbo, A high-coverage Neandertal genome from Chagyrskaya Cave. *Proc. Natl. Acad. Sci. U.S.A.* **117**, 15132–15136 (2020).

130. K. Prüfer, C. de Filippo, S. Grote, F. Mafessoni, P. Korlević, M. Hajdinjak, B. Vernot, L. Skov, P. Hsieh, S. Peyrégne, D. Reher, C. Hopfe, S. Nagel, T. Maricic, Q. Fu, C. Theunert, R. Rogers, P. Skoglund, M. Chintalapati, M. Dannemann, B. J. Nelson, F. M. Key, P. Rudan, Ž. Kućan, I. Gušić, L. V. Golovanova, V. B. Doronichev, N. Patterson, D. Reich, E. E. Eichler, M. Slatkin, M. H. Schierup, A. M. Andrés, J. Kelso, M. Meyer, S. Pääbo, A high-coverage Neandertal genome from Vindija Cave in Croatia. *Science* **358**, 655–658 (2017).

131. M. Meyer, M. Kircher, M. T. Gansauge, H. Li, F. Racimo, S. Mallick, J. G. Schraiber, F. Jay, K. Prüfer, C. de Filippo, P. H. Sudmant, C. Alkan, Q. Fu, R. do, N. Rohland, A. Tandon, M. Siebauer, R. E. Green, K. Bryc, A. W. Briggs, U. Stenzel, J. Dabney, J. Shendure, J. Kitzman, M. F. Hammer, M. V. Shunkov, A. P. Derevianko, N. Patterson, A. M. Andrés, E. E. Eichler, M. Slatkin, D. Reich, J. Kelso, S. Pääbo, A high-coverage genome sequence from an archaic Denisovan individual. *Science* **338**, 222–226 (2012).

132. J. Friedman, T. Hastie, R. Tibshirani, Regularization paths for generalized linear models via coordinate descent. *J. Stat. Softw.* **33**, 1–22 (2010).

133. D. York, N. M. Evensen, M. L. Martı́nez, J. De Bersabe Delgado, Unified equations for the slope, intercept, and standard errors of the best straight line. *Am. J. Phys.* **72**, 367–375 (2004).

134. T. Paysan-Lafosse, M. Blum, S. Chuguransky, T. Grego, B. L. Pinto, G. A. Salazar, M. L. Bileschi, P. Bork, A. Bridge, L. Colwell, J. Gough, D. H. Haft, I. Letunić, A. Marchler-

Bauer, H. Mi, D. A. Natale, C. A. Orengo, A. P. Pandurangan, C. Rivoire, C. J. A. Sigrist, I. Sillitoe, N. Thanki, P. D. Thomas, S. C. E. Tosatto, C. H. Wu, A. Bateman, InterPro in 2022. *Nucleic Acids Res.* **51**, D418–D427 (2023).

135. D. Szklarczyk, K. Nastou, M. Koutrouli, R. Kirsch, F. Mehryary, R. Hachilif, D. Hu, M. E. Peluso, Q. Huang, T. Fang, N. T. Doncheva, S. Pyysalo, P. Bork, L. J. Jensen, C. von Mering, The STRING database in 2025: Protein networks with directionality of regulation. *Nucleic Acids Res.* **53**, D730–D737 (2025).

136. A. Akbari, A. R. Barton, S. Gazal, Z. Li, M. Kariminejad, A. Perry, Y. Zeng, A. Mittnik, N. Patterson, M. Mah, X. Zhou, A. L. Price, E. S. Lander, R. Pinhasi, N. Rohland, S. Mallick, D. Reich, Pervasive findings of directional selection realize the promise of ancient DNA to elucidate human adaptation. bioRxiv:2024.09.14.613021 (2024).

137. S. Purcell, B. Neale, K. Todd-Brown, L. Thomas, M. A. R. Ferreira, D. Bender, J. Maller, P. Sklar, P. I. W. de Bakker, M. J. Daly, P. C. Sham, PLINK: A tool set for whole-genome association and population-based linkage analyses. *Am. J. Human Genet.* **81**, 559–575 (2007).

138. J. Yang, S. H. Lee, M. E. Goddard, P. M. Visscher, GCTA: A tool for genome-wide complex trait analysis. *Am. J. Human Genet.* **88**, 76–82 (2011).

139. H. Perdry, C. Dandine-Roulland, gaston: Genetic Data Handling (QC, GRM, LD, PCA) and Linear Mixed Models, R package version 1.6 (2023); https://CRAN.R-project.org/package=gaston.

140. N. R. Karcher, D. M. Barch, The ABCD study: Understanding the development of risk for mental and physical health outcomes. *Neuropsychopharmacology* **46**, 131–142 (2021).

141. D. Risso, J. Ngai, T. P. Speed, S. Dudoit, Normalization of RNA-seq data using factor analysis of control genes or samples. *Nat. Biotechnol.* **32**, 896–902 (2014).

142. A. Tenenhaus, C. Philippe, V. Guillemot, K. A. le Cao, J. Grill, V. Frouin, Variable selection for generalized canonical correlation analysis. *Biostatistics* **15**, 569–583 (2014).

143. E. H. Au, C. Fauci, Y. Luo, R. J. Mangan, D. A. Snellings, C. R. Shoben, S. Weaver, S. K. Simpson, C. B. Lowe, Gonomics: Uniting high performance and readability for genomics with Go. *Bioinformatics* **39**, btad516 (2023).

144. P. J. A. Cock, T. Antao, J. T. Chang, B. A. Chapman, C. J. Cox, A. Dalke, I. Friedberg, T. Hamelryck, F. Kauff, B. Wilczynski, M. J. L. de Hoon, Biopython: Freely available Python tools for computational molecular biology and bioinformatics. *Bioinformatics* **25**, 1422–1423 (2009).
